# Reunderstanding the classical prescription Banxia Xiexin Decoction: new perspectives from a comprehensive review of clinical research and pharmacological studies

**DOI:** 10.1186/s13020-025-01087-0

**Published:** 2025-03-18

**Authors:** Chang Liu, Pengwei Gao, Xiaoying Liu, Min Kuang, Haoran Xu, Yangming Wu, Wenjun Liu, Shengpeng Wang

**Affiliations:** 1 State Key Laboratory of Quality Research in Chinese Medicine, Institute of Chinese Medical Sciences, University of Macau, Taipa, Macao China; 2 Macao Centre for Research and Development in Chinese Medicine, Institute of Chinese Medical Sciences, University of Macau, Taipa, Macao China; 3 State Key Laboratory for the Modernization of Classical and Famous Prescriptions of Chinese Medicine, Nanchang, China; 4 Research and Development Department, Jiangzhong Pharmaceutical Co., Ltd., Nanchang, China

**Keywords:** Banxia Xiexin Decoction, Classical prescription, Gastric ulcer, Gastritis, Bibliometric analysis

## Abstract

Classical prescriptions of Chinese medicine represent the crystallized wisdom of millennia of clinical practice, enduring as cornerstones of therapeutic intervention due to their demonstrated efficacy across generations. Their evolving role in modern healthcare systems reflects shifting disease patterns, scientific advancements, and global health priorities. Banxia Xiexin Decoction (BXD), formulated by Zhang Zhongjing in the *Treatise on Febrile and Miscellaneous Diseases* (*Shanghanlun*), is a time-honored classical prescription renowned for its therapeutic versatility in managing gastrointestinal disorders, both in China and internationally. Recent advancements in clinical research and pharmacological studies on BXD underscore the necessity for a comprehensive bibliometric analysis to summarize and elucidate its specific clinical benefits. Through an extensive literature review of publications from the Web of Science, PubMed, Scopus, and the China National Knowledge Infrastructure (CNKI) between 1997 and 2024, 11 major categories of clinical applications for BXD were identified, along with an analysis of the potential pharmacological mechanisms, such as chronic gastritis, functional dyspepsia, and inflammatory bowel disease. We believe this review will provide new insights into the understanding of clinical value of BXD and identify potential future perspectives for its research and development.

**Supplementary Information:**

The online version contains supplementary material available at 10.1186/s13020-025-01087-0.

## Introduction

Classical prescriptions of Chinese medicine are one of the primary carriers that embody the holistic perspective and individualized treatment of traditional Chinese medicine (TCM), which are quintessential aspects of TCM wisdom. Particularly, classical prescriptions exemplified by those formulated by Zhang Zhongjing in the Han Dynasty serve as foundational remedies for treating diseases, and their unique therapeutic effects have been validated over millennia. However, with the increasing precision in medical treatment, the development of classical prescriptions encounters challenges such as difficulties in quality control, unclear mechanisms of action, and undefined clinical positioning. Among these, clinical value is the core prerequisite and driving force for the development of classical prescriptions, as well as the guide for whole-process quality control. Therefore, it is necessary to conduct systematic literature review and analysis to identify and analyze the clinical advantages of classical prescriptions, thereby providing precise guidance for their clinical application.

Banxia Xiexin Decoction (BXD), originating from the *Treatise on Febrile and Miscellaneous Diseases* (*Shanghanlun*), is primarily used to treat “Xinxiapi,” a condition characterized by fullness and tightness in the epigastrium that is soft and non-painful upon pressure. Historical records indicate that BXD has been employed to treat a variety of ailments, with the most common being spleen and stomach system disorders. Its applications extend to hepatobiliary diseases, lung diseases, kidney diseases, heart diseases, brain diseases, exogenous diseases, and surgical conditions, encompassing a wide range of clinical issues. Since the Tang Dynasty (618 A.D.–907 A.D.), BXD has been utilized for treating cholera, dysentery, diarrhea, among other conditions. By the Ming (1368 A.D.–1644 A.D.) and Qing (1616 A.D.–1912 A.D.) Dynasties, the use of BXD reached its zenith in treating ailments such as diarrhea and jaundice, further expanding its therapeutic scope [1]. BXD has also been widely used in Japan as a Kampo medicine, specifically as Hangeshashinto, since the sixteenth century, due to its beneficial effects on gastritis, inflammatory diarrhea, and oral mucositis [2].

With the continuous deepening of the clinical application of BXD, its therapeutic scope has expanded beyond the primary symptoms it was originally designed to address. BXD is now widely used to treat and alleviate a variety of conditions, including gastric ulcers, ulcerative colitis, Helicobacter pylori infection, polycystic ovary syndrome, diabetic gastroparesis, insulin resistance, gastrointestinal cancer, and other diseases [1]. This expansion goes far beyond the original scope of treatment as described in the *Treatise on Febrile Diseases and Miscellaneous Diseases.* The broadened applications of BXD not only enhance its clinical utility but also offer new perspectives for researchers in the utilization of classical prescriptions.

The publication of “Catalogue of Ancient Classical Prescriptions (First Batch)” specifies the composition and usage of BXD, in which BXD consists of 7 herbs with the following dosages: 0.5 *sheng* of Pinelliae Rhizoma (Banxia), 3 *liang* of Scutellariae Radix (Huangqin), 3 *liang* of Zingiberis Rhizoma (Ganjiang), 3 *liang* of Glycyrrhizae Radix et Rhizoma Praeparata cum Melle (Gancao) and Ginseng Radix et Rhizoma (Renshen), 1 *liang* of Coptidis Rhizoma (Huanglian), and 12 counts of Jujubae Fructus (Dazao) in the preparation of the decoction [3].

Recent advancements in clinical research and pharmacological studies on BXD highlight the necessity for a comprehensive bibliometric analysis to summarize and elucidate its specific clinical benefits. To achieve this, this review is designed to first employ bibliometric analysis to summarize the articles in the field of global trends in BXD research and development, encompassing temporal distribution and spatial coordination. Subsequently, based on the screened literature, we categorized current clinical studies into 11 categories according to the practical applications of BXD and analyzed the corresponding pharmacological mechanisms. This approach aims to provide a comprehensive overview of the field, highlight current research hotspots, and suggest future directions for BXD research.

## Chemical composition and quality control of BXD

BXD, a prescription comprising 7 Chinese herbal medicines, namely Pinelliae Rhizoma, Coptidis Rhizoma, Scutellariae Radix, Zingiberis Rhizoma, Glycyrrhizae Radix et Rhizoma, Jujubae Fructus, and Ginseng Radix et Rhizoma, exhibits a complex and multifaceted chemical composition. A thorough understanding of the constituents of these medicinal herbs is essential for both the quality control and the elucidation of their pharmacological mechanisms.

**Fig. 1:**
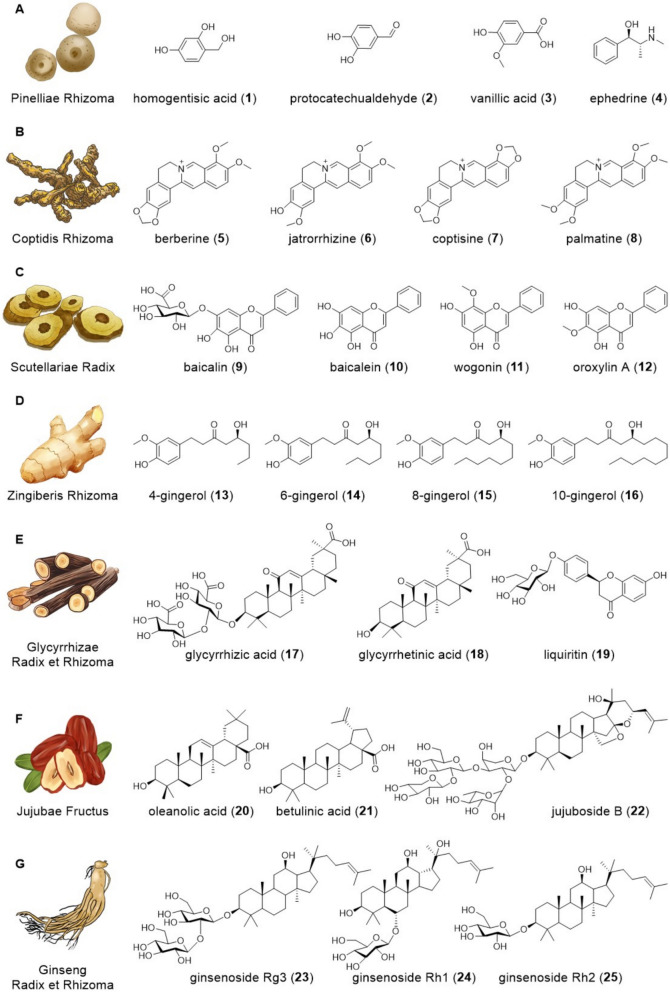
Seven Chinese herbal medicines comprising BXD and their representative compounds. Representative active compounds of Pinelliae Rhizoma (**A**), Coptidis Rhizoma (**B**), Scutellariae Radix (**C**), Zingiberis Rhizoma (**D**), Glycyrrhizae Radix et Rhizoma (**E**), Jujubae Fructus (**F**), and Ginseng Radix et Rhizoma (**G**)

Pinelliae Rhizoma, the principal herb of the prescription, is the dried tuber of *Pinellia ternata* (Thunb.) Breit [4]. It mainly contains organic acids, nucleosides, flavonoids, alkaloids. Organic acids have been identified as the bioactive constituents from *P. ternata* (Thunb.) Breit, and the content is positively correlated with the antitussive effect, such as homogentisic acid (**1**), protocatechualdehyde (**2**) and vanillic acid (**3**) [4]. Alkaloids are generally considered to be the main bioactive ingredients of Pinelliae Rhizoma and exert important pharmacological effects. The ephedrine (**4**) is the first alkaloid isolated and identified as the active principle of Pinelliae Rhizoma (Fig. 1A) [5]. Coptidis Rhizoma, the dried rhizome of *Coptis chinensis* Franch., *Coptis deltoidei* C. Y. Cheng et Hsiao, or *Coptis teeta* Wall, is primarily characterized by its content of alkaloids, including notable compounds such as berberine (**5**), jatrorrhizine (**6**), coptisine (**7**) and palmatine (**8**) (Fig. 1B) [6]. Scutellariae Radix is the dried root of *Scutellaria baicalensis* Georgi, and its flavonoids, especially baicalin (**9**), baicalein (**10**), wogonin (**11**) and oroxylin A (**12**) (Fig. 1C), are widely recognized as its principal bioactive constituents [7]. Zingiberis Rhizoma, also known as dry ginger, is the dried rhizome of *Zingiber officinale* Roscoe, commonly used as both a medicinal and dietary additive. A variety of active components have been identified in dry ginger, with 4-gingerol (**13**), 6-gingerol (**14**), 8-gingerol (**15**) and 10-gingerol (**16**) (Fig. 1D) being extensively studied for their medicinal properties [8]. Glycyrrhizae Radix et Rhizoma, commonly known as licorice, is the dry root and rhizome of *Glycyrrhiza uralensis* Fisch., *Glycyrrhiza inflata* Bat, or *Glycyrrhiza glabra* L. It mainly contains flavonoid glycosides, triterpenoid saponins, and phenolic compounds, such as glycyrrhizic acid (**17**), glycyrrhetinic acid (**18**) and liquiritin (**19**) (Fig. 1E) [9]. Jujubae Fructus, commonly referred to as jujube, is the dry ripe fruit of *Ziziphus jujuba* Mill. The principal biologically active constituents of Jujubae Fructus encompass cyclic adenosine monophosphate, phenolics, flavonoids, triterpenic acids, and polysaccharides, among which oleanolic acid (**20**), betulinic acid (**21**) and jujuboside B (**22**) are notable (Fig. 1F) [10]. Ginseng Radix et Rhizoma, the rhizome and root of *Panax ginseng* C. A. Meyer, contains a variety of active constituents, including ginsenosides, volatile oils, amino acids, peptides, polysaccharides, nitrogen compounds and polyacetylenes. Among these, ginseng saponins such as ginsenosides Rg_3_ (**23**), Rh_1_ (**24**) and Rh_2_ (**25**) (Fig. 1G), have been identified as the principal and most active constituents [11, 12].

However, considering the chemical reactions that may exist in the decoction process, the chemical constituents of BXD cannot be regarded as a simple superposition of the constituents of the seven Chinese herbal medicines. Combining chemical compositional studies of individual herbs and prescription, the main chemical constituents of BXD were found to be alkaloids, flavonoids, and saponins [13]. The complexity and diversity of the chemical composition of BXD present significant challenges for the quality control of this prescription. As of the latest edition, the Chinese Pharmacopoeia does not provide specific guidelines for the quality control of BXD. However, the Japanese Pharmacopoeia provides detailed specifications for the quality control of BXD (Hangeshashinto). For qualitative analysis, thin-layer chromatography (TLC) is used for the identification of six herbs, including Scutellariae Radix, licorice, processed ginger, ginger, ginseng, and Coptidis Rhizoma. In terms of content limits, baicalin, glycyrrhizic acid, and berberine are used to establish limits for Scutellariae Radix, licorice, and Coptidis Rhizoma. Additionally, specifications for heavy metals, arsenic, loss on drying, and total ash are also included. However, the Japanese Pharmacopoeia does not provide qualitative or quantitative analysis for Pinelliae Rhizoma and jujube [14].

With the rapid development of modern analysis technology, many researchers established qualitative and quantitative detection methods for BXD. 18 active components in BXD are accurately quantitated by a validated UPLC-MS/MS method, including liquiritin, coptisine, jatrorrhizine, scutellarin, berberine, palmatine, isoliquiritin, liquiritigenin, baicalin, wogonoside, ginsenoside Rg_1_, ginsenoside Re, isoliquiritigenin, baicalein, wogonin, oroxylin A, glycyrrhizic acid and ginsenoside Rb_1_ [13]. By using UPLC/Q-TOF-MS, a total of 74 compounds are detected and characterized in BXD, including flavonoids, triterpenoid saponins, alkaloids and glycosides [15]. In addition, TLC and DESI-MSI are also applied in the quality control and chemical composition study of BXD, as well as 15 batches BXD are used to establish the HPLC-fingerprints [16, 17].

## Bibliometric analysis

Bibliometric analysis, introduced by Pritchard in 1969 [18], uses statistical methods to evaluate publication trends, authorship patterns, and research impacts across scientific fields [19]. Tools like CiteSpace and VOSviewer are widely employed for such analyses. CiteSpace identifies emerging research trends and visualizes knowledge domains over time [20, 21], while VOSviewer maps influential journals, authors, and collaborative networks [22]. Both tools will be applied here to analyze evolving trends and emerging directions in BXD research.

### Data source, screening and collection

**Fig. 2:**
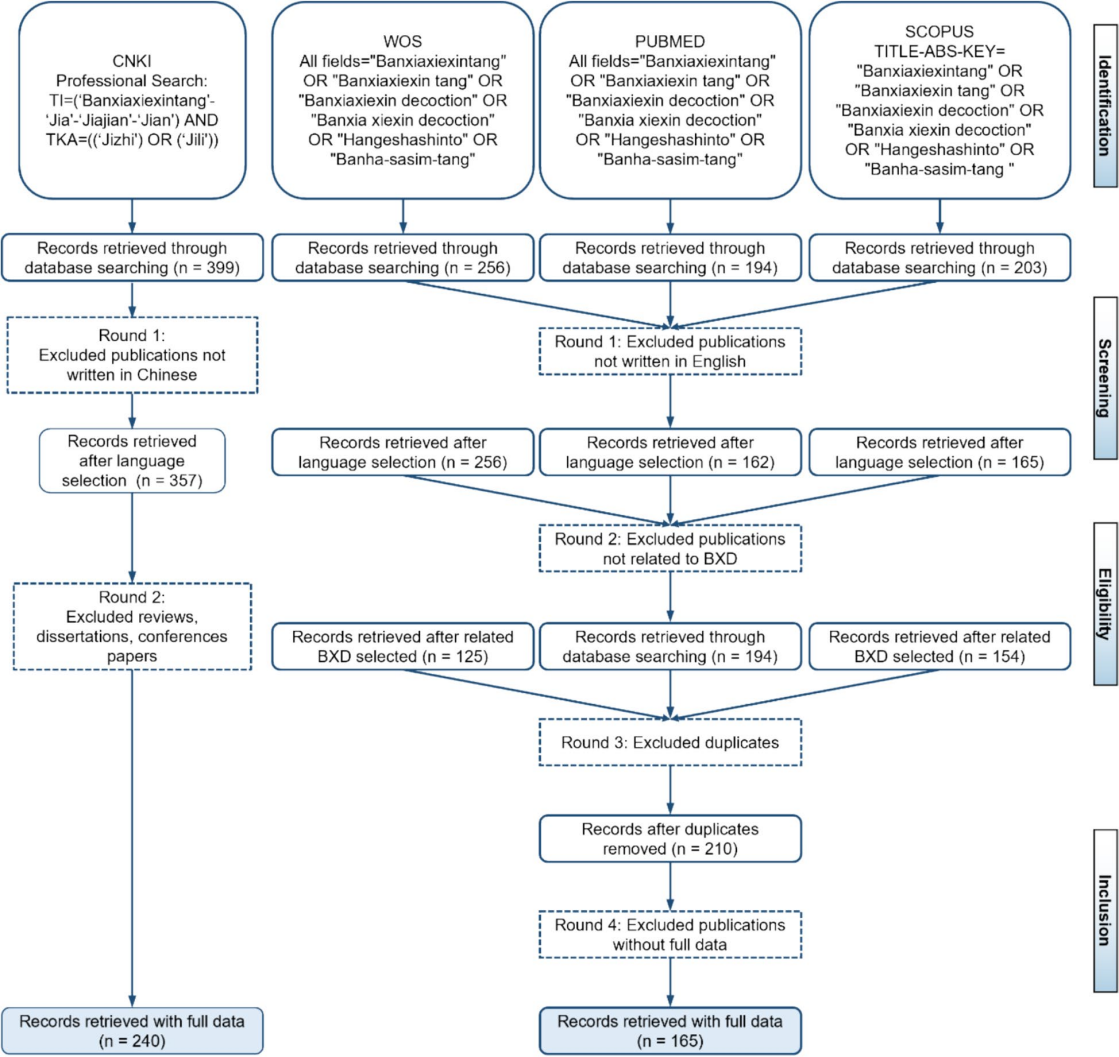
Screening process for publications of BXD using CNKI, WoS, PubMed, and Scopus databases

Following PRISMA guidelines, data were retrieved from Web of Science (WoS), PubMed, Scopus, and CNKI, with separate strategies for WOS/PubMed/Scopus and CNKI due to language differences (CNKI contains Chinese articles). For WOS, PubMed, and Scopus, variations of “Banxia Xiexin Decoction” (e.g., “Banxiaxiexintang,” “Hangeshashinto,” “Banha-sasim-tang”) were searched in titles, abstracts, and keywords. Inclusion criteria included English-language publications, high relevance to BXD, sufficient data for analysis, and publications from 1997 (earliest BXD record) to June 2024, with no restrictions on gender, age, or ethnicity. The screening process began with 653 articles, narrowed to 583 after keyword screening, and further reduced to 473, 210, and finally 165 eligible articles (Fig. 2). These 165 articles were exported in “Full record and Cited References” (Plain Text) format for CiteSpace analysis, including titles, authors, abstracts, citations, and publication dates.

To capture the extensive research history of BXD, first documented in the *Treatise on Febrile and Miscellaneous Diseases* (Eastern Han Dynasty), this study also incorporates data from the China National Knowledge Infrastructure (CNKI), the largest Chinese-language academic database covering > 99% of Chinese journals [23]. CNKI searches used the terms TI = (‘Banxiaxiexintang’-‘Jia’-‘Jiajian’-‘Jian’) AND TKA = ((‘Jizhi’) OR (‘Jili’)) to retrieve articles (1997–2024) meeting criteria: (1) Chinese language; (2) focus on BXD’s pharmacological mechanisms; (3) sufficient data for analysis. Initial screening identified 399 articles, reduced to 240 eligible entries after excluding non-Chinese publications, reviews, dissertations, and conference papers (Fig. 2). These were formatted for CiteSpace to analyze BXD’s research dynamics comprehensively.

### Data visualization and data analysis

We further employed CiteSpace (v6.3.R1) and VOSviewer for bibliometric analysis to map research trends and collaborations in BXD studies. CiteSpace, configured with a 1997–2024 timeframe, annual time slices, and node types (country/institution/keywords), identifies research hotspots and emerging themes through co-authorship, co-citation, and co-occurrence analyses [24]. These metrics reveal collaborative networks (authors/countries/institutions), conceptual linkages (keyword clusters), and intellectual influences (cited references). VOSviewer complements this by visualizing knowledge domain structures and collaboration density [25]. Using 405 screened publications, temporal trends were assessed via annual publication counts and keyword evolution, while spatial cooperation networks highlighted global and institutional collaboration patterns.

#### Time distribution and keyword clusters

**Fig. 3:**
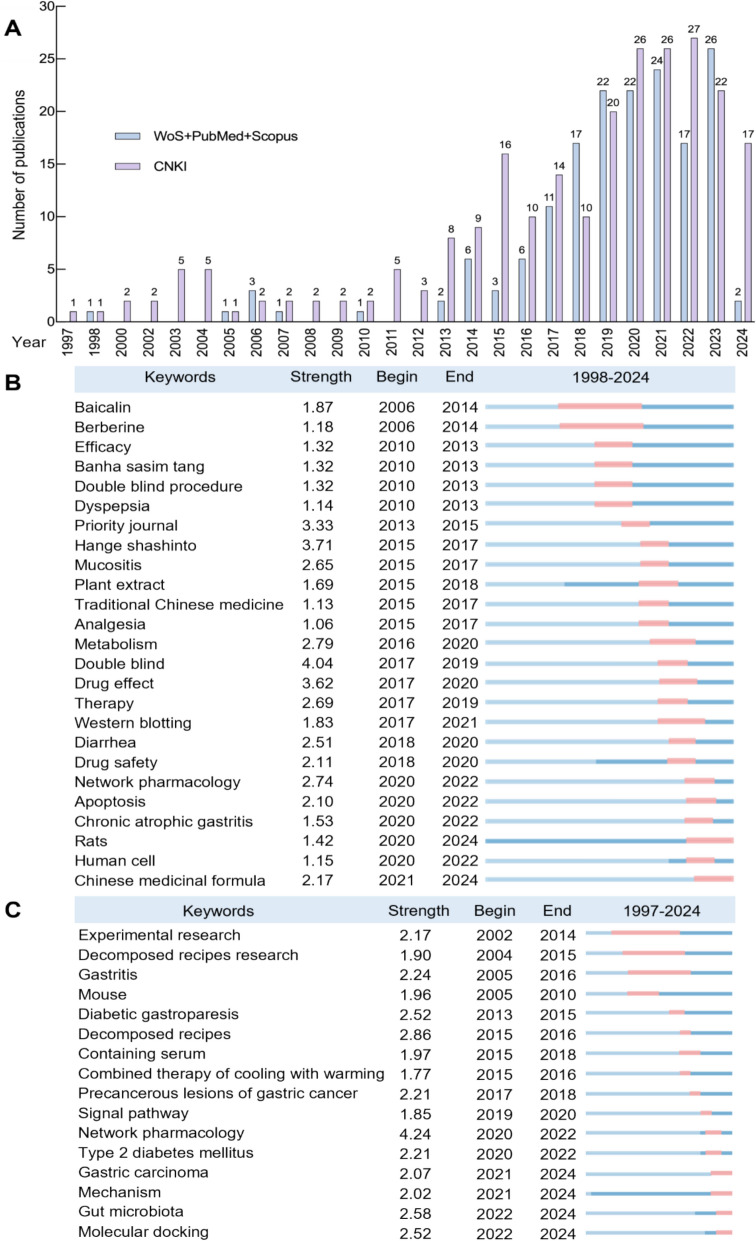
**A** Annual number of BXD-related publications retrieved from CNKI, WoS, PubMed, and Scopus; **B** keywords with the strongest citation burst among BXD-related publications indexed in WoS, PubMed, and Scopus; **C** keywords with the strongest citation burst among BXD-related publications indexed in CNKI

The chronological analysis of BXD-related publications reveals distinct patterns across international and Chinese databases. In international databases (WoS, PubMed, Scopus), the earliest study on BXD was published in 1998, followed by sporadic outputs until 2013, after which annual publications demonstrated sustained growth (Fig. 3A). Notably, temporary declines occurred in 2015 and 2022, but a significant surge began in 2014, with publication numbers tripling compared to the previous year. Subsequent growth rates remained robust, peaking at 333.3% in 2023. In contrast, the CNKI database documented an earlier inception of BXD research, with the first publication in 1997. However, outputs remained limited (35 cumulative articles) until 2013, after which Chinese scholars exhibited accelerated interest, producing 205 publications by 2024, including over 20 annual publications in the past 5 years. Cross-database comparisons highlight that Chinese researchers initiated BXD studies earlier (1997 vs. 1998), yet sustained growth in international databases lagged, commencing in 2017 compared to CNKI’s 2013 trajectory.

Keyword burst analysis underscores thematic shifts in BXD research. International databases exhibited early emphasis on phytochemical compounds such as “baicalin” and “berberine” (2006–2014), followed by a transition to clinical and methodological topics like “mucositis,” “double blind,” and “Western blotting” (2015–2017). Post-2020, emerging hotspots included “network pharmacology,” “apoptosis,” and “chronic atrophic gastritis,” while “rats” persisted as a consistent keyword since 1998 (Fig. 3B). In CNKI, long-term foci such as “experimental research,” “prescription decomposition,” and “gastritis” dominated for over a decade. Recent trends (2020–2024) shifted toward mechanistic explorations, including “gastric cancer,” “gut flora,” and “molecular docking” (Fig. 3C). Strikingly, only “network pharmacology” and “gastritis” overlapped as shared burst keywords between CNKI and international databases, reflecting divergent regional priorities.

It is obvious that CNKI demonstrates a significant advantage in publication volume (240 articles versus 165 international publications), but its lower keyword burst intensity indicates a narrower thematic focus, predominantly on pharmacological studies. This contrasts with the broader thematic diversity observed in global research. Meanwhile, distinct regional disparities in research priorities are apparent: Chinese scholarship emphasizes experimental and mechanistic investigations, as evidenced by high-frequency keywords such as “gut flora” and “molecular docking,” whereas international studies prioritize clinical methodologies (e.g., “double blind”) and cross-cultural applications. Moreover, non-Chinese regions exhibit a predominant focus on non-clinical BXD research, revealing a critical deficit in clinical validation efforts. Collectively, these findings underscore the imperative for a strategic integration of regional expertise to bridge methodological and translational gaps, thereby enhancing both mechanistic rigor and the clinical applicability of BXD research.

#### Country network and co-occurrence analysis

**Fig. 4:**
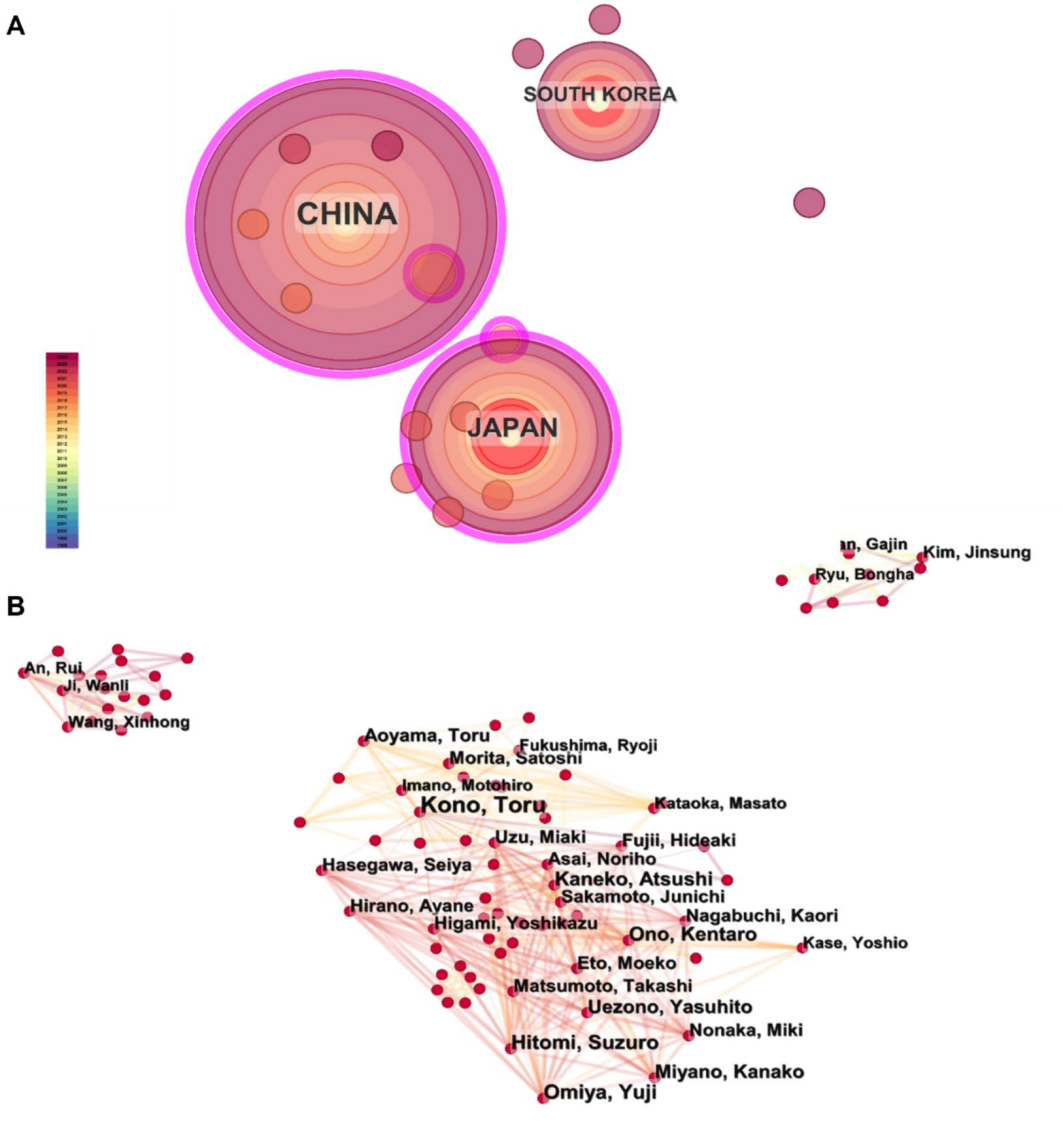
Country network and co-occurrence analysis of BXD studies in WoS, PubMed, and Scopus. **A** Global research landscape for BXD. **B** Author co-occurrence analysis of BXD

The country and institutional collaboration networks (Fig. 4A) reveal a fragmented global research landscape for BXD. China dominates with 95 screened publications (57.57% of total) from WoS, PubMed, and Scopus—exclusive of CNKI data. Japan and South Korea follow with 50 and 16 publications, respectively. Despite this output disparity, all three countries exhibit isolated nodes with minimal interconnections, reflecting limited international collaboration.

Author co-occurrence analysis (Fig. 4B) further highlights sparse domestic collaboration, particularly in China. Despite China’s publication dominance, institutional cooperation within the country remains underdeveloped: only three Chinese authors met the co-occurrence threshold (≥ 10 collaborations), compared to 24 in Japan. Lowering the threshold revealed more Chinese authors but underscored weaker collaborative networks relative to Japan.

Analysis of 165 screened publications identified three distinct author clusters corresponding to China, Japan, and South Korea. These clusters show negligible cross-border linkages, reinforcing the lack of global synergy. Notably, Japan’s robust intra-cluster collaboration contrasts sharply with China’s fragmented institutional networks, suggesting divergent research cultures in BXD studies.

#### Co-citation analysis

**Fig. 5:**
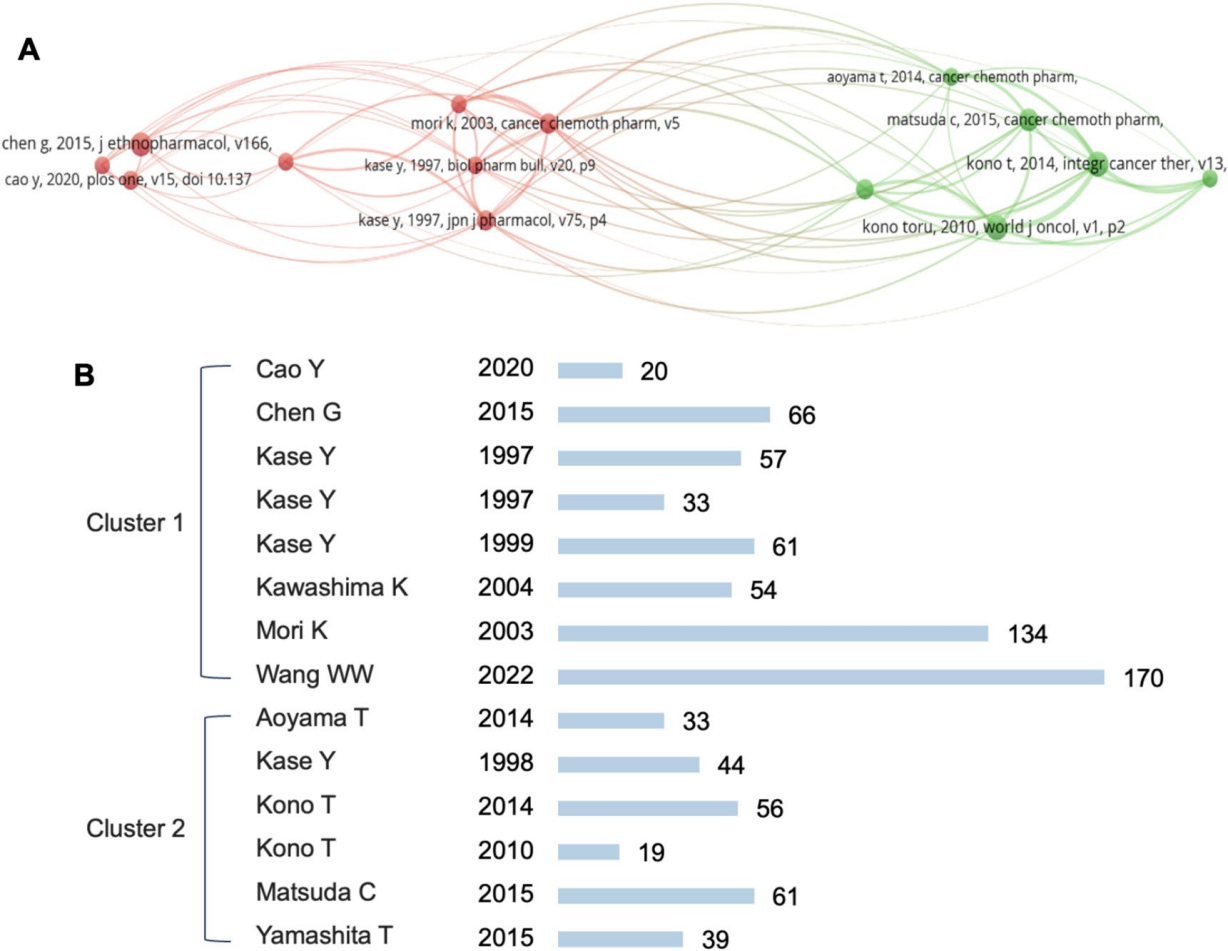
**A** Clusters of number of cited references. **B** Clusters of number of most cited authors

To map the intellectual foundations of BXD research, we performed a co-citation analysis of 3267 cited references. By applying a citation threshold of ≥ 14 citations per reference to ensure analytical precision, 14 highly cited references were identified and categorized into two thematic clusters (Fig. 5A).

The first cluster centers on BXD’s therapeutic applications for gastrointestinal diseases, particularly gastritis and chronic ulcerative colitis. A series of studies by Kase (1997–2004) established the foundational role of Hangeshashinto (TJ-14, a BXD formulation) in accelerating intestinal healing and mitigating chemotherapy-induced gastrointestinal toxicity [26–31]. These findings were further corroborated by Chen et al. who demonstrated BXD’s anti-inflammatory and antioxidant effects in alleviating experimental colitis [32]. Recent work by Cao et al. [33] reinforced BXD’s clinical superiority over combined traditional and Western therapies, while Wang et al. [34] expanded its potential by exploring the material basis of BXD’s efficacy in gastrointestinal disorders.

Cluster 2 highlights BXD’s clinical utility in managing oral mucositis. Early research by Kono revealed the effectiveness of topical TJ-14 application in chemotherapy-induced oral mucositis (COM) management [35], a discovery later refined through multicomponent analyses of its PGE2-regulating properties [36]. Clinical trials by Aoyama [37] and Matsuda et al. [38] provided robust evidence for TJ-14’s preventive effects in gastric and head/neck cancer patients, respectively, with Yamashita [39] further validating its capacity to reduce mucositis severity during chemoradiation.

Notably, the most frequently cited references—Matsuda et al. (2015, 61 citations) [38], Kono et al. (2014, 58 citations) [36], and Kase et al. (1998, 44 citations) [28]—span both clusters, reflecting their dual contributions to mechanistic and clinical research (Fig. 5B). Collectively, these findings illustrate a bifurcation in BXD scholarship: one strand advances mechanistic understanding of gastrointestinal therapies, while the other prioritizes clinical validation for oral mucositis. This dichotomy underscores the need for interdisciplinary collaboration to bridge preclinical insights with translational applications, ultimately enhancing BXD’s therapeutic potential.

## Function classification of BXD and pharmacological mechanism

**Fig. 6:**
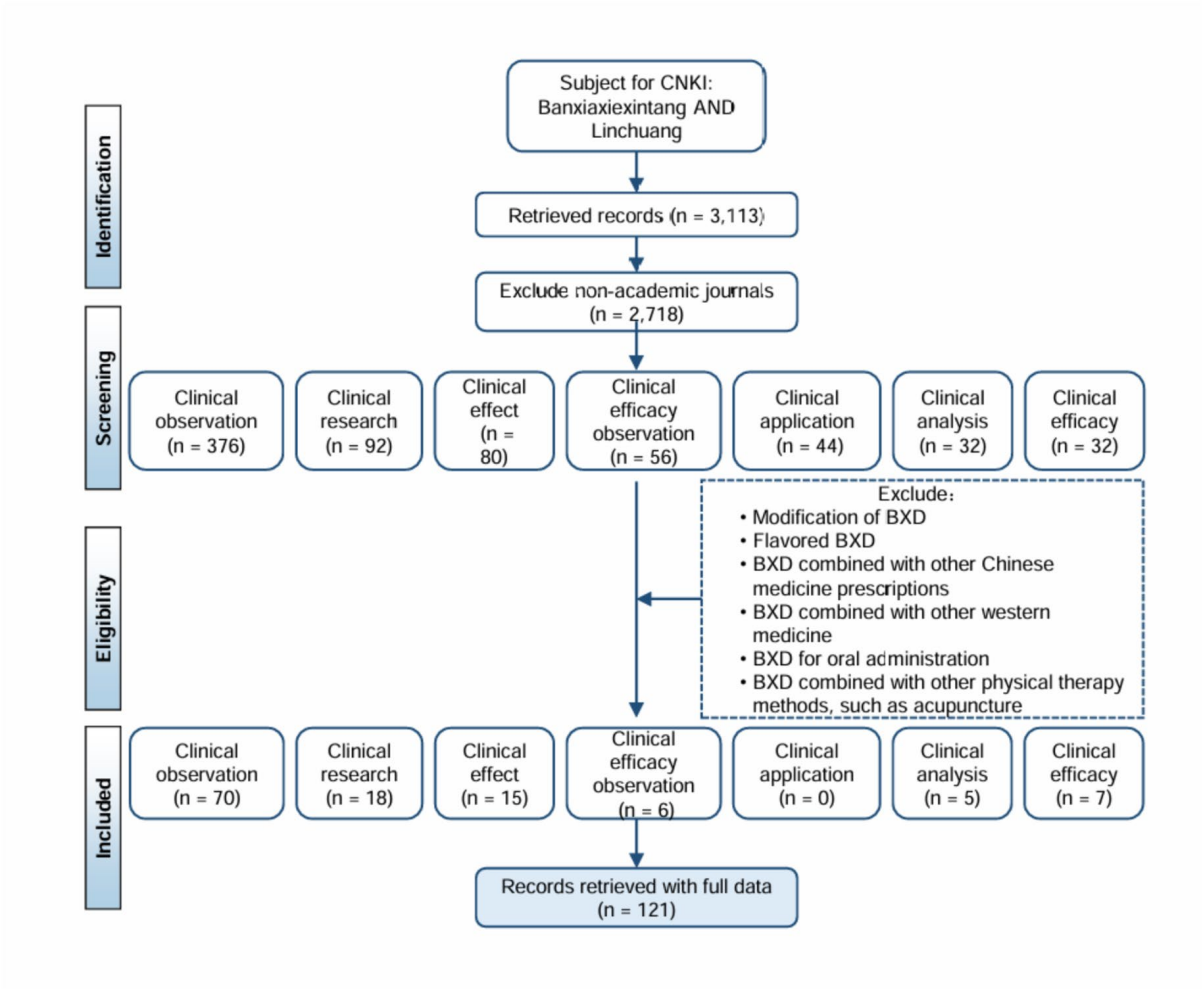
Screening process of the clinical application publications of BXD from CNKI

**Fig. 7:**
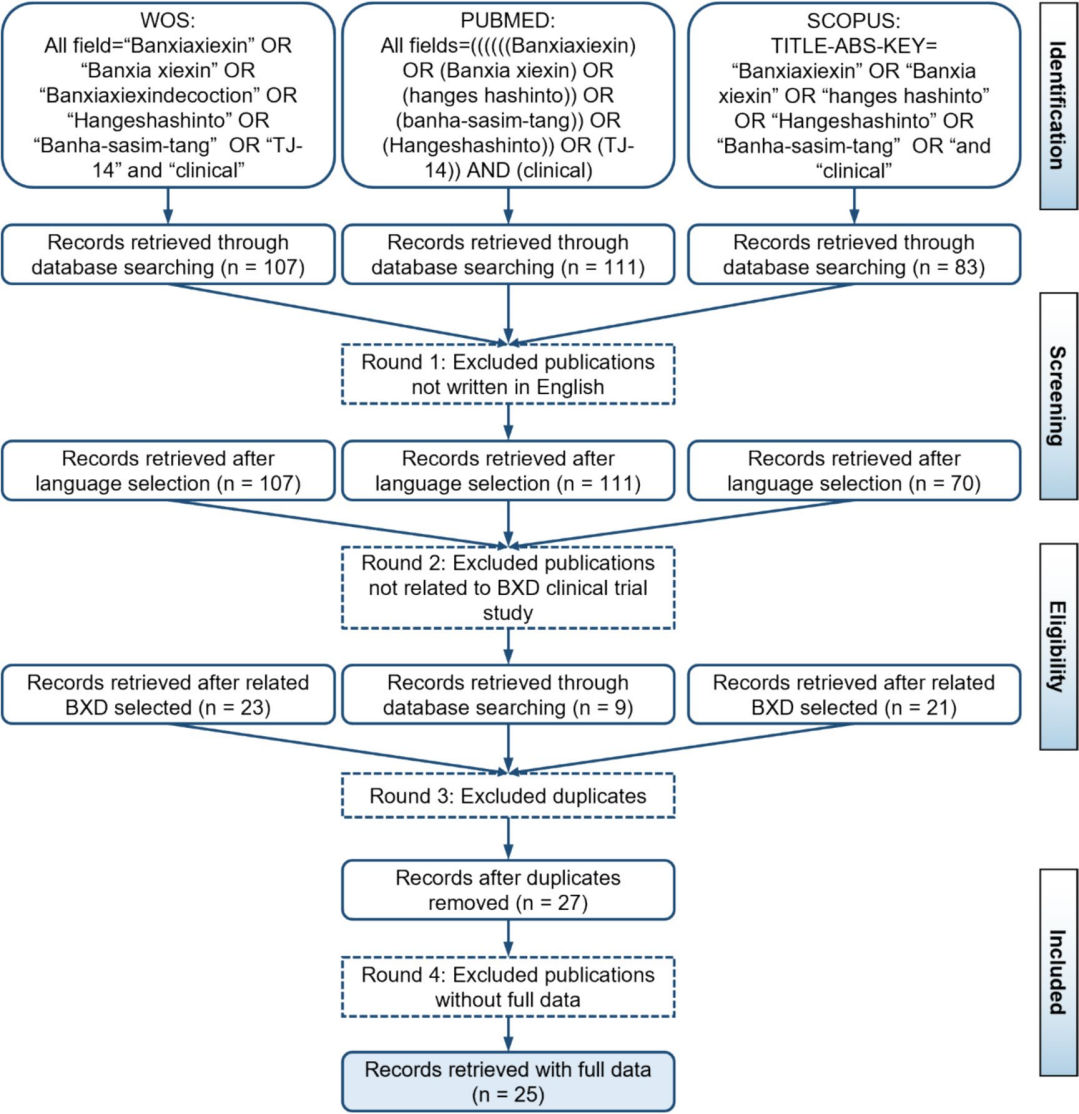
Screening process of the clinical application publications of BXD from WoS, PubMed and Scopus

To further focus on the analysis of the clinical efficacy of BXD, a systematic review of clinical studies was conducted. The search and screening process, executed in September 2024, adhered to predefined inclusion/exclusion criteria. Studies employing BXD as an adjuvant therapy or lacking explicit documentation of its primary clinical application were excluded. This yielded 121 eligible articles from CNKI (Fig. 6) and 25 articles from international databases (Web of Science, PubMed, Scopus) (Fig. 7). Data extraction followed a standardized protocol to ensure methodological consistency. Key parameters included study title, objectives, trial design, duration, sample size, patient demographics, functional classification, formulation specifications (prescription details), treatment protocols (administration route, dosage), and clinical outcomes (efficacy, adverse effects, and effect size). Discrepancies in data interpretation were resolved through iterative consensus—building discussions among the research team.

**Fig. 8:**
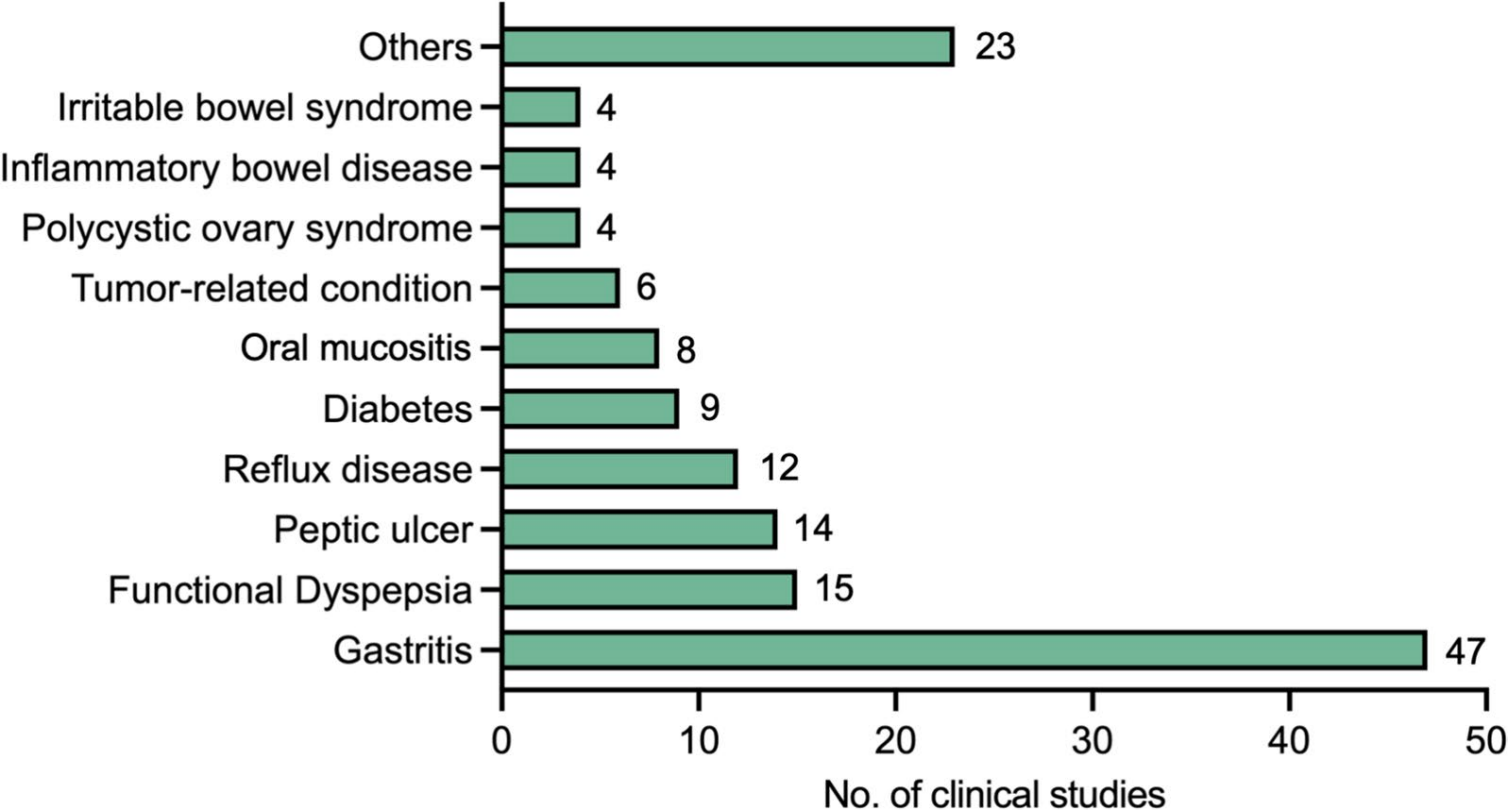
Classification of clinical application of BXD

All BXD—related clinical studies were categorized according to 11 clinical indications, including gastritis, functional dyspepsia, diabetes, reflux disease, tumor—associated conditions, and oral mucositis (Fig. 8). Evidently, digestive tract disorders such as gastritis represent the domains with the highest concentration of BXD clinical research. Subsequently, we conducted a more in-depth analysis of the efficacy and underlying mechanisms of BXD across these 11 clinical classifications.

### Gastritis

Gastritis, defined as inflammation of the gastric mucosa, arises from a variety of etiologic factors, including *Helicobacter pylori* infection, nonsteroidal anti-inflammatory drug (NSAID) use, alcohol consumption, chronic stress, and autoimmune disorders [40]. Among these, gastritis is the most common clinical indication for Banxia Xiexin Decoction (BXD), which has demonstrated therapeutic efficacy across multiple subtypes of this condition.

BXD demonstrates robust clinical efficacy in the staged, comprehensive treatment of refractory Helicobacter pylori infection (RHPI), thereby significantly enhancing patient compliance through targeted therapeutic action [41]. Mechanistic studies reveal BXD’s potent inhibitory effects on *H. pylori* proliferation, underpinning its high efficacy in treating *H. pylori*-positive gastritis [42–46]. Clinical trials further corroborate BXD’s anti-inflammatory properties, evidenced by its ability to markedly reduce serum levels of hs-CRP and IL-6 in patients with *H. pylori*-associated gastritis [47, 48].

BXD also exhibits multifaceted benefits in chronic atrophic gastritis (CAG) management (Supplementary Table 2). Clinical observations indicate that BXD alleviates core symptoms, mitigates gastric mucosal inflammation, and promotes mucosal repair through mechanisms including increased gastric gland proliferation and partial reversal of precancerous lesions [49–51]. Notably, BXD enhances biochemical markers of gastric function (e.g., G-17, PGI, PGII) and elevates *H. pylori* clearance rates, thereby reducing nausea, vomiting, and other symptomatic burdens [52–63].

Beyond pathogen-targeted therapy, BXD demonstrates broad utility in ameliorating chronic superficial gastritis (CSG), a condition characterized by plasma cell and lymphocyte infiltration of the gastric mucosa [64]. Randomized trials have confirmed that BXD effectively alleviated dyspeptic symptoms—such as acid reflux, epigastric pain, and belching—thereby warranting further large-scale clinical validation [65, 66]. However, while existing evidence underscores BXD’s therapeutic versatility, critical gaps persist. Current studies remain inconclusive regarding BXD’s capacity to mitigate adverse effects associated with sulfasalazine therapy [67], highlighting the need for mechanistic investigations into drug-herb interactions.

Emerging evidence elucidates the therapeutic mechanisms of BXD in managing drug-resistant *H. pylori*-associated gastritis (MIC = 256–512 μg/mL). BXD exerts multimodal action through immune activation, bactericidal enhancement, suppression of pathogen colonization, and attenuation of gastric mucosal inflammatory damage [68]. Additionally, preclinical studies in ethanol-induced gastritis models have shown that intragastric administration of BXD significantly ameliorates chronic gastric injury in rats, accompanied by reductions in pro-inflammatory mediators (TNF-α, IL-2, IL-8) and lactate dehydrogenase (LDH) levels [69].

The therapeutic potency of BXD is further underpinned by the synergistic activities of its component herbs. For example, extracts of Rhizoma Coptidis have been shown to modulate multiple inflammation-associated targets in *N*-methyl-*N*ʹ-nitro-*N*-nitrosoguanidine (MNNG)-induced CAG murine models [70], at the molecular level, bioactive compounds within BXD contribute to its anti-gastritis effects. Specifically, ginsenoside Rg1 alleviates CAG by suppressing pyroptosis via the NF-κB/NLRP3/GSDMD signaling pathway [71], and palmatine mitigates MNNG-induced CAG through STAT1/CXCL10 pathway regulation [72].

### Peptic ulcer

Peptic ulcer, an acid-peptic disorder, involves the erosion of the protective mucosal barrier in the stomach, proximal duodenum, or esophagus [73, 74]. BXD demonstrates significant therapeutic efficacy and favorable safety in the treatment of peptic ulcers [75–82]. BXD mitigates mucosal injury by reducing gastrin and motilin release, thereby alleviating local hypoperfusion and accelerating inflammatory resolution to promote ulcer healing [83]. Meanwhile, BXD can improve sleep quality in patients with peptic ulcers, addressing a common comorbidity of the condition [84].

Mechanically, BXD enhances gastric lymphatic pumping, alleviating the accumulation of inflammatory mediators and metabolic waste in the stomach, a critical mechanism in resolving stress-induced gastric ulceration [85]. BXD modulates the TGF-β/Smad signaling pathway by suppressing transforming growth factor-beta 1 (TGF-β1) and Smad3 expression while upregulating Smad7 in peptic ulcer patients [86]. Additionally, BXD inhibits excessive apoptosis of gastric mucosal epithelial cells by upregulating Bcl-2 mRNA expression and downregulating the apoptotic executor Caspase-3, thereby preserving mucosal integrity and mitigating injury [87]. BXD further reduces gastric inflammation by lowering levels of pro-inflammatory cytokines, including interleukin (IL)−2, IL-8, and tumor necrosis factor-alpha (TNF-α), in preclinical ulcer models [88].

### Functional dyspepsia (FD)

Functional dyspepsia (FD) is a chronic gastrointestinal disorder characterized by persistent dyspeptic symptoms in the absence of identifiable organic, systemic, or metabolic pathology [89]. BXD has demonstrated significant clinical efficacy in FD management. Specifically, it can elevate the fasting plasma motilin levels in patients, with an overall efficacy comparable to that of metoclopramide [90–95]. It may surpass conventional treatments in alleviating clinical symptoms such as epigastric pain and postprandial discomfort [96]. BXD is particularly effective in vulnerable populations, including the elderly and pediatric patients, where it ameliorates FD symptoms in individuals with compromised digestive function [97, 98]. Furthermore, BXD exhibits therapeutic benefits in non-ulcer dyspepsia (NUD), underscoring its broad applicability in functional gastrointestinal disorders [99].

Korean scholars have also expanded the evidence base for BXD (termed Banha-sasim-tang or BST) for the treatment of FD since 2010. A 2021 study by Sul-Ki Kim et al. identified plasma ghrelin modulation as a key mechanism underlying BST’s efficacy [100], with additional clinical trials corroborating its potential to improve FD symptoms [101–104].

Preclinical studies in FD rat models have elucidated BXD’s multifaceted mechanisms. BXD enhances gastric emptying and small intestinal propulsion rates, likely mediated by increased gastric mucin and substance P levels, alongside reduced gastrointestinal sensitivity [105, 106]. It also restores gut microbiota homeostasis by promoting probiotic proliferation, suppressing pathogenic bacteria, and preserving the colonic mucosal immune barrier post-microbiota disruption [107, 108]. Additionally, BXD modifies the ultrastructure of interstitial cells of Cajal (ICCs), critical regulators of gastrointestinal motility, further explaining its therapeutic impact on FD [109]. Recent research employing high-resolution mass spectrometer analysis in conjunction with multiple databases and molecular docking techniques, identified a total of 11 active compounds in BXD, especially berberine, could activate TAS2R38 and contributed to the amelioration of inflammation in the duodenum of FD mice [110]. Furthermore, certain constituent herbs or active compounds within BXD have also demonstrated therapeutic efficacy in the treatment of FD, with ginger serving as a notable example [111]. These findings collectively position BXD as a holistic intervention addressing both symptom relief and underlying pathophysiology in FD.

### Diabetes

Although BXD is extensively employed in glycemic management, it exhibits significant potential in attenuating diabetes-induced multi-organ damage. Clinical studies highlight its efficacy in addressing diabetic gastroparesis (DGP), a condition characterized by delayed gastric emptying. BXD alleviates DGP symptoms, including nausea, vomiting, abdominal distension, and early satiety, by enhancing gastric motility, shortening gastric emptying time, and modulating gastrointestinal hormone and inflammatory factor levels [112–116]. Furthermore, BXD improves metabolic parameters in diabetic non-alcoholic fatty liver disease (NAFLD), significantly reducing blood glucose, lipid levels, and hepatic dysfunction markers [117–119]. Comparative studies in type 2 diabetes management reveal that BXD, when integrated into treatment regimens, achieves superior clinical outcomes compared to conventional pharmacotherapy alone, underscoring its adjunctive therapeutic value [120].

Preclinical studies have also demonstrated the therapeutic potential and mechanism of BXD in addressing diabetes-related conditions. In diabetic mouse models, BXD significantly improved glucose and lipid metabolism while promoting insulin secretion [121]. In diabetic gastroparesis (DGP) models, BXD enhanced blood glucose regulation and accelerated gastric emptying. Mechanistically, these effects were attributed to the inhibition of advanced glycation end products (AGEs) production and receptor for AGE (RAGE) expression, coupled with the upregulation of neuronal nitric oxide synthase (nNOS). This modulation facilitated the proliferation of ICCs and ICC stem cells (ICC-SCs), thereby improving gastric motility [122]. In type 2 diabetes mellitus (T2DM) rat models, BXD regulated blood glucose homeostasis by altering serum bile acid metabolism profiles, upregulating farnesoid X receptor (FXR) expression, and modulating the secretion of serum and pancreatic glucagon-like peptide-1 (GLP-1) [123]. Additionally, BXD exhibited protective effects in pre-diabetes rats induced by 3-deoxyglucosone (3DG). Its mechanisms included reducing protein carbonylation levels, reactive oxygen species (ROS) levels, and inflammatory factor concentrations in the colon and serum [124]. Furthermore, BXD’s therapeutic efficacy in prediabetes was linked to its ability to modulate gut microbiota composition [125].

### Reflux disease

BXD demonstrates therapeutic efficacy in the management of several reflux-related disorders, including reflux esophagitis (RE), reflux gastritis, and gastroesophageal reflux disease (GERD). RE, a prevalent clinical gastrointestinal condition, remains incompletely understood in terms of pathogenesis [126, 127]. BXD alleviates RE symptoms such as epigastric pain, acid reflux, and gastric discomfort by promoting gastric emptying, reducing reflux stimulation, protecting the gastric mucosa, modulating gastric acid secretion, and enhancing gastrointestinal motility [128, 129]. Similarly, BXD exhibits clinical utility in GERD, a syndrome characterized by retrograde flow of gastrointestinal contents into the esophagus, manifesting as heartburn and acid regurgitation. Studies report that modified BXD formulations significantly improve GERD symptoms, including gastroesophageal reflux cough, with robust therapeutic outcomes [130–134]. Furthermore, BXD shows marked efficacy in treating reflux gastritis, underscoring its broad applicability in reflux disorders [135, 136]. Notably, complementary therapies such as Hangeshashinto may benefit patients with proton-pump inhibitor (PPI)-refractory GERD, particularly non-obese, non-elderly individuals with dyspepsia [137].

Mechanistic studies in a rat model of RE revealed that BXD reduces gastric acid secretion, regulates calcitonin gene-related peptide (CGRP) synthesis [138], protects esophageal mucosal integrity, and modulates neurotensin synthesis and secretion [139]. These findings highlight BXD’s multifaceted mechanisms in mitigating reflux pathology, combining mucosal protection, neuromodulation, and acid suppression to restore gastrointestinal homeostasis. Meanwhile, the active compounds present in BXD have also demonstrated notable bioactivity in the context of reflux disease. For instance, berberine has been shown to inhibit the production of pro-inflammatory cytokines in a rat model of GERD [140].

### Inflammatory bowel disease (IBD)

Inflammatory bowel disease (IBD) is a general term for chronic or remitting/relapsing inflammatory diseases of the intestinal tract and generally refers to ulcerative colitis (UC) and Crohn’s disease (CD) [141]. Due to the complex etiology of inflammatory bowel disease (IBD) and the limitations of current treatment options, Banxia Xiexin Decoction (BXD) has demonstrated unique advantages in the management of IBD [142, 143]. A meta-analysis incorporating 10 randomized controlled trials (RCTs) involving 768 patients revealed that BXD exhibited significantly higher efficacy (P < 0.00001) and reduced serum TNF-α levels (P = 0.001) compared to control groups. However, no significant difference was observed in the incidence of adverse reactions (P = 0.23) [144]. Clinical observations have indicated that the therapeutic efficacy of BXD in the treatment of UC is, at least in part, contingent upon its regulatory influence on the intestinal microbiota of patients [145].

Numerous studies have elucidated the mechanisms by which BXD exerts its therapeutic effects on UC, highlighting its multifaceted actions, including immune regulation, anti-inflammatory activity, intestinal barrier repair, oxidative stress reduction, and tissue repair through stem cell mobilization. BXD has been shown to promote ulcer healing by modulating immune cell balance. In dextran sulfate sodium (DSS)-induced UC mouse models, BXD restored the equilibrium between Th17/Treg [146] and Th1/Th2 immune cells [147], thereby suppressing excessive immune responses. Additionally, BXD exerts anti-inflammatory effects by regulating the levels of pro-inflammatory factors and anti-inflammatory factors, modulating the relevant signaling pathways, and attenuating the inflammatory damage [148–152]. In trinitrobenzene sulfonic acid (TNBS)-induced UC mouse models, BXD elevated the expression of ZO-1 and occludin in the colon, reduced intestinal mucosal permeability, and decreased d-lactic acid levels, indicating its role in maintaining colonic mucosal tight junction integrity and repairing the damaged intestinal mucosal barrier [153]. Furthermore, BXD attenuates oxidative stress by restoring superoxide dismutase (SOD) activity and intervening in the Nrf2 signaling pathway in DSS-induced UC mouse models [32]. Furthermore, BXD facilitated the homing of mesenchymal stem cells (MSCs) to the colonic mucosa, promoting tissue repair and improving the histopathological condition of the colon in TNBS-induced UC rat models [154].

### Irritable bowel syndrome (IBS)

Irritable bowel syndrome (IBS) is a functional gastrointestinal disorder characterized by abdominal pain or discomfort, accompanied by alterations in bowel habits. Currently, effective treatment options for IBS remain limited [155]. Several clinical studies have demonstrated the potential of BXD in alleviating abdominal pain, discomfort, and diarrhea associated with IBS [156–159]. In a prospective, randomized, controlled trial, although a 4-week treatment with BXD did not show a statistically significant difference compared to the control group in patients with diarrhea-predominant IBS (D-IBS), it significantly modulated ghrelin secretion, which may contribute to improved gastrointestinal function [156]. Another study reported that a modified BXD formulation achieved an overall effectiveness rate of 93.3% in treating D-IBS, significantly higher than the 75.0% observed in the control group [157].

Through a systems pharmacology approach, researchers identified 36 active compounds in BXD, including wogonin, glycyrrhetinic acid, berberine, and ginsenoside Rh4. The study revealed that the cross-talk interactions among the TNF signaling pathway, dopaminergic synapse, and cGMP-PKG signaling pathway may synergistically influence the pathogenesis and treatment of IBS [160]. Additionally, numerous studies have highlighted the therapeutic potential of individual active compounds derived from BXD for IBS management, such as berberine [161], Coptisine [162], and 6-Shogaol [163]. These findings underscore the multifaceted mechanisms through which BXD and its components may exert beneficial effects in IBS treatment.

### Oral mucositis

Oral mucositis is a frequent and clinically significant side effect in cancer patients undergoing chemotherapy or radiation therapy. Japanese researchers have widely investigated the therapeutic effect of Hangeshashinto (TJ-14) on COM. Among the seven selected clinical trials evaluating the treatment of oral mucositis, all relevant studies originated Japan. The earliest clinical study, conducted in 2013, involved a double-blind trial of 91 patients who received either Hangeshashinto or placebo for 2 to 6 weeks [37]. The results indicated that while Hangeshashinto treatment did not significantly reduce the incidence of ≥ 2 COM in patients developing mucositis during gastric cancer chemotherapy, it tends to lower the risk of COM in patients with grade 1 COM during the screening cycle. The study pointed out that follow-up phase III studies with larger sample sizes are necessary to elucidate the protective effect of Hangeshashinto on COM. A 2014 study involving 80 patients demonstrated that Hangeshashinto was effective in ameliorating oral mucositis induced by radiation therapy in patients with head and neck cancer. Hangeshashinto was associated with improved completion rates of cisplatin-based chemoradiotherapy [39]. Other studies have shown that, although the incidence of grade ≥ 2 oral mucositis was lower in patients treated with Hangeshashinto compared to those receiving a placebo, the difference was not statistically significant [38, 164, 165]. However, Hangeshashinto showed significant efficacy compared with placebo in the treatment of grade ≥ 2 mucositis in patients with colorectal cancer. Studies also suggest that Hangeshashinto cannot prevent the occurrence of COM [166], but it can reduce its severity [167]. Another preliminary study showed that the combination of Hangeshashinto and cryotherapy may be effective in treating radiation induced oral stomatitis [168].

Despite the widespread reporting of Hangeshashinto’s effectiveness in managing oral mucositis, the underlying mechanisms of action remain to be fully elucidated. Some studies explored the ingredient-specific pharmacological actions of Hangeshashinto. For instance, 6-gingerol and 6-shogaol, two bioactive compounds derived from Hangeshashinto, have been shown to alleviate pain associated with oral ulcerative mucositis through modulation of Na^+^ channels [169]. A randomized clinical trial demonstrated that a licorice-based mucoadhesive film, containing herb of Hangeshashinto, significantly reduced pain severity and mitigated the progression of radiation-induced mucositis [170].

### Tumor-related conditions

BXD has demonstrated significant potential as a widely applicable adjuvant therapy in cancer treatment. Clinically, it effectively addresses gastric precancerous lesions and liver-spleen disharmony following colorectal cancer surgery. Studies have shown that BXD reduces serum carcinoembryonic antigen (CEA) levels, accelerates postoperative recovery, and alleviates surgical complications [171, 172]. Additionally, Hangeshashinto has been shown to reduce the risk of afatinib-induced diarrhea, a common adverse effect in cancer therapy [173], and effectively mitigates chemotherapy-related gastrointestinal symptoms, such as nausea and vomiting, in patients with digestive tract tumors [174]. Furthermore, BXD exhibits broad-spectrum anti-tumor activity, showing therapeutic benefits in colon cancer and hepatocellular carcinoma (HCC) [175, 176]. When combined with chemotherapy, modified BXD formulations not only alleviate chemotherapy-induced clinical symptoms but also significantly prolong disease-free survival (DFS) in patients with stage III colon cancer [177], gastric cancer (GC) [178], and duodenal cancer [179].

BXD exhibits multifaceted anti-tumor effects across various cancer models, including inhibition of tumor growth, suppression of invasion, delay of cancer progression, and modulation of drug sensitivity through the regulation of multiple cancer-related signaling pathways. In GC models, BXD significantly inhibits tumor growth and delays disease progression, potentially mediated by the regulation of the PI3K–Akt signaling pathway [180]. Similarly, in colitis-associated cancer (CAC) models, BXD impedes the colitis-to-cancer transition by suppressing the E-cadherin/β-catenin pathway, particularly through inhibition of Fusobacterium nucleatum FadA activity [181]. BXD also enhances drug sensitivity in gastric cancer cells by regulating MGMT expression through the IL-6/JAK/STAT3-mediated PD-L1 signaling axis [182]. This mechanism underscores BXD’s potential to overcome chemoresistance and improve therapeutic outcomes.

### Polycystic ovary syndrome (PCOS)

Polycystic ovary syndrome (PCOS) is a prevalent endocrine and metabolic disorder affecting women of reproductive age, characterized by oligo-anovulation, hyperandrogenism, polycystic ovarian morphology, and metabolic dysregulation, including abdominal obesity and insulin resistance (IR) [183]. Emerging clinical evidence suggests that modified BXD demonstrates therapeutic efficacy in ameliorating insulin sensitivity and restoring reproductive endocrine balance in patients with PCOS-associated IR (PCOS-IR). These improvements correlate with enhanced clinical outcomes, such as restoration of spontaneous menstruation, elevated pregnancy rates, and metabolic normalization, potentially mediated through IR correction and endocrine stabilization [184]. In PCOS patients with hyperinsulinemia (PCOS-HI) presenting with traditional Chinese medicine (TCM) syndromes of stomach heat and spleen deficiency, BXD exhibits comparable efficacy to metformin in reducing fasting insulin (FINS) levels and enhancing insulin sensitivity. Notably, modified BXD surpasses metformin in restoring spontaneous ovulation rates and alleviating oligomenorrhea, effects attributed to its capacity to modulate FINS levels [185, 186].

Mechanistic studies indicate that BXD may restore sex hormone equilibrium in PCOS by modulating gut microbiota composition, including enrichment of beneficial taxa, suppression of pathogenic species, and reestablishment of intestinal microecological homeostasis [187]. Preclinical studies utilizing a letrozole and high-fat diet-induced PCOS-IR rat model further demonstrate that modified BXD formulations attenuate IR and metabolic dysfunction via gut microbiota regulation [188, 189]. Additionally, modified BXD improves IR and endocrine parameters in PCOS-IR rats, potentially through activation of the PI3K/Akt/GLUT4 signaling pathway [190]. Another recent study highlights BXD’s role in suppressing ovarian granulosa cell proliferation and inflammation in PCOS models by upregulating miR-20b-5p and inhibiting the TLR4/IL-6 axis [191].

Meanwhile, emerging evidence highlights the pharmacological efficacy of bioactive constituents in BXD PCOS intervention. Notably, berberine, a key alkaloid of BXD, ameliorates PCOS-associated inflammation through downregulation of hyaluronan synthase 2 (HAS2) [192], while concurrently enhancing ovulation rates and endometrial receptivity [193]. Similarly, 6-gingerol, a primary bioactive phytochemical in ginger, exhibits therapeutic promise in PCOS models. In estradiol valerate-induced PCOS rats, 6-gingerol attenuates pathological increases in body weight, ovarian hypertrophy, and sex steroid dysregulation. These effects correlate with suppression of cyclooxygenase-2 (COX-2) expression, suggesting a mechanism involving modulation of inflammatory pathways [194].

### Other conditions

In addition to its well-documented efficacy in the 10 primary disease classifications, BXD has demonstrated therapeutic potential across a broad spectrum of clinical conditions. These include diarrhea, ventricular premature beats, sleep disorders, chronic gastric issues, respiratory-related pneumonia, chronic subjective dizziness, impaired fasting blood sugar, and chronic cough.

BXD has been shown to prevent and control irinotecan (CPT-11)-induced delayed diarrhea, with its efficacy validated in clinical studies [195]. Additionally, Hangeshashinto is effective in managing acute radiation-induced enteritis (ARE) [196]. BXD could also alleviate postprandial distress syndrome (PDS) [197] and reduces the incidence and severity of postoperative sore throat in patients undergoing laparoscopic surgery [198]. Furthermore, given the role of *H. pylori* in halitosis, BXD has shown significant efficacy in treating *H. pylori*-related halitosis [199].

**Fig. 9:**
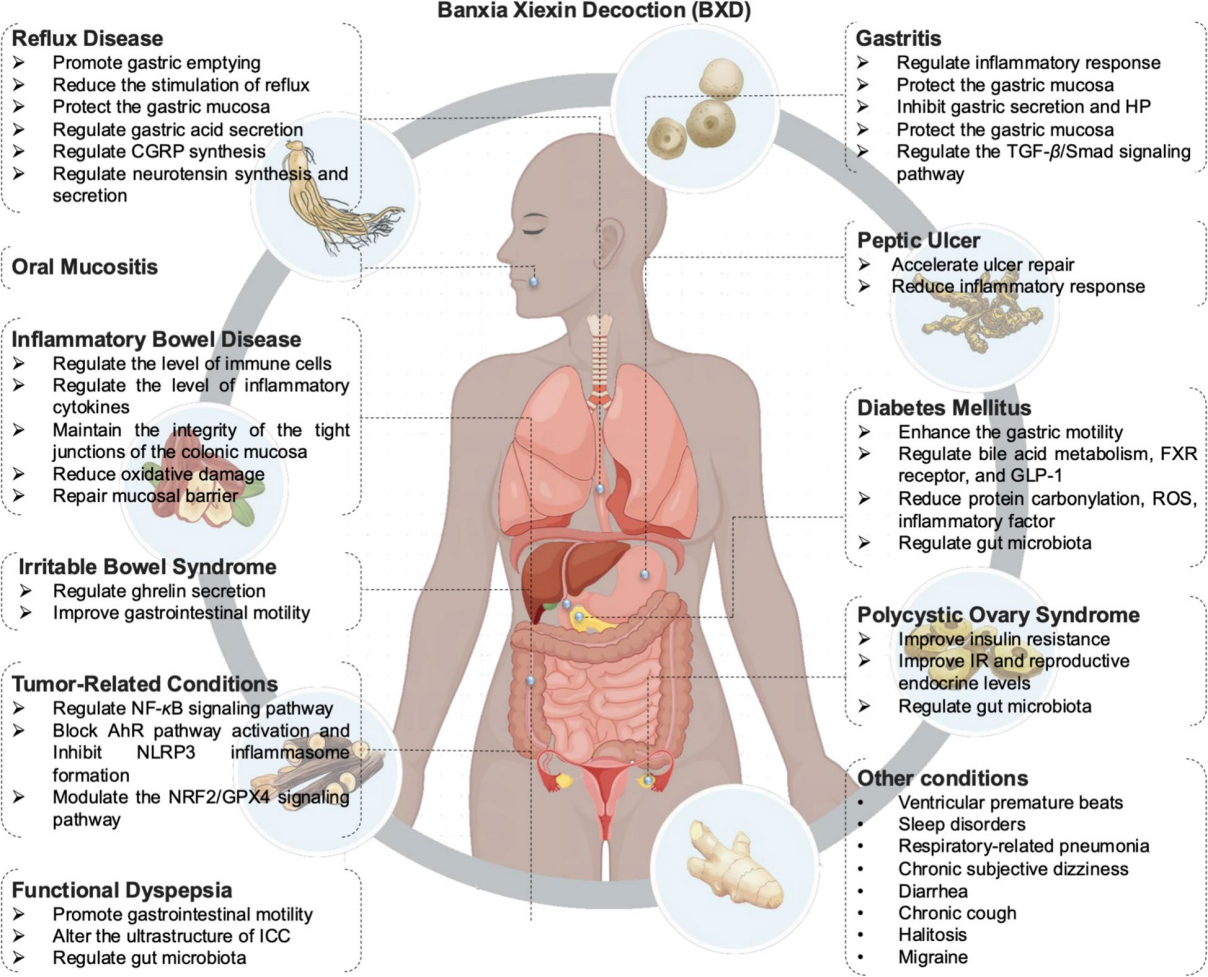
Clinical application of BXD and related mechanism

Preclinical studies suggest that BXD may have therapeutic potential for migraine, as it reduces plasma levels of neuropeptides [e.g., calcitonin gene-related peptide (CGRP) and substance P (SP)], increases endothelin (ET) levels, and downregulates early-response genes (e.g., C-FOS and C-JUN) in experimental migraine models [200]. These findings provide a promising research direction for BXD’s clinical application in migraine management (Fig. 9).

## Discussion and perspectives

Since September 2020, the National Medical Products Administration of China has implemented new registration categories for TCM, categorizing classical prescription preparations as one of the four types of new drugs. This initiative has significantly spurred the development of classical prescriptions. To date, over a dozen classical prescription preparations, such as Linggui Zhugan Decoction, have received approval for marketing. However, since most classical prescriptions are formulated to address specific TCM syndromes, their expression in TCM terminology may encounter substantial challenges in clinical application and market promotion, hindering the full realization of their clinical value. Consequently, clarifying clinical indications and accurately reflecting applicable diseases in drug labeling are critical and unavoidable issues for classical prescription products.

Over the past 25 years, the enthusiasm for the research and development of BXD has continued to grow, and literature reviews have provided valuable insights into evolving research trends. China, Japan, and South Korea are the leading countries in advancing BXD research. Notably, while Japanese scholars initially published a higher number of papers, by 2018, the number of publications from China had surpassed that of Japan, positioning China as the most prolific contributor in this field. The most productive research institutions are primarily located in Japan and China. However, the citation counts for Chinese researchers still lag behind those of their Japanese counterparts, suggesting that the quality and impact of their research need further enhancement. We propose that with the accelerated development of BXD R&D and increased international and institutional collaborations, the research network will become increasingly complex and interconnected. However, we believe that the development of BXD should still prioritize the following aspects.

The stability and reliability of quality are fundamental prerequisites for the clinical efficacy of classical prescription preparations. Ensuring every detail, including the quality of herbs, decoction pieces, and manufacturing processes, is essential for maintaining the stability and reliability of these preparations. This meticulous attention to detail is both critical and an inevitable requirement for ensuring their quality. For example, He’s study emphasized the impact of decoction methods on the chemical composition and pharmacological activity of BXD [201]. Simultaneously, the clear delineation of critical quality attributes is indispensable for attaining cohesive control across various stages, thus guaranteeing comprehensive quality control throughout the entire process. However, given the complex composition of BXD, the quality control marker for Pinelliae Rhizoma and jujube remain undefined. Consequently, advanced analytical techniques are required to perform in-depth characterization of their constituents and establish correlations with relevant biological activities, thereby identifying critical quality attributes. It is imperative to underscore that the enhancement of quality is inherently linked to increased manufacturing expenses, which further demands that the product possess significant clinical and market value [202].

In the context of clinical research, significant challenges arise from inconsistencies in BXD dosing and administration protocols, as well as the variable quality of studies, which hinder the comprehensive analysis and synthesis of BXD’s clinical benefits. For example, as per the key information of BXD issued by the National Medical Products Administration and the National Administration of Traditional Chinese Medicine of China, BXD comprises seven herbal ingredients, with the cumulative dosage amounting to 83.3 g. Conversely, within the Japanese Pharmacopoeia, there are three distinct prescriptions for BXD (Hangeshashinto). These are categorized into versions containing either processed ginger or ginger, and the dosages specified therein are significantly lower than those in the officially published Chinese version, which range from 18.5 to 22 g. Moreover, in the Japanese Pharmacopoeia, both Pinelliae Rhizoma and Glycyrrhizae Radix et Rhizoma are presented in their unprocessed forms. The disparities in prescriptions and dosages pose challenges in directly comparing and evaluating the clinical efficacy of BXD. Consequently, future research endeavors should focus on conducting systematic investigations into the dose–response relationships of BXD preparations for various diseases. This approach will facilitate a more comprehensive understanding of the optimal utilization of BXD in clinical practice.

BXD has exhibited promising therapeutic efficacy in the management of various gastrointestinal disorders, including gastritis, IBD, and FD. Our findings indicate that the majority of clinical investigations on BXD have predominantly centered on these conditions. Simultaneously, recent studies have revealed that BXD demonstrates unique therapeutic potential in other clinical domains. For instance, since 2020, research exploring the application of BXD in PCOS and peptic ulcers has emerged, marking a notable shift as prior to this period, BXD was scarcely utilized in these areas. Furthermore, BXD has been shown to possess distinct therapeutic properties in the treatment of oral mucositis, as evidenced by multiple clinical studies. However, current research on BXD in this context has largely focused on mucositis induced by chemotherapy or radiotherapy, with conventional forms of mucositis remaining underexplored. We propose that this represents a promising avenue for future research and development.

Simultaneously, given that BXD is frequently employed in the management of chronic gastrointestinal disorders, including chronic gastritis and IBD, and considering that Pinelliae Rhizoma, an ingredient in BXD, exhibits mild toxicity, the safety profile of BXD emerges as a critical aspect that demands due attention. Generally speaking, BXD is widely recognized for its safety, and clinical studies have not reported any substantial adverse effects. Nevertheless, in the absence of a comprehensive and systematic assessment of its toxicity, long-term and indiscriminate use of BXD is not advisable. In subsequent research, it is imperative to conduct high-quality randomized controlled clinical trials following rigorous scientific protocols. These trials will play an indispensable role in generating more reliable and conclusive evidence to support the clinical application of BXD.

## Supplementary Information

Supplementary Material 1

## Data Availability

The study’s original data can be obtained from the corresponding authors upon reasonable request.

## References

[1] Zhang C, Liu Y, Li H, Li B, Dong Y, Liu S. Analysis on evolution of clinical application of classic famous prescription Banxia Xiexin Decoction in ancient and modern times. J Basic Chin Med. 2023;29(03):452–6.

[2] Miyano K, Hasegawa S, Asai N, Uzu M, Yatsuoka W, Ueno T, et al. The Japanese herbal medicine Hangeshashinto induces oral keratinocyte migration by mediating the expression of CXCL12 through the activation of extracellular signal-regulated kinase. Front Pharmacol. 2021;12: 695039. 35145397 10.3389/fphar.2021.695039 PMC8822321

[3] Pharmacopoeia Commission of the People’s Republic of China. The Pharmacopoeia of the People’s Republic of China. Beijing: China Medical Science Press; 2020.

[4] Bai J, Qi J, Yang L, Wang Z, Wang R, Shi Y. A comprehensive review on ethnopharmacological, phytochemical, pharmacological and toxicological evaluation, and quality control of *Pinellia**ternata* (Thunb.) Breit. J Ethnopharmacol. 2022;298: 115650. 35988838 10.1016/j.jep.2022.115650

[5] Chen C, Sun Y, Wang Z, Huang Z, Zou Y, Yang F. *Pinellia* genus: a systematic review of active ingredients, pharmacological effects and action mechanism, toxicological evaluation, and multi-omics application. Gene. 2023;870: 147426. 37044184 10.1016/j.gene.2023.147426

[6] Wang J, Wang L, Lou G-H, Zeng H-R, Hu J, Huang Q-W, et al. Coptidis Rhizoma: a comprehensive review of its traditional uses, botany, phytochemistry, pharmacology and toxicology. Pharm Biol. 2019;57(1):193–225. 30963783 10.1080/13880209.2019.1577466 PMC6461078

[7] Ma W, Liu T, Ogaji OD, Li J, Du K, Chang Y. Recent advances in Scutellariae radix: a comprehensive review on ethnobotanical uses, processing, phytochemistry, pharmacological effects, quality control and influence factors of biosynthesis. Heliyon. 2024;10(16): e36146. 39262990 10.1016/j.heliyon.2024.e36146 PMC11388511

[8] Chen L, Wang H, Chen Z, Zhuo W, Xu R, Zeng X. The effect of dried ginger (Gan Jiang) on stomach energy metabolism and the related mechanism in rats based on metabonomics. Chem Biodivers. 2022;19(11): e202200757. 36226702 10.1002/cbdv.202200757

[9] Song W, Qiao X, Chen K, Wang Y, Ji S, Feng J. Biosynthesis-based quantitative analysis of 151 secondary metabolites of licorice to differentiate medicinal *Glycyrrhiza* species and their hybrids. Anal Chem. 2017;89(5):3146–53. 28192986 10.1021/acs.analchem.6b04919

[10] Zhang Z, Li J, Li F, Wang T, Luo X, Li B. Jujubae fructus extract prolongs lifespan and improves stress tolerance in *Caenorhabditis**elegans* dependent on DAF-16/SOD-3. Sci Rep. 2024;14(1):13713. 38877105 10.1038/s41598-024-64045-0 PMC11178930

[11] Hou J-P. The chemical constituents of ginseng plants. Am J Chin Med. 1977;05(02):123–45. 10.1142/s0147291777000209 608333

[12] Zhao B, Lv C, Lu J. Natural occurring polysaccharides from *Panax**ginseng* C. A. Meyer: a review of isolation, structures, and bioactivities. Int J Biol Macromol. 2019;133:324–36. 30943421 10.1016/j.ijbiomac.2019.03.229

[13] Wang Y, Xu R, Xiao J, Zhang J, Wang X, An R. Quantitative analysis of flavonoids, alkaloids and saponins of Banxia Xiexin Decoction using ultra-high performance liquid chromatography coupled with electrospray ionization tandem mass spectrometry. J Pharm Biomed Anal. 2014;88:525–35. 24189040 10.1016/j.jpba.2013.10.002

[14] Committee T J P E. The Japanese pharmacopoeia. Japanese Ministry of Health, Labour and Welfare Press; 2021.

[15] Yan L, Shi J, Wang J, Shi Y. UPLC/Q-TOF-MSE based analysis of chemical composition of Banxia Xiexin Decoction. Acta Pharmaceutica Sinica. 2013;48(04):526–31. 23833940

[16] Wang Y C, Qiu Z D, Wang L Y, Wang M X and Dong X L, Quality analysis of Banxia Xiexin Decoction material benchmark by high performance liquid chromatography fingerprint with chemometrics. Phys Test Chem Anal Part B Chem Anal 2024. 1–8.

[17] Sun B, Zhao YF, Zhu GW, Zhang D, Qu YZ, Chang AQ. Application of DESI-MSI in quality control of Banxia Xiexintang. Chin J Exp Tradit Med Formulae. 2020;26(07):117–28.

[18] Pritchard A. Statistical bibliography or bibliometrics? J Doc. 1969;25(4):348–9.

[19] Meng H, Liu X, Li J, Bao T, Yi F. Bibliometric analysis of the effects of ginseng on skin. J Cosmet Dermatol. 2022;21(1):99–107. 34520601 10.1111/jocd.14450

[20] Chen C. Searching for intellectual turning points: progressive knowledge domain visualization. Proc Natl Acad Sci USA. 2004;101(1):5303–10. 14724295 10.1073/pnas.0307513100 PMC387312

[21] Baker DR. Citation analysis: a methodological review. Soc Work Res Abstracts. 1990;26(3):3–10.

[22] Van Eck N, Waltman L. Software survey: VOSviewer, a computer program for bibliometric mapping. Scientometrics. 2010;84(2):523–38. 20585380 10.1007/s11192-009-0146-3 PMC2883932

[23] Xu H, Lin X, Shi K, Lin S, Zheng G, Wang Q. Research progress and hotspot evolution analysis of landscape microclimate: visual analysis based on CNKI and WOS. Int J Environ Res Public Health. 2022;19(22):15118. 36429831 10.3390/ijerph192215118 PMC9691154

[24] Trujillo CM, Long TM. Document co-citation analysis to enhance transdisciplinary research. Sci Adv. 2018;4(1): e1701130. 29308433 10.1126/sciadv.1701130 PMC5752411

[25] Van Eck N, Waltman L. Citation-based clustering of publications using CitNetExplorer and VOSviewer. Scientometrics. 2017;111(2):1053–70. 28490825 10.1007/s11192-017-2300-7 PMC5400793

[26] Kase Y, Hayakawa T, Aburada M, Komatsu Y, Kamataki T. Preventive effects of Hange-shashin-to on irinotecan hydrochloride-caused diarrhea and its relevance to the colonic prostaglandin E2 and water absorption in the rat. Jpn J Pharmacol. 1997;75(4):407–13. 9469647 10.1254/jjp.75.407

[27] Mori K, Kondo T, Kamiyama Y, Kano Y, Tominaga K. Preventive effect of Kampo medicine (Hangeshashin-to) against irinotecan-induced diarrhea in advanced non-small-cell lung cancer. Cancer Chemother Pharmacol. 2003;51(5):403–6. 12687289 10.1007/s00280-003-0585-0

[28] Kase Y, Hayakawa T, Ishige A, Aburada M, Komatsu Y. The effects of Hange-shashin-to on the content of prostaglandin E2 and water absorption in the large intestine of rats. Biol Pharm Bull. 1997;20(9):954–7. 9331975 10.1248/bpb.20.954

[29] Kase Y, Saitoh K, Makino B, Hashimoto K, Ishige A, Komatsu Y. Relationship between the antidiarrhoeal effects of Hange-Shashin-To and its active components. Phytother Res. 1999;13(6):468–73. 10479755 10.1002/(sici)1099-1573(199909)13:6<468::aid-ptr504>3.0.co;2-v

[30] Kase Y, Saitoh K, Ishige A, Komatsu Y. Mechanisms by which Hange-shashin-to reduces prostaglandin E2 levels. Biol Pharm Bull. 1998;21(12):1277–81. 9881638 10.1248/bpb.21.1277

[31] Kawashima K, Nomura A, Makino T, Saito K, Kano Y. Pharmacological properties of traditional medicine (XXIX): effect of hange-shashin-to and the combinations of its herbal constituents on rat experimental colitis. Biol Pharm Bull. 2004;27(10):1599–603. 15467203 10.1248/bpb.27.1599

[32] Chen G, Yang Y, Liu M, Teng Z, Ye J, Xu Y. Banxia Xiexin Decoction protects against dextran sulfate sodium-induced chronic ulcerative colitis in mice. J Ethnopharmacol. 2015;166:149–56. 25794808 10.1016/j.jep.2015.03.027

[33] Cao Y, Zheng Y, Niu J, Zhu C, Yang D, Rong F, et al. Efficacy of Banxia Xiexin Decoction for chronic atrophic gastritis: a systematic review and meta-analysis. PLoS ONE. 2020;15(10): e0241202. 33108375 10.1371/journal.pone.0241202 PMC7591022

[34] Wang W, Gu W, He C, Zhang T, Shen Y, Pu Y. Bioactive components of Banxia Xiexin Decoction for the treatment of gastrointestinal diseases based on flavor-oriented analysis. J Ethnopharmacol. 2022;291: 115085. 35150814 10.1016/j.jep.2022.115085

[35] Kono T, Satomi M, Chisato N, Ebisawa Y, Suno M, Asama T. Topical application of Hangeshashinto (TJ-14) in the treatment of chemotherapy-induced oral mucositis. World J Oncol. 2010;1(6):232–5. 29147213 10.4021/wjon263w PMC5649748

[36] Kono T, Kaneko A, Matsumoto C, Miyagi C, Ohbuchi K, Mizuhara Y. Multitargeted effects of Hangeshashinto for treatment of chemotherapy-induced oral mucositis on inducible prostaglandin E2 production in human oral keratinocytes. Integr Cancer Ther. 2014;13(5):435–45. 24501112 10.1177/1534735413520035

[37] Aoyama T, Nishikawa K, Takiguchi N, Tanabe K, Imano M, Fukushima R. Double-blind, placebo-controlled, randomized phase II study of TJ-14 (Hangeshashinto) for gastric cancer chemotherapy-induced oral mucositis. Cancer Chemother Pharmacol. 2014;73(5):1047–54. 24652604 10.1007/s00280-014-2440-x PMC4000413

[38] Matsuda C, Munemoto Y, Mishima H, Nagata N, Oshiro M, Kataoka M. Double-blind, placebo-controlled, randomized phase II study of TJ-14 (Hangeshashinto) for infusional fluorinated-pyrimidine-based colorectal cancer chemotherapy-induced oral mucositis. Cancer Chemother Pharmacol. 2015;76(1):97–103. 25983022 10.1007/s00280-015-2767-y PMC4485889

[39] Yamashita T, Araki K, Tomifuji M, Kamide D, Tanaka Y, Shiotani A. A traditional Japanese medicine-Hangeshashinto (TJ-14)-alleviates chemoradiation-induced mucositis and improves rates of treatment completion. Support Care Cancer. 2015;23(1):29–35. 24943276 10.1007/s00520-014-2315-z

[40] Rugge M, Sugano K, Sacchi D, Sbaraglia M, Malfertheiner P. Gastritis: an Update in 2020. Curr Treat Opt Gastroenterol. 2020;18:488–503.

[41] Fang C, Huang Y, Liu L, Yang H, Liu W, Fang Z. Clinical study of staged comprehensive treatment of Banxia Xiexin Decoction in treating refractory helicobacter pylori infection. Acta Chin Med Pharmacol. 2021;49(11):78–82.

[42] Qu Z, Yu M, Liu C. Clinical study on 98 cases of Banxiaxiexin Decoction for *H.**Pylori*-related positive gastritis. J Beihua Univ Nat Sci. 2017;18(03):368–70.

[43] Li L, Kong F, Shen J, Li D, Zhou H, Zhu Y. Clinical study on the relationship between Banxia Xiexin Decoction syndrome and HP infection. J Tradit Chin Med. 1998;1998(04):220–1.

[44] Yuan J. Clinical observation on 64 cases of *Helicobacter**pylori*-related gastritis treated with Banxia Xiexin Decoction. Yunnan J Trad Chin Med Materia Medica. 2002;2002(02):23.

[45] Hu C. Clinical observation on 72 cases of banxiaxiexin decoction for *H.**Pylori*-related positive gastritis. New Chin Med. 1994;1994(03):20–1.

[46] Lin C. Clinical effect and effective effect analysis of Banxia Xiexin Decoction in the treatment of *Helicobacter**pylori*-positive chronic gastritis. China Foreign Med Treat. 2019;38(21):155–7.

[47] Yang L. Clinical observation on Banxia Xiexin Decoction in treating HP-related gastritis. Chin Med Mod Distance Educ China. 2022;20(03):92–3.

[48] Chen Y. Clinical observation on Banxia Xiexin Decoction in the treatment of chronic gastritis with *Helicobacter**pylori* positive. Chin Med Mod Distance Educ China. 2023;21(07):99–101.

[49] Gao L. Clinical effect of Banxia Xiexin Decoction on chronic atrophic gastritis. Shenzhen J Integr Tradit Chin Western Med. 2019;29(10):53–4.

[50] Wu Y, Chen W, Yang J. Clinical study on Banxia Xiexin Decoction for treating chronic atrophic gastritis. China Pract Med. 2009;4(12):175–7.

[51] Li S. Clinical study on Banxia Xiexin Decoction for treating chronic atrophic gastritis. Henan Tradit Chin Med. 2015;35(01):26–7.

[52] Wang Q. Clinical effect observation of Banxia Xiexin Decoction in treating 40 cases of chronic atrophic gastritis. Inner Mongolia J Tradit Chin Med. 2016;35(17):30.

[53] Yuan Q. Clinical observation of Banxia Xiexin Decoction for treating chronic atrophic gastritis. World Latest Med Inf. 2017;17(02):147.

[54] Zhang Z. Clinical observation of Banxia Xiexin Decoction for treating chronic atrophic gastritis. J Electrocardiogr. 2018;7(02):254–6.

[55] Lu X. Clinical efficacy of Banxia Xiexin Decoction in the treatment of chronic atrophic gastritis. Res Integr Tradit Chin Western Med. 2017;9(06):295–6.

[56] Bi L. Clinical analysis of Banxia Xiexin Decoction in the treatment of chronic atrophic gastritis. J Snake. 2015;27(03):265–6.

[57] Zhang L, Xu P. Clinical effect analysis of Banxia Xiexin Decoction in the treatment of chronic atrophic gastritis. China Continuing Med Educ. 2018;10(09):122–4.

[58] Luo X, Li L. Clinical observation of Banxia Xiexin Decoction for treating chronic atrophic gastritis. J Liaoning Univ Tradit Chin Med. 2000;2000(02):124–5.

[59] M C, Clinical research of Pinelliae Decoction for purging stomach-fire to healing chronic atrophic gastritis. 2005: Tianjin University of Traditional Chinese Medicine.

[60] Kong X, Xu J, Li Q, Zhao S. Clinical observation of Banxia Xiexin Decoction in treating 20 cases of chronic atrophic gastritis. Mod Med J China. 2011;13(10):84–5.

[61] Qu W. Clinical observation of Banxia Xiexin Decoction in treating 42 cases of chronic atrophic gastritis. Clin J Chin Med. 2016;8(09):95–6.

[62] Cao Y. Clinical study on Banxia Xiexin Decoction in the treatment of chronic atrophic gastritis with spleen-stomach dampness syndrome. Guangming J Chin Med. 2021;36(07):1102–5.

[63] Liu Q. Clinical study on Banxia Xiexin Decoction in the treatment of chronic atrophic gastritis with spleen-stomach dampness syndrome. Guangming J Chin Med. 2022;37(20):3736–8.

[64] Yang L. Clinical observation on 50 cases of Banxiaxiexin Decoction for superficial gastritis. China’s Naturopathy. 2017;25(12):39–40.

[65] Liu H, Wang L. Observation on Banxia Xiexin Decoction in treating superficial gastritis. J Pract Tradit Chin Med. 2019;35(04):394–5.

[66] Zhao Q. Clinical observation on 32 cases of Banxiaxiexin Decoction for superficial gastritis. China Foreign Med Treat. 2010;29(07):127.

[67] Yuan C. Clinical study on Banxia Xiexin Decoction for treating chronic atrophic gastritis. Liaoning J Tradit Chin Med. 2007;2007(11):1583–4.

[68] Li X, Xu J, Wang X, Liao L, Huang L, Huang Y. Banxiaxiexin decoction treating gastritis mice with drug-resistant *Helicobacter**pylori* and its mechanism. World J Gastroenterol. 2023;29(18):2818–35. 37274067 10.3748/wjg.v29.i18.2818 PMC10237109

[69] Ji W, Wang T, Xu Y, An R, Liang K, Wang X. Identifying the active compounds and mechanism of action of Banxia Xiexin Decoction for treating ethanol-induced chronic gastritis using network pharmacology combined with UPLC–LTQ–Orbitrap MS. Comput Biol Chem. 2021;93: 107535. 34217946 10.1016/j.compbiolchem.2021.107535

[70] Ma Z, Chen X, Xiong M, Wang H, Sun C, Tang W, et al. Cyberpharmacology uncover the mechanism of the total Rhizoma Coptidis extracts ameliorate chronic atrophic gastritis. J Ethnopharmacol. 2024;335: 118644. 39094758 10.1016/j.jep.2024.118644

[71] Zhou Z, Hu C, Cui B, You L, An R, Liang K, et al. Ginsenoside Rg1 suppresses pyroptosis via the NF-kappaB/NLRP3/GSDMD pathway to alleviate chronic atrophic gastritis in vitro and in vivo. J Agric Food Chem. 2024;72:13668–83. 10.1021/acs.jafc.4c01271 38855973

[72] Zhou Y, Wang Q, Tang W, Ma Z, Yang Z, Li X, et al. *Palmatine**ameliorates* N-methyl-N’-nitrosoguanidine-induced chronic atrophic gastritis through the STAT1/CXCL10 axis. FASEB J. 2024;38(18): e70037. 39287361 10.1096/fj.202401624R

[73] Lanas A, Chan FKL. Peptic ulcer disease. Lancet. 2017;390(10094):613–24. 28242110 10.1016/S0140-6736(16)32404-7

[74] Del Valle J. Peptic ulcer and related disorders. New York: McGraw-Hill Education; 2020.

[75] Wang P. Clinical observation of peptic ulcer treated with Banxia Xiexin Decoction. China Pract Med. 2010;5(36):156.

[76] Wang H. Clinical observation of peptic ulcer treated with Banxia Xiexin Decoction. Guangming J Chin Med. 2011;26(08):1576–7.

[77] Li B. Clinical observation of peptic ulcer treated with Banxia Xiexin Decoction. J China Prescript Drug. 2014;12(4):124.

[78] Ding L. Clinical observation of peptic ulcer treated with Banxia Xiexin Decoction. Med J Chin People’s Health. 2018;30(13):73–5.

[79] Sun Y, Zhao F, Liu J. Clinical observation of peptic ulcer treated with Banxia Xiexin Decoction. China Pract Med. 2021;37(07):1104–5.

[80] Liang P, Liu Y. Clinical effect of Banxia Xiexin Decoction on peptic ulcer. Clin Res Pract. 2020;5(03):155–6.

[81] Fang Y. Clinical analysis on the treatment of peptic ulcer by Banxia Xiexin Decoction. Guangming J Chin Med. 2011;26(01):97–8.

[82] Li F. Clinical effect analysis on the treatment of peptic ulcer by Banxia Xiexin Decoction. China Med Herald. 2010;7(19):102–3.

[83] Zhao X. Clinical observation and mechanism study of traditional Chinese medicine Gufang Banxia Xiexin Decoction in treating patients with gastric ulcer. Guide of China Med. 2020;18(10):193–4.

[84] Zhuang J. Treatment of sleep disorder in peptic ulcer patients by banxiaxiexin prescription. World J Sleep Med. 2014;1(02):93–5.

[85] Pan S, Yu X, Liu M, Liu J, Wang C, Zhang Y, et al. Banxia Xiexin decoction promotes gastric lymphatic pumping by regulating lymphatic smooth muscle cell contraction and energy metabolism in a stress-induced gastric ulceration rat model. J Ethnopharmacol. 2024;328: 118015. 38499261 10.1016/j.jep.2024.118015

[86] Chen S, Huang Y, Wan S, Huang Y, Liang H, Chen S. Effect of Banxia Xiexin decoction on *Helicobacter**pylori*-related peptic ulcers and its possible mechanism via the TGF-beta/Smad signaling pathway. J Tradit Chin Med. 2018;38(3):419–26. 32185975

[87] Liu Y, Tan DY, Luo GX, Li DX, Ying Y, Guo CX. A study of Banxiaxiexin Decoction in preventing stress-induced gastric mucosal injury and its effects on Bcl-2 and Caspase-3. J Hunan Univ Chin Med. 2015;35(05):17–20.

[88] Ji W, Zhuang X, Hu C, Zhang Y. Revealing the active compounds and mechanism of Banxia Xiexin Decoction against gastric ulcer by network pharmacology and molecular docking. Nat Prod Commun. 2022. 10.1177/1934578X221118.

[89] Meng M, Wang S, Zhang S. Interpretation of the experts consensus on traditional Chinese medicine diagnosis and treatment of functional dyspepsia (2023). China J Tradit Chin Med Pharm. 2024;32(07):564–7.

[90] Zhang L, Cai H, Sun Z. Clinical study of Banxia Xiexin Decoction in the treatment of functional dyspepsia. J Math Med. 2019;32(10):1519–21.

[91] Yang L. Clinical observation of Banxia Xiexin Decoction for treating functional dyspepsia. Yunnan J Tradit Chin Med Materia Medica. 2019;40(08):100–1.

[92] Chu C, Yang L. Clinical observation on Banxia Xiexin Decoction in the treatment of functional dyspepsia. Guangming J Chin Med. 2019;34(10):1530–2.

[93] Zhao L, Su X. Clinical observation on Banxia Xiexin Decoction in the treatment of functional dyspepsia. Guangming J Chin Med. 2017;32(10):1432–4.

[94] Lang H, Chen F. Clinical observation of using Banxia Xiexin Decoction in the treatment of functional dyspepsia. J Sichuan Tradit Chin Med. 2015;33(09):90–2.

[95] Hu X, Zhu D, Zhou H, Xiong X. Clinical study on Banxia Xiexin Decoction for treating functional dyspepsia with cold-heat complex. J Hunan Univ Chin Med. 2006;2006(01):40–1.

[96] Liu C. Clinical observation of Banxia Xiexin Decoction for treating functional dyspepsia with cold-heat complex. Guangming J Chin Med. 2020;35(04):482–4.

[97] Zhang L, Cai Y, Liu X. Clinical effect and safety observation on Banxia Xiexin Decoction in the treatment of functional dyspepsia in the elderly. Chin J Clin Rational Drug Use. 2019;12(07):88–9.

[98] Yi P. Clinical effect of Banxia Xiexin Decoction in the treatment of children with dyspepsia. China Mod Med. 2020;27(14):172–4.

[99] Dang X. Clinical observation of Banxia Xiexin Decoction for treating non-ulcerative dyspepsia. Pract Clin J Integr Tradit Chin Western Med. 2005;2005(02):38–9.

[100] Kim S, Joung J, Ahn Y, Jung I, Son C. Beneficial potential of Banha-Sasim-Tang for Stress-sensitive functional dyspepsia via modulation of ghrelin: a randomized controlled trial. Front Pharmacol. 2021;12: 636752. 33959008 10.3389/fphar.2021.636752 PMC8093827

[101] Park J-W, Ko S-J, Han G, Yeo I, Ryu B, Kim J. The effects of Banha-sasim-tang on dyspeptic symptoms and gastric motility in cases of functional dyspepsia: a randomized, double-blind, placebo-controlled, and two-center trial. Evid Based Complement Altern Med. 2013;2013: 265035. 10.1155/2013/265035 PMC3686053 23861702

[102] Kim Y, Kim J, Jung S, Kwon O, Lee J, Son C. Efficacy of Banha-sasim-tang on functional dyspepsia classified as excess pattern: study protocol for a randomized controlled trial. Trials. 2017;18(1):1–7. 29121988 10.1186/s13063-017-2282-z PMC5679389

[103] Kim Y, Kim J, Kwon O, Jung S, Joung J, Yang C. Efficacy of a traditional herbal formula, Banha-Sasim-Tang in functional dyspepsia classified as excess pattern. Front Pharmacol. 2021;12: 698887. 34512334 10.3389/fphar.2021.698887 PMC8429799

[104] Park J-W, Ryu B, Yeo I, Jerng U-M, Han G, Oh S, et al. Banha-sasim-tan as an herbal formula for the treatment of functional dyspepsia: a randomized, double-blind, placebo-controlled, two-center trial. Trials. 2010;11(1):83. 20670451 10.1186/1745-6215-11-83 PMC2922084

[105] Zhu J, Li Y, Wang Q, Wang Q, Zeng H. Effects of Banxia Xiexin Decoction on gastric emptying and plasma gastric actin in rats with functional dyspepsia. China J Tradit Chin Med Pharm. 2005;2005(06):335–7.

[106] Liu H, Zhu Y, Wang G. Effects of Banxia Xiexin Decoction on gastrointestinal motility and related protein levels in rats with functional dyspepsia. Zhejiang J Tradit Chin Med. 2019;54(07):483–4.

[107] Dai L, Xhen Q, Liu X, Lin Y, Li K, Yue J. Banxia Xiexintang and its disassembled prescriptions regulate colonic mucosal immunity in young rats with flora disorder. Chin J Exp Tradit Med Formulae. 2022;28(11):42–50.

[108] Jeon Y-J, Lee J-S, Cho Y-R, Lee S-B, Kim W-Y, Roh S-S. Banha-sasim-tang improves gastrointestinal function in loperamide-induced functional dyspepsia mouse model. J Ethnopharmacol. 2019;238: 111834. 30940567 10.1016/j.jep.2019.111834

[109] Xing D, Dong Y, Liang Y, Deng S, Huang R. Effects of Banxia Xiexin Decoction on the ultrastructure of ICCs in rats with FD. J Guangdong Pharm Univ. 2012;28(03):336–8.

[110] Ren L, Ruan X, Dong H, Cheng Y, Shon K, Chang C, et al. The bitter flavor of Banxia Xiexin decoction activates TAS2R38 to ameliorate low-grade inflammation in the duodenum of mice with functional dyspepsia. J Ethnopharmacol. 2025;341: 119309. 39746410 10.1016/j.jep.2024.119309

[111] Aregawi LG, Shokrolahi M, Gebremeskel TG, Zoltan C. The effect of ginger supplementation on the improvement of dyspeptic symptoms in patients with functional dyspepsia. Cureus. 2023;15(9): e46061. 37771933 10.7759/cureus.46061 PMC10525921

[112] Xie P, Wu J, Chen G, Huang Y. Clinical observation on the treatment of diabetic gastroparesis with cold-heat mismatch by Banxia Xiexin Decoction. China’s Naturopathy. 2022;30(15):73–6.

[113] Li N, Duan C, Hu M. Clinical effect of Banxia Xiexin decoction in treatment of diabetic gastroparesis: an analysis of 31 cases. Hunan J Tradit Chin Med. 2020;36(2):1–3.

[114] Liu B. Clinical observation on Banxia Xiexin Decoction in treatment of diabetic gastroparesis. J Aerospace Med. 2018;29(09):1141–2.

[115] Zhao J, Zhang J. Clinical observation on Ban Xia Xie Xin Decoction in treating diabetic gastroparesis. Western J Tradit Chin Med. 2014;27(11):104–6.

[116] Han S, Li S, Li J. Clinical observation on Banxia Xiexin Decoction in the treatment of diabetes complicated with gastroparesis. Guangming J Chin Med. 2018;33(01):59–61.

[117] Zhang Y, Zheng G, Zhang J. Clinical study on Banxia Xiexin Decoction for treating diabetic non-alcoholic fatty liver disease. Psychol Mag. 2019;14(03):165.

[118] Zou J, Pan X. Clinical observation on Banxia Xiexin Decoction in treating diabetic gastroparesis. Inner Mongolia J Tradit Chin Med. 2009;28(14):6.

[119] Zhang Z. Analysis of the clinical effects of Banxia Xiexin Decoction in the treatment of non-alcoholic fatty liver with diabetes. Diabetes New World. 2020;23(21):89–91.

[120] Xu M. Clinical observation on the treatment of type 2 diabetes mellitus with spleen deficiency and gastric stagnation with Banxia Xiexin Decoction. Guangming J Chin Med. 2021;36(06):933–4.

[121] Li J, Qian W, Qing N. Effects of Banxia Xiexin Decoction on glucose and lipid metabolism and insulin secretion in diabetic mice. Beijing J Tradit Chin Med. 2021;40(03):246–9.

[122] Li Z, Ding N, Ren S. Effect of Banxia Xiexin Decoction on gastric emptying, AGEs content in gastric tissue and expression of RAGE and nNOS proteins in diabetic gastroparesis model mice. J Tradit Chin Med. 2022;63(24):2375–81.

[123] Xu C, Yue S, Lv X, Wang S, Chen Y. Study on the mechanism of Banxia Xiexin Decoction regulating blood glucose homeostasis through FXR/GLP-1 pathway based on bile acid metabolism profile. China J Tradit Chin Med Pharm. 2022;37(08):4394–9.

[124] Yang H, Li J, Song X, Cai J, Zhou L, Jiang G. Protective effect of Banxia Xiexin Decoction on pre-diabetic rats induced by 3-deoxyglucosone. Chin J Clin Pharmacol. 2022;38(11):1211–4.

[125] Li J, Yang H, Song X, Zhou L, Jiang G. Effects of Banxia Xiexintang on gut microbiota in prediabetic rats induced by 3-deoxyglucosone based on high-throughput sequencing. Chin J Clin Pharmacol. 2022;38(02):132–6.

[126] Tong S. Clinical research on the treatment of reflux oesophagitis by acridness relieving superficies and bitterness expelling internal heat method. Asia-Pacific Tradit Med. 2017;13(14):113–4.

[127] Liu D. Clinical observation on Banxia Xiexin Decoction in the treatment of reflux esophagitis of intermingled cold and heat type. Chin Med Mod Dist Educ China. 2022;20(21):76–7.

[128] Li H, Zhou Z, Huang H. Clinical observation on the treatment of reflux oesophagitis with Banxia Xiexin Decoction. Shenzhen J Integr Tradit Chin Western Med. 2021;31(07):61–2.

[129] Liu Y, Chen H. Clinical observation on 36 cases of reflux oesophagitis treated with Banxia Xiexin Decoction. Liaoning J Tradit Chin Med. 2006;2006(04):436.

[130] Wang J, Zhou W, Gu R, Song G, Liu H. Clinical observation on the treatment of gastro-oesophageal reflux disease with Banxia Xiexin Decoction. J Emerg Tradit Chin Med. 2016;25(09):1828–9.

[131] Tan L. Clinical observation on treating gastroesophageal reflux disease with Banxia Xiexin Decoction. Clin J Chin Med. 2014;6(16):103–4.

[132] Li D, Gu Z. Observation on the efficacy of Banxia Xiexin Decoction in the treatment of gastroesophageal reflux disease. Shaanxi J Tradit Chin Med. 2017;38(07):903–4.

[133] Li J. Clinical observation on 113 cases of gastroesophageal reflux treated with Banxia Xiexin Decoction. Clin J Chin Med. 2014;6(03):109–10.

[134] Sun L, Wang Y. Clinical study on modified Banxia Xiexin Decoction in the treatment of gastroesophageal reflux cough. Guangming J Chin Med. 2020;35(16):2489–91.

[135] Zou Y, Lv H, Cheng S. Clinical observation on the treatment of bile reflux gastritis with Banxia Xiexin Decoction. Clin J Tradit Chin Med. 2007;2007(02):139–40.

[136] Huang Y. Clinical observation on the treatment of patients with superficial gastritis with bile reflux and anxiety symptoms by Banxia Xiexin Decoction. Mod Intervent Diagn Treat Gastroenterol. 2007;2007(02):114–5.

[137] Takeuchi T, Hongo H, Kimura T, Kojima Y, Harada S, Ota K. Efficacy and safety of hangeshashinto for treatment of GERD refractory to proton pump inhibitors: Usual dose proton pump inhibitors plus hangeshashinto versus double-dose proton pump inhibitors: randomized, multicenter open label exploratory study. J Gastroenterol. 2019;54(11):972–83. 31037449 10.1007/s00535-019-01588-4

[138] Jin X, Li Y, Liu G, Sun W. Study on the treatment effect of Bx Decoction on rat models of reflux esophagitis. J Radioimmunol. 2008;2008(04):312–4.

[139] Liu X, Gao Y, Si Y, Niu X. The influence of Banxiaxiexin decoction and its analogous preparations on neurotensin (NT) in rat models with reflux esophagitis. J Radioimmunol. 2003;16(04):215–7.

[140] Choo BK, Roh SS. Berberine protects against esophageal mucosal damage in reflux esophagitis by suppressing proinflammatory cytokines. Exp Ther Med. 2013;6(3):663–70. 24137243 10.3892/etm.2013.1202 PMC3786780

[141] Nakase H, Uchino M, Shinzaki S, Matsuura M, Matsuoka K, Kobayashi T. Evidence-based clinical practice guidelines for inflammatory bowel disease 2020. J Gastroenterol. 2021;56(6):489–526. 33885977 10.1007/s00535-021-01784-1 PMC8137635

[142] Wang P. Observation on the therapeutic effect of modified Banxia Xiexin Decoction combined with traditional Chinese medicine retention enema in the treatment of ulcerative colitis. Contemp Med. 2013;19(19):153–4.

[143] Bian Y, Zhang S. Observation on the therapeutic effect of Banxia Xiexin Decoction in treating gastric ulcer of mixed cold and heat type and chronic ulcerative colitis. Jilin J Chin Med. 2013;33(06):588–9.

[144] Sun X, Du J, Sun Y. Meta-analysis of efficacy and safety of Banxia Xiexin Decoction in treatment of ulcerative colitis. Drug Eval Res. 2023;46(4):878–84.

[145] Zhang H. Observation on the therapeutic effect of Banxiaxiexin Decoction in the treatment of 40 cases of ulcerative colitis. Asia-Pacific Tradit Med. 2016;12(02):109–10.

[146] Chen J, Zhang L, Gu W, Wu X, Zhang X, Han T. Effect of Banxie Xiexin Decoction on gut microbiota and th17/treg cell balance in mice with ulcerative colitis induced by dextran sodium sulfate. Mod Tradit Chin Med Materia Medica World Sci Technol. 2022;24(07):2695–709.

[147] Zhang Y. Influences of Banxia Xiexin Decoction and split prescriptions on Th1/Th2 cytokines of rats with ulcerative colitis. Jinan: Shandong University of Traditional Chinese Medicine; 2015.

[148] Mou Y, Min D, Li S, Xie T, Luo W. Effect of Banxia Xiexin Decoction on expression of TNF-α, IL-6 and PI3K/Akt signaling pathway in rats with ulcerative colitis. J Liaoning Univ Tradit Chin Med. 2023;25(09):29–31.

[149] Zhang L, Gu W, Wu T, Li L, Wu X, Zhang X, et al. Banxia Xiexin Decoction inhibited ulcerative colitis in mice by inhibiting inflammation. Tianjin J Tradit Chin Med. 2023;40(02):202–13.

[150] Yang G, Sun J, Wang L, Jiang X, Zheng Y. Banxia Xiexin Decoction inhibits secretion of inflammatory cytokines in macrophages via MAPK signaling pathway. Acad J Shanghai Univ Tradit Chin Med. 2018;32(05):67–72.

[151] Zhao Z, Liu L, Song N, Yang Y, Han X. Effect of Banxia Xiexintang on NLRP3/Caspase-1 pyroptosis pathway in rats with ulcerative colitis. Chin J Exp Tradit Med Formulae. 2022;28(16):29–34.

[152] Li S, Zhang L, Mu Y, Zhu T, Luo W, Wang J, et al. Experimental study of Banxia Xiexin Decoction regulating TLRs/NF-κB/MyD88 signaling pathway in the intervention of ulcerative colitis. Mod Tradit Chin Med Materia Medica World Sci Technol. 2023;25(03):1133–9.

[153] Xu F, Mao Y, Zhou S, Shang H. Protective effect of Banxia Xiexin Decoction on intestinal mucosal barrier function and expression of ZO-1 and occludin in mice with ulcerative colitis. J Basic Chin Med. 2019;25(01):44–7.

[154] Cui L, Zhang Y, Zheng Z, Song X, Han T. Intervention of bone marrow mesenchymal stem cell transplantation with Banxia Xiexin Decoction in the treatment of ulcerative colitis in rats. Chinese Society of Traditional Chinese Medicine, Experimental Pharmacology of Chinese Medicines Division; 2014. p. 52–3.

[155] Gwee K, Bak Y, Ghoshal U, Gonlachanvit S, Lee O, Fock K. Asian consensus on irritable bowel syndrome. J Gastroenterol Hepatol. 2010;25(7):1189–205. 20594245 10.1111/j.1440-1746.2010.06353.x

[156] Zhan C, Pan F, Zhang T. A clinical study on Banxia Xiexin Decoction treating diarrhea predominated irritable bowel syndrome by observing the expression of ghrelin. Chin Arch Tradit Chin Med. 2011;29(11):2588–91.

[157] Liu T, Chen X, Miao W, Zhong L. Clinical observation of Banxia Xiexin Tang for diarrhea-predominant of irritable bowel syndrome. New Chin Med. 2016;48(08):76–9.

[158] Li D. Clinical study on 56 cases of Banxiaxiexin Decoction for irritable bowel syndrome. Chin J Tradit Med Sci Technol. 1998;1998(01):45.

[159] Zeng Y, Lian X, Lin L. Clinical effect observation on Ban Xia Xie Xin Decoction in treating irritable bowel syndrome. China Med Herald. 2009;6(19):127–8.

[160] Li B, Rui J, Ding X, Yang X. Exploring the multicomponent synergy mechanism of Banxia Xiexin Decoction on irritable bowel syndrome by a systems pharmacology strategy. J Ethnopharmacol. 2019;233:158–68. 30590198 10.1016/j.jep.2018.12.033

[161] Lu Y, Huang J, Zhang Y, Huang Z, Yan W, Zhou T, et al. Therapeutic effects of berberine hydrochloride on stress-induced diarrhea-predominant irritable bowel syndrome rats by inhibiting neurotransmission in colonic smooth muscle. Front Pharmacol. 2021;12: 596686. 34594213 10.3389/fphar.2021.596686 PMC8476869

[162] Xiong Y, Wei H, Chen C, Jiao L, Zhang J, Tan Y, et al. Coptisine attenuates post-infectious IBS via Nrf2-dependent inhibition of the NLPR3 inflammasome. Mol Med Rep. 2022;26(6):362. 36281933 10.3892/mmr.2022.12879

[163] Zhao B, Ye J, Zhao W, Liu X, Lan H, Sun J, et al. 6-Shogaol derived from ginger inhibits intestinal crypt stem cell differentiation and contributes to irritable bowel syndrome risk. Research (Wash D C). 2024;7:0524. 39512446 10.34133/research.0524 PMC11542252

[164] Takahashi M, Nakajima M, Muroi H, Satomura H, Domeki Y, Ihara K. Prevention of the chemotherapy-induced oral mucositis in esophageal cancer by use of Hangeshashinto (TJ-14). Int Surg. 2018;103(7–8):401–8.

[165] Taira K, Fujiwara K, Fukuhara T, Koyama S, Takeuchi H. The effect of Hangeshashinto on oral mucositis caused by induction chemotherapy in patients with head and neck cancer. Yonago Acta Med. 2020;63(3):183–7. 32884437 10.33160/yam.2020.08.007 PMC7435114

[166] Nishikawa K, Aoyama T, Oba M, Yoshikawa T, Matsuda C, Munemoto Y. The clinical impact of Hangeshashinto (TJ-14) in the treatment of chemotherapy-induced oral mucositis in gastric cancer and colorectal cancer: Analyses of pooled data from two phase II randomized clinical trials (Hangesha-G and Hangesha-C). J Cancer. 2018;9(10):1725–30. 29805697 10.7150/jca.24733 PMC5968759

[167] Yoshimatsu M, Kawashita Y, Soutome S, Murata M, Sawayama Y, Kurogi T. Hangeshashinto for prevention of oral mucositis in patients undergoing hematopoietic stem cell transplantation: a randomized phase II study. Support Care Cancer. 2023;31(12):375. 37979045 10.1007/s00520-023-08175-7 PMC10657322

[168] Kato T, Sakagami H. Efficacy of cryotherapy and Hangeshashinto for radiation-induced oral stomatitis: preliminary study. In Vivo. 2023;37(2):830–5. 36881064 10.21873/invivo.13149 PMC10026662

[169] Hitomi S, Ono K, Terawaki K, Matsumoto C, Mizuno K, Yamaguchi K, et al. [6]-gingerol and [6]-shogaol, active ingredients of the traditional Japanese medicine hangeshashinto, relief oral ulcerative mucositis-induced pain via action on Na(+) channels. Pharmacol Res. 2017;117:288–302. 28043879 10.1016/j.phrs.2016.12.026

[170] Pakravan F, Salehabad NH, Karimi F, Isfahani MN. Comparative study of the effect of licorice muco-adhesive film on radiotherapy induced oral mucositis, a randomized controlled clinical trial. Gulf J Oncolog. 2021;1(37):42–7. 35152194

[171] Li Y, Qiu S, Liang J. Clinical study on Banxia Xiexin Decoction in treatment of disharmony between liver and spleen after colorectal cancer surgery. World Chin Med. 2017;12(07):1523–6.

[172] Li J, Li H, Qi R, Lv S. Clinical study on treatment of stuffiness of stomach of cold-heat complicated syndrome in gastric precancerous lesions with Ban Xia Xie Xin Decoction. Xinjiang Tradit Chin Med. 2022;40(03):4–7.

[173] Ichiki M, Wataya I, Yamada K, Tsuruta N, Takeoka H, Okayama Y. Preventive effect of kampo medicine (Hangeshashin-to, TJ-14) plus minocycline against afatinib-induced diarrhea and skin rash in patients with non-small cell lung cancer. Oncotargets Ther. 2017;10:5107–13. 10.2147/OTT.S145613 PMC5661491 29123409

[174] He J. Clinical observation on the prevention and treatment of gastrointestinal reactions induced by chemotherapy of gastrointestinal tumour with Banxia Xiexin Decoction. J Emerg Tradit Chin Med. 2010;19(04):581–2.

[175] Wang L, Ke J, Wang C, Li Y, Wu G, Ding Q. Efficacy and safety of Banxia Xiexin Decoction, a blended traditional Chinese medicine, as monotherapy for patients with advanced hepatocellular carcinoma. Integr Cancer Ther. 2020. 10.1177/1534735420942587. 32787468 10.1177/1534735420942587 PMC7427017

[176] Sun Z. Clinical study on the treatment of colon cancer with the combination of acridness relieving superficies and bitterness expelling internal heat method and Banxia Xiexin Decoction. Asia-Pacific Tradit Med. 2017;13(08):125–6.

[177] Li K, Liu R, Xu G, Zhang W, Liu C, Zhu B. Effect of a modified Banxia Xiexin Decoction plus chemotherapy on stage III colon cancer. J Tradit Chin Med. 2019;39(2):251–7. 32186049

[178] Zhang Z, Wu C, Liu N, Wang Z, Pan Z, Jiang Y, et al. Modified Banxiaxiexin decoction benefitted chemotherapy in treating gastric cancer by regulating multiple targets and pathways. J Ethnopharmacol. 2024;331: 118277. 38697407 10.1016/j.jep.2024.118277

[179] Gou Y, Zhou H. Observation on the therapeutic effect of chemotherapy combined with Banxia Xiexin Decoction on duodenal cancer under CT enhanced scanning. Int J Radiat Res. 2023;21(4):50.

[180] Zu GX, Sun KY, Liu XJ, Tang JQ, Huang HL, Han T. Banxia xiexin decoction prevents the development of gastric cancer. World J Clin Oncol. 2024;15(10):1293–308. 39473858 10.5306/wjco.v15.i10.1293 PMC11514502

[181] Jiang Y, Huang Y, Hu Y, Yang Y, You F, Hu Q, et al. Banxia Xiexin Decoction delays colitis-to-cancer transition by inhibiting E-cadherin/beta-catenin pathway via Fusobacterium nucleatum FadA. J Ethnopharmacol. 2024;328: 117932. 38382652 10.1016/j.jep.2024.117932

[182] Feng X, Xue F, He G, Ni Q, Huang S. Banxia xiexin decoction affects drug sensitivity in gastric cancer cells by regulating MGMT expression via IL-6/JAK/STAT3-mediated PD-L1 activity. Int J Mol Med. 2021;48(2):1–11. 10.3892/ijmm.2021.4998 PMC8262654 34278452

[183] Kelley S, Skarra D, Rivera A, Thackray V. The gut microbiome is altered in a letrozole-induced mouse model of polycystic ovary syndrome. PLoS ONE. 2016;11(1): e0146509. 26731268 10.1371/journal.pone.0146509 PMC4701222

[184] Zheng D, Liu X, Zhao Y. Time-effect relationship study of modified Banxia Xiexin Decoction in the treatment of polycystic ovary syndrome insulin resistance (deficiency of the spleen and heat of the stomach). Glob Tradit Chin Med. 2017;10(02):220–4.

[185] Liu X, Chen R, Wen S, Liu J, Zhang L, Zheng D. Efficacy of modified Banxia Xiexin Decoction in treating PCOS hyperinsulinemia of stomach heat and spleen deficiency. Beijing J Tradit Chin Med. 2022;41(04):431–5.

[186] Wang L, Gu X, Chen J, Yu J, Sun Y, Ma Z. Clinical efficacy of modified Banxia Xiexin Decoction PCOS-IR patients with spleen-deficiency stomach-heat type and effect on serum IL-4. J Hebei Tradit Chin Med Pharmacol. 2023;38(06):48–52.

[187] Zhao F, Ding X, An M, Zhao Y, Liu J. Effect of Banxia Xiexin Decoction on intestinal microflora diversity of PCOS based on 16s rDNA sequencing. Chin J Microecol. 2022;34(10):1151–7.

[188] Zhao H, Chen R, Zheng D, Xiong F, Jia F, Liu J. Modified Banxia Xiexin Decoction ameliorates polycystic ovarian syndrome with insulin resistance by regulating intestinal microbiota. Front Cell Infect Microbiol. 2022;12: 854796. 35619648 10.3389/fcimb.2022.854796 PMC9127304

[189] Zhao H, Chen R, Zheng D, Jia F, Liu J, Liu Y. Effect of Jiawei Banxia Xiexin Decoction on gut microbiota and inflammatory factors in polycystic ovarian syndrome with insulin resistance model rats. J Tradit Chin Med. 2022;63(21):2072–80.

[190] Jia F, Zhao H, Zheng D, Chen R, Chen T, Yu Y. Therapeutic effect and mechanism of modified Banxia Xiexin Decoction in the treatment of polycystic ovary syndrome complicated with insulin resistance. J Beijing Univ Tradit Chin Med. 2023;46(08):1117–27.

[191] Gu W, Gu J, Wang J and Zhang L, Banxia Xiexin Decoction can regulate the TLR4/IL-6 signal axis by targeting miR-20b-5p and alleviate polycystic ovarian syndrome. Mol Cell Toxicol. 2025.

[192] He S, Li H, Zhang Q, Zhao W, Li W, Dai C, et al. Berberine alleviates inflammation in polycystic ovary syndrome by inhibiting hyaluronan synthase 2 expression. Phytomedicine. 2024;128: 155456. 38537446 10.1016/j.phymed.2024.155456

[193] Wang Z, Nie K, Su H, Tang Y, Wang H, Xu X, et al. Berberine improves ovulation and endometrial receptivity in polycystic ovary syndrome. Phytomedicine. 2021;91: 153654. 34333328 10.1016/j.phymed.2021.153654

[194] Pournaderi PS, Yaghmaei P, Khodaei H, Noormohammadi Z, Hejazi SH. The effects of 6-Gingerol on reproductive improvement, liver functioning and Cyclooxygenase-2 gene expression in estradiol valerate—induced polycystic ovary syndrome in Wistar rats. Biochem Biophys Res Commun. 2017;484(2):461–6. 28093231 10.1016/j.bbrc.2017.01.057

[195] Lu H, Qin J, Han N, Xie F, Gong L, Li C. Banxia Xiexin Decoction is effective to prevent and control irinotecan-induced delayed diarrhea in recurrent small cell lung cancer. Integr Cancer Ther. 2018;17(4):1109–14. 30229683 10.1177/1534735418801532 PMC6247536

[196] Murai T, Matsuo M, Tanaka H, Manabe Y, Takaoka T, Hachiya K. Efficacy of herbal medicine TJ-14 for acute radiation-induced enteritis: a multi-institutional prospective Phase II trial. J Radiat Res. 2020;61(1):140–5. 31691810 10.1093/jrr/rrz025 PMC7022136

[197] Sin S, Wu J, Kang Y, Yip K, Kong N, Wan H. Efficacy of modified Banxia Xiexin Decoction in the management of Wei-Pi syndrome (postprandial distress syndrome): study protocol for a randomized, waitlist-controlled trial. Trials. 2021;22(1):135. 33579349 10.1186/s13063-021-05078-y PMC7881573

[198] Kuwamura A, Komasawa N, Kori K, Tanaka M, Minami T. Preventive effect of preoperative administration of Hange-Shashin-To on postoperative sore throat: A prospective, double-blind, randomized trial. J Altern Complementary Med. 2015;21(8):485–8. 10.1089/acm.2014.0316 26087107

[199] Shi S. Clinical observation on the treatment of Helicobacter Pylori-associated halitosis with Banxia Xiexin Decoction. Inner Mongolia J Tradit Chin Med. 2014;46(31):33–4.

[200] Liu J, Li L, Ning N, Huang L. Effects of Banxia Xiexin Decoction on the expression of neurotransmitters and early fast gene in migraine model rats. Pharmacol Clin Chin Materia Medica. 2015;31(05):9–12.

[201] He C, Zhou J, Zhang W, Zhang T, Pu Y. Study on the bioactive components of Banxia Xiexin Decoction with different decocting methods and its effects on ulcerative colitis rats from the perspective of phase states. J Ethnopharmacol. 2024;335: 118626. 39053716 10.1016/j.jep.2024.118626

[202] Wang S, Leung C-H, Chen X, Li P, Lu J, Lu J, et al. Mechanism-oriented quality research system for traditional Chinese medicine. SCIENTIA SINICA Vitae. 2024. 10.1360/ssv-2024-0161.

