# Supplementary material for: Reunderstanding the classical prescription Banxia Xiexin Decoction: new perspectives from a comprehensive review of clinical research and pharmacological studies
Source: Chin Med. 2025 Mar 18;20:39. doi: 10.1186/s13020-025-01087-0 (PMC11921579; doi:10.1186/s13020-025-01087-0)
Supplement: Supplementary file 1 — Supplementary Material 1 [file 13020_2025_1087_MOESM1_ESM.docx]

Supplementary materials for

**Reunderstanding the Classical Prescription Banxia Xiexin Decoction: New Perspectives from a Comprehensive Review of Clinical Research and Pharmacological Studies**

Chang Liu^a,b,#^, Pengwei Gao^a^, Xiaoying Liu^a^, Min Kuang^c,d^, Haoran Xu^c,d^, Yangming Wu^c,d^, Wenjun Liu^c,d*^, Shengpeng Wang^a,b*^

**Supplementary Table 1. Representative clinical studies of BXD on chronic gastritis.**

| **Patients entered** | **Study design** | **Study length** | **Sample size** | **Observation Group** | | | **Main results** | **Reference** |
| --- | --- | --- | --- | --- | --- | --- | --- | --- |
|  |  |  |  | **Medicine Detail** | **Dosage** | **Preparation** | **Details** |  |
| chronic gastritis | Randomized | 4 weeks | 80 | Pinelliae Rhizoma 10 g, Coptidis Rhizoma 10 g, Scutellariae Radix 10 g, Magnoliae Officinalis Cortex 12 g, Toosendan Fructus 12 g, Codonopsis Radix 15 g, Zingiberis Rhizoma 6 g, Glycyrrhizae Radix et Rhizoma Praeparata cum Melle 5 g, 6 pieces of Jujubae Fructus.  For patients with liver and stomach Qi stagnation, remove Codonopsis Radix and Zingiberis Rhizoma, add Aucklandiae Radix 12 g, Stir-fried Aurantii Fructus Immaturus 12 g, Bupleuri Radix 15 g.  For patients with phlegm-heat interjunction, remove Codonopsis Radix and Zingiberis Rhizoma, add Forsythiae Fructus 12 g, Aurantii Fructus 12 g, Taraxaci Herba 15 g.  For patients with food stagnation, add Galli Gigerii Endothelium Corneum 12 g, Stir-fried Hordei Fructus Germinatus 12 g, Raphani Semen 9 g. | 1 dose/ d, 2 times/ d | Decoction | BXD combined with triple therapy has significant clinical efficacy in the treatment of chronic gastritis. It can effectively remove HP with few adverse reactions. The total effective rate was 92.5%, compared with 87.5% total effective rate in control group. The HP negative conversion rate was 87.5%, compared with 70.0% total effective rate in control group. | [1] |
| chronic gastritis | Randomized | 4 weeks | 88 | Pinelliae Rhizoma praeparatum 10 g, Codonopsis Radix 15 g, Zingiberis Rhizoma 3 g, 6 pieces of Jujubae Fructus, Bletillae Rhizoma 10 g, Scutellariae Radix 10 g, Glycyrrhizae Radix et Rhizoma 6 g, Stir-fried Aurantii Fructus Immaturus 10 g, Coptidis Rhizoma 3 g.  For patients with liver depression, add Citri Sarcodactylis Fructus 15 g.  For patients with dry stool, add Rhei Radix et Rhizoma 6 g.  For patients with limb weakness, remove Codonopsis Radix and add Ginseng Radix et Rhizoma 6 g.  For fidgety patients, add Glycyrrhizae Radix et Rhizoma 15 g. | 1 dose/ d, 2 times/ d, 75 ml/ time | Decoction | BXD adjustable treatments can relieve patients' symptoms, improve gastrointestinal motility, reduce inflammatory reactions, and reduce recurrence rates. The experiment evaluated TCM syndrome scores, clinical efficacy, levels of inflammatory factors and gastrointestinal hormones, adverse reactions and recurrence rates. The above indicators in the BXD treatment group were better than those in the control group, and the difference was statistically significant. | [2] |
| chronic gastritis with | Randomized | 1 month | 120 | Pinelliae Rhizoma Praeparatum Cum Alumine 10 g, Codonopsis Radix 10 g, Scutellariae Radix 10 g, Coptidis Rhizoma 5 g, Bupleuri Radix 10 g, Zingiberis Rhizoma 6 g, Curcumae Radix 12 g, Aurantii Fructus 10 g, Glycyrrhizae Radix et Rhizoma Praeparata cum Melle 6 g, 12 pieces of Jujubae Fructus.  For patients with shortness of breath and limb weakness, add Astragali Radix 15 g, Atractylodis Macrocephalae Rhizoma 10 g.  For patients with epigastric pain, add Corydalis Rhizoma 10 g, Toosendan Fructus 10 g.  For patients with gastric cavity cold, add Alpiniae Officinarum Rhizoma 5 g, Aconiti Lateralis Radix Praeparata 3 g. | 1 dose/ d, 2 times/ d | Decoction | BXD has significant clinical efficacy in treating chronic gastritis and can effectively improve gastrointestinal hormone levels. The total effective rate was 95.00%, compared with 76.67% total effective rate in control group. Comparing the gastrointestinal hormone levels of the two groups of patients after treatment, the GAS level of the BXD treatment group after treatment was significantly lower than that of the control group, while the MTL and SS levels were significantly higher than those of the control group, and the difference was statistically significant (P<0.05). | [3] |
| chronic gastritis | Randomized | 1 month | 120 | Codonopsis Radix 20 g, Processed Pinelliae Rhizoma 10 g, Scutellariae Radix 12 g, Coptidis Rhizoma 8 g, Jujubae Fructus 15 g, Atractylodis Macrocephalae Rhizoma 10 g, Glycyrrhizae Radix et Rhizoma Praeparata cum Melle 8 g, dioscoreae rhizoma 20 g, lablab semen album 20 g, Amomi Fructus 8 g, Shenqu 15 g, Taraxaci Herba 15 g, Scutellariae Barbatae Herba 15 g.  For severe spleen deficiency, add Astragali Radix and Atractylodis Macrocephalae Rhizoma.  For patients with dampness and heat in the spleen and stomach, add Herba Hedyotidis and Gardeniae Fructus.  For patients with spleen and stomach deficiency cold, add Zingiberis Rhizoma and Euodiae Fructus. | 1 dose/ d, 2 times/ d | Decoction | Modified BXD has a significant effect on the repair of gastric mucosa in chronic gastritis. The score of chronic inflammation in gastric mucosal pathological tissue was 1.01±0.15, compared with 1.29±0.67 score in control group. | [4] |
| chronic gastritis | Randomized | 12 weeks | 84 | Processed Pinelliae Rhizoma 12 g, Zingiberis Rhizoma 9 g, Scutellariae Radix 9 g, Coptidis Rhizoma 3 g, Glycyrrhizae Radix et Rhizoma Praeparata cum Melle 9 g, Codonopsis Radix 9 g, 12 pieces of Jujubae Fructus.  For severe heat, add Scutellariae Radix to 15 g and Coptidis Rhizoma to 10 g.  For severe cold, add Processed Pinelliae Rhizoma to 15 g and Zingiberis Rhizoma to 10 g. | 1 dose/ d, 2 times/ d, 125 ml/ time | Decoction | BXD can improve multiple clinical symptoms in patients with chronic gastritis. The experiment evaluated three aspects: patient treatment effect, incidence of adverse reactions, and clinical symptom scores before and after treatment. The indicators of the BXD treatment group were better than those of the control group, and the difference was statistically significant. The total effective rate was 90.4%, compared with 73.8% total effective rate in control group. The adverse reactions rate was 7.1%, compared with 23.8% adverse reactions rate in control group. | [5] |
| chronic gastritis with | Randomized | 4 weeks | 100 | Pinelliae Rhizoma 15 g, Zingiberis Rhizoma 15 g, Ginseng Radix et Rhizoma 6 g, Glycyrrhizae Radix et Rhizoma 6 g, Scutellariae Radix 10 g, Coptidis Rhizoma 10 g, 5 pieces of Jujubae Fructus.  For patients with nausea and vomiting, add Aurantii Fructus Immaturus.  For patients with dry stool, add Rhei Radix et Rhizoma.  For patients with Qi stagnation and liver depression, add Bupleuri Radix.  For patients with acid reflux, add Euodiae Fructus.  For patients with anorexia, add Stir-fried Hordei Fructus Germinatus.  For patients with bloating, add Curcumae Radix and Aucklandiae Radix. | 1 dose/ d, 2 times/ d | Decoction | The use of BXD in the treatment of patients with chronic gastritis can achieve good short-term and long-term effects. The experiment evaluated aspects such as the disappearance time of patients' clinical symptoms, comparison of HP negative turning time, comparison of inflammatory factor levels before and after treatment, comparison of recurrence of gastric disease after 6 months, and comparison of medication safety. Except for the fact that there was no statistically significant difference between the treatment group and the control group in terms of medication safety, the treatment group was proven to be more effective in the other three aspects. HP turned negative for 10.5 ± 2.8 days in the Chinese medicine group, compared with 15.2 ± 3.9 days in the western medicine group. | [6] |
| chronic gastritis | Randomized | 30 days | 86 | Codonopsis Radix 15 g, Bletillae Rhizoma 10 g, Pinelliae Rhizoma praeparatum 10 g, Scutellariae Radix 10 g, Stir-fried Aurantii Fructus Immaturus 10 g, Coptidis Rhizoma 3 g, Zingiberis Rhizoma 3 g, Glycyrrhizae Radix et Rhizoma 6 g, 6 pieces of Jujubae Fructus.  For patients with acid reflux, add Sepiae Endoconcha 15 g.  For patients with epigastric tingling pain, add Paeoniae Radix Alba 20 g.  For patients with limb weakness, add Ginseng Radix et Rhizoma 6 g and remove Codonopsis Radix.  For patients with irritability, liver depression and fire, irritability, add Prunellae Spica 15 g.  For patients with liver depression, add Citri Sarcodactylis Fructus 15 g.  For patients with dry stool, add Rhei Radix et Rhizoma 6 g. | 1 dose/ d, 2 times/ d, 75 ml/ time | Decoction | BXD can effectively improve the clinical symptoms of patients with chronic gastritis, protect the gastric mucosa, and reduce the occurrence of adverse reactions. The total effective rate was 95.35%, compared with 79.07% total effective rate in control group. The experiment tested the patients' pepsinogen I, pepsinogen ratio, pepsinogen II, and gastrin 17 levels before and after treatment, which showed that the indicators in the BXD treatment group were lower than those in the control group (p<0.05). | [7] |
| chronic gastritis | Randomized | 4 weeks | 108 | Pinelliae Rhizoma 12 g, Scutellariae Radix 9 g, Zingiberis Rhizoma 9 g, Ginseng Radix et Rhizoma 9 g, Glycyrrhizae Radix et Rhizoma Praeparata cum Melle 9 g, Coptidis Rhizoma 3 g, 12 pieces of Jujubae Fructus.  For patients with damp and heat, add Herba Hedyotidis 30 g, Taraxaci Herba 30 g.  For patients with Qi stagnation and liver depression, add Paeoniae Radix Alba 12 g, Bupleuri Radix 12 g.  For patients with symptoms of diarrhea, add Citri Reticulatae Pericarpium 15 g, Atractylodis Macrocephalae Rhizoma 20 g.  For patients with acid reflux, add Sepiae Endoconcha 30 g, Calcines Arcae Concha 30 g.  For patients with dyspepsia, add Stir-fried Alli Gigerii Endothelium Corneum 15 g, and Raphani Semen 15 g. | 1 dose/ d, 2 times/ d | Decoction | BXD in the treatment of chronic gastritis clinical effect is better than conventional medicine treatment. The total effective rate was 88.89%, compared with 72.22% total effective rate in control group. | [8] |
| chronic gastritis | Randomized | 1 month | 148 | Pinelliae Rhizoma 9 g, Zingiberis Rhizoma 10 g, Scutellariae Radix 6 g, Coptidis Rhizoma 6 g, Ginseng Radix et Rhizoma 10 g, 10 pieces of Jujubae Fructus, Glycyrrhizae Radix et Rhizoma 10 g.  For patients with belching, add Inulae Flos 12 g, Haematitum 15 g.  For those who vomit acid, add Calcines Arcae Concha 15 g.  For patients with indigestion, add Scorched Crataegi Fructus 12 g, Messa Medicata Fermentata 12 g, Stir-fried Hordei Fructus Germinatus 12 g. | 2 times/ d, 100 ml/ time, 100 ml/ time | Decoction | BXD has definite clinical efficacy in treating chronic gastritis. The total effective rate was 94.6%, compared with 82.4% total effective rate in control group. | [9] |
| gastritis | Randomized | 45 days | 80 | Pinelliae Rhizoma 12 g, Coptidis Rhizoma 3 g, Bambusae Caulis in Taenias 8 g, Aurantii Fructus 15 g, Aucklandiae Radix 10 g, Glycyrrhizae Radix et Rhizoma 6 g, Coptidis Rhizoma 4 g, Codonopsis Radix 15 g, Aurantii Fructus Immaturus 10 g, Bletillae Rhizoma 10 g, Scutellariae Radix 10 g, 6 pieces of Jujubae Fructus.  For patients with bloating, add Aspongopus 6 g, Arecae Pericarpium 12 g, Perillae Fructus 10 g.  For patients with nausea and vomiting, add Ginger-fried Banbusae Caulis in Taenias 9 g, Inulae Flos 9 g.  For patients with waterbrash, add Euodiae Fructus 3 g, Calcines Arcae Concha 20 g, Sepiae Endoconcha 15 g.  For patients with borborygmus and diarrhea, add Jiao Sanxian (Fried Hordei Fructus Germinatus, Fried Crataegi Fructus, Fried Messa Medicata Fermentata) 15 g, Aucklandiae Radix 9 g, Atractylodis Macrocephalae Rhizoma 10 g.  For patients with Qi-stagnancy and distending pain, add Citri Reticulatae Pericarpium 10 g, Citri Sarcodactylis Fructus 10 g, Corydalis Rhizoma 12 g, Linderae Radix 10 g. | 1 dose/ d, 2 times/ d, 120 ml/ time | Decoction | BXD has definite clinical efficacy in treating chronic gastritis. The total effective rate was 87.50%, compared with 67.50% total effective rate in control group. | [10] |

**References**

1. Bing S. Clinical Study of 80 Cases of Chronic Gastritis Treated with Banxia Xiexin Decoction. Asia-Pacific Traditional Medicine. 2015;11(24):128-9.
2. Jingjing L. Clinical Observation on Banxia Xiexin Decoction in the Treatment of Chronic Gastritis of Spleen Deficiency and Stomach Heat Type. Guangming Journal of Chinese Medicine. 2022;37(02):206-9.
3. Haitong L. Clinical Observation on the Treatment of Chronic Gastritis with Cold-Heat Complex by Banxia Xiexin Decoction. Electronic Journal of Clinical Medical Literature. 2018;5(A0):188.
4. Hong L., Ye Y., Zhiguo Z., Lijun C. Modified BanxiaxiexinTang of Chronic Gastritis Spleen Clinical Observation of Repair. Chinese Medicine Modern Distance Education of China. 2011;9(15):71-2.
5. Chuming Z. Clinical Efficacy Observation of Zhang Zhongjing Banxia Xiexin Decoction Using Sutra in the Treatment of Cold-Heat Complex Type Chronic Gastritis. Journal of Electrocardiogram (Electronic Edition). 2017;6(03):36-8.
6. Yaqing H., Meng M., Yanni L. Clinical Effect of Banxia Xiexin Decoction for Patients with Chronic Gastritis with Cold-Heat Complex. Clinical Research and Practice. 2018;3(29):128-9.
7. Weijun L. Clinical Observation on Banxia Xiexin Decoction in the Treatment of Chronic Gastritis with Spleen Deficiency and Stomach Heat Syndrome. Chinese Medicine Modern Distance Education of China. 2021;19(09):107-9.
8. Ximei W. To Study the Clinical Effect of Banxia Xiexin Decoction in the Treatment of Chronic Gastritis. Smart Healthcare. 2017;3(05):99-100.
9. Zhiyun G., Na L. Clinical Efficacy Observation of 74 Cases of Chronic Gastritis Treated with Banxia Xiexin Decoction. China Practical Medicine. 2012;7(27):170-1.
10. Huiping L. Clinical Efficacy Evaluation of Chinese Medicine Banxia Xiexin Decoction on Patients with Gastritis. Anti-infective Pharmacology. Anti-Infection Pharmacy. 2016;13(03):653-5.
11. Yan L., Shaohua W., Peng W., Jie M., Wei W. Clinical Observation on the Treatment of Chronic Gastritis with Cold-Heat Complex by Banxia Xiexin Decoction. Shenzhen Journal of Integrated Traditional Chinese and Western Medicine. 2021;31(10):80-2.
12. Huixia Z., Xiangping X., Jinghong Z. Clinical Observation of the Treatment of Chronic Gastritis Treated with Banxia Xiexin Decoction. Clinical Journal of Chinese Medicine. 2016;8(03):54-5.
13. Lixing J. Clinical Observation of Banxiaxiexin Decoction in the Treatment of Chronic Gastritis. China Continuing Medical Education. 2015;7(01):185-6.
14. Yujian W. Clinical Observation on 32 Cases of Chronic Gastritis Treated with Banxia Xiexin Decoction. Chinese Journal of Ethnomedicine and Ethnopharmacy. 2013;22 (13):88.
15. Jingyang S., Xuemin T. Clinical Observation on the Treatment of Chronic Gastritis with Banxia Xiexin Decoction. New Chinese Medicine. 2012;44(2):33-4.
16. Chunsheng G. Clinical Observation on 100 Cases of Chronic Gastritis Treated with Banxia Xiexin Decoction. Forum on Traditional Chinese Medicine. 2006;21(5):7.
17. Mei S., Xiaoping Z., Chenyu J., Guoping Z., Jianzhen S. Clinical Observation on the Treatment of Chronic Gastritis with Banxia Xiexin Decoction. Chinese Journal of Integrated Traditional and Western Medicine. 2006;26(05):463-4.
18. Yuanping S., Yuejin S., Wenzheng S. Clinical Observation on 126 Cases of Chronic Gastritis Treated with Banxia Xiexin Decoction. Medical Journal of Chinese People's Health. 1994;6(03):118-9.
19. Yuanping S., Yuejin S. Clinical Observation on the Treatment of Chronic Gastritis with Banxia Xiexin Decoction. Journal of Shandong University of Traditional Chinese Medicine. 1994;18(04):241-2.
20. Hongyan Z. Analysis of Clinical Effect and Adverse Reaction of Banxia Xiexin Decoction in the Treatment of Chronic Gastritis. Inner Mongolia Journal of Traditional Chinese Medicine. 2017;36(20):75.
21. Xiaojian G. Clinical Analysis of 46 Cases of Chronic Gastritis Treated with Banxia Xiexin Decoction. 2012;9(16):169.

**Supplementary Table 2. Representative clinical trials of BXD on HP-related diseases**

| **Patients entered** | **Study design** | **Study length** | **Sample size** | **Observation Group** | | | **Main results** | **Reference** |
| --- | --- | --- | --- | --- | --- | --- | --- | --- |
|  |  |  |  | **Medicine Detail** | **Dosage** | **Preparation** | **Details** |  |
| HP-related Positive Gastritis | Randomized | 4 weeks | 98 | Pinelliae Rhizoma 9 g, Scutellariae Radix 6 g, Zingiberis Rhizoma 6 g, Ginseng Radix et Rhizoma 6 g, Coptidis Rhizoma 3 g, Jujubae Fructus 3 g, Glycyrrhizae Radix et Rhizoma Praeparata cum Melle 6 g | 2 times/d, 100ml/t | Decoction | BXD has a clearing effect on HP and has a good effect on diseases related to HP infection. Both the BXD treatment group and the control group improved clinical symptoms in terms of treatment results and pathological results, but the difference was not statistically significant (p>0.05). The BXD treatment group was higher in HP clearance rate than the control group (p<0.01) | [1] |
| HP-related Positive Gastritis | Randomized | 1 month | 72 | Processed Pinelliae Rhizoma 12 g，Scutellariae Radix 9 g, Zingiberis Rhizoma 9 g, Codonopsis Radix 9 g, Glycyrrhizae Radix et Rhizoma Praeparata cum Melle 9 g, Coptidis Rhizoma 3 g, Jujubae Fructus 6 g. | 2 times/d | Decoction | BXD reduces gastric mucositis and has a good clearing effect on HP infection. In the treatment group, 36 cases (50%) were cured, 22 cases (31%) showed significant improvement, 9 cases (12%) improved, and 5 cases (7%) were ineffective, with a total effective rate of 93%. There is a significant difference (p<0.05) in the total effective rate compared to the control group of 68%. | [2] |
| HP-related Positive Gastritis | Randomized | 1 month | 60 | Pinelliae Rhizoma 15 g, Coptidis Rhizoma 6 g, Scutellariae Radix 10 g, Codonopsis Radix 12 g, Zingiberis Rhizoma 6 g, Glycyrrhizae Radix et Rhizoma 6 g, 3 pieces of Jujubae Fructus.  For those with severe belching, add Cyperi Rhizoma 10 g, Aucklandiae Radix 10 g, Poria 15 g, Magnoliae Officinalis Cortex 9 g, Bupleuri Radix 12 g.  For severe stomach pain, add Paeoniae Radix Alba 9 g, Toosendan Fructus 9 g, Corydalis Rhizoma 9 g.  For those with burning sensation in the stomach, add Sepiae Endoconcha 15 g, Concha Arcae 15 g, Dendrobii Caulis 12 g, Rehmannlae Radix 12 g.  For those with nausea and vomiting, add Inulae Flos 9 g, Bambusae Caulis in Taenias 12 g.  For those with obvious blood stasis, add Chuanxiong Rhizoma 12 g, Curcumae Rhizoma 9 g.  For patients with anorexia, add Jiao Sanxian (Fried Hordei Fructus Germinatus, Fried Crataegi Fructus, Fried Messa Medicata Fermentata) 15 g, Galli Gigerii Endothelium Corneum 15 g.  For patients with diarrhea, add Atractylodis Macrocephalae Rhizoma 12 g, Poria 12 g. | 1dose/d, 200ml | Decoction | BXD has therapeutic effect on chronic gastritis caused by HP. The BXD treatment group had significantly lower scores for gastric bloating, upper abdominal pain, dry mouth, anorexia, and belching and acid reflux than those in the control group. The clinical symptoms of the patients were relieved quickly, and their social relationships, psychological status, material life, and physiological functions were higher than those of the control group (P<0.05). In terms of HP clearance, the probability of HP clearance in the BXD treatment group was 90.00%, which was higher than that of the control group, and the probability of recurrence was 6.67%, which was lower than that of the control group (P<0.05). In terms of adverse drug reactions, the BXD treatment group was lower than the control group (P<0.05). The main clinical symptoms were nausea and constipation, and no serious consequences occurred. | [3] |
| HP-related halitosis | Randomized | 10 days | 90 | Pinelliae Rhizoma Praeparatum 9 g, Scutellariae Radix 9 g, Coptidis Rhizoma 6 g, Zingiberis Rhizoma 6 g, Codonopsis Radix 9 g, Glycyrrhizae Radix et Rhizoma 6 g，3 pieces of Jujubae Fructus.  For patients who suffer from spleen and stomach damp-heat, add Atractylodis Rhizoma 9g and Taraxacai Herba 15g. | 2 times/d | Decoction | BXD plays a synergistic therapeutic effect on HP-related halitosis. The total effective rates of short-term and long-term effects on halitosis of the BXD study group were higher than those of the control group (p<0.05). | [4] |
| HP-related Positive Gastritis | Randomized | 2 weeks | 90 | Pinelliae Rhizoma Praeparatum 13 g, Codonopsis Radix 12 g, Scutellariae Radix 12 g, Coptidis Rhizoma 6 g, Zingiberis Rhizoma 6 g, Glycyrrhizae Radix et Rhizoma 7 g, 3 pieces of Jujubae Fructus.  For those with unformed stools or spleen deficiency and dampness can add Atractylodis Macrocephalae Rhizoma 14 g, Poria 20 g. | 1 does/d, 200ml | Decoction | BXD can effectively improve the treatment effect of Hp-related gastritis and reduce the rate of adverse reactions. In terms of TCM syndrome scores: the TCM syndrome score of the BXD treatment group was significantly lower than that of the control group, and the difference was statistically significant (P <0.05). In terms of clinical efficacy: BXD treatment group was higher than that in the control group, and the difference was statistically significant (P < 0.05). There was no statistically significant difference in the adverse reaction rate between the observation group and the control group after treatment (P > 0.05). | [5] |
| HP-related Positive Gastritis | Randomized | 2 weeks | 110 | Pinelliae Rhizoma Praeparatum 10 g，Coptidis Rhizoma 6 g，Scutellariae Radix 12 g，Zingiberis Rhizoma 12 g，Codonopsis Radix 12 g, Glycyrrhizae Radix et Rhizoma 10 g，Jujubae Fructus 15 g. | 2 times/d | Decoction | BXD has significant efficacy in patients with chronic gastritis and HP-positive patients. The BXD treatment group had lower TCM symptom scores of gastric bloating, epigastric pain, belching, sore throat, lack of appetite and indigestion after treatment (P < 0.05). The levels of hs-CRP and IL-6 in the experimental group were lower after treatment (P <0.05). Compared with the HP clearance rate in the control group, which was 80.00% (44/55), the HP clearance rate in the test group was 96.36% (53/55), which was higher, and the difference was statistically significant (P < 0.05). | [6] |
| Helicobacter pylori (Hp)-re lated peptic ulcers (PUs) | Randomized | 7 d | 60 | Rhizoma Pinelliae 10 g, Rhizoma Zingiberis 10 g, Rhizoma Coptidis 5 g, Radix Scutellariae Baicalensis 10 g, Radix Ginseng 10 g, Fructus Jujubae 10 g, Radix Glycyrrhizae 10 g | 2 times/d | formula granules | BXD regulates the TGF-β/Smad signal ing pathway by inhibiting the expression of TGF-β1 and Smad3, and increasing the expression of Smad7 | [7] |

**References**

1. Zhiwei Q., Mingjun Y., Chunlei L. Clinical Study on 98 Cases of Banxiaxiexin Decoction for *H. Pylori*-Related Positive Gastritis. Journal of Beihua University (Natural Science). 2017;18(03):368-70.
2. Kefu C., Buju H. Clinical Observation on 72 Cases of Banxiaxiexin Decoction for Pylori-Related Positive Gastritis. New Chinese Medicine. 1994;(03):20-1.
3. Chunguan L. Clinical Effect and Effective Effect Analysis of Banxia Xiexin Decoction in the Treatment of Helicobacter Pylori-positive Chronic Gastritis. China & Foreign Medical Treatment. 2019;38(21):155-157-63.
4. Shimei S. Clinical Observation on the Treatment of *Helicobacter Pylori*-associated Halitosis with Banxia xiexin Decoction. Inner Mongolia Journal of Traditional Chinese Medicine. 2014;33(31):33-4.
5. Lijuan Y. Clinical Observation on Banxia Xiexin Decoction in Treating HP-related Gastritis. Chinese Medicine Modern Distance Education of China. 2022;20(03):92-3.
6. Yue C. Clinical Observation on Banxia Xiexin Decoction in the Treatment of Chronic Gastritis with *Helicobacter Pylori* Positive. Chinese Medicine Modern Distance Education of China. 2023;21(07):99-101.
7. Chen S., Huang Y., Wan S., Huang Y., Liang H., Chen S. Effect of Banxia Xiexin Decoction on *Helicobacter Pylori*-Related Peptic Ulcers and Its Possible Mechanism via the TGF-*β*/Smad Signaling Pathway. Journal of traditional Chinese medicine. 2018;38(3): 419-26.

**Supplementary Table 3. Representative clinical studies of BXD on chronic atrophic gastritis.**

| **Patients entered** | **Study design** | **Study length** | **Sample size** | **Observation Group** | | | **Main results** | **Reference** |
| --- | --- | --- | --- | --- | --- | --- | --- | --- |
|  |  |  |  | **Medicine Detail** | **Dosage** | **Preparation** | **Details** |  |
| chronic atrophic gastritis | Randomized | 3 months | 78 | Pinelliae Rhizoma Praeparatum cum Zingibere et Alumine 12 g, Codonopsis Radix 12 g and Scutellariae Radix 12 g, Glycyrrhizae Radix et Rhizoma 9 g, Jujubae Fructus 9 g, Coptidis Rhizoma 6 g, and Zingiberis Rhizoma 6 g, Herba Hedyotidis 30 g, Scutellariae Barbatae Herba 15 g.  For patients with obvious epigastric pain, add Corydalis Rhizoma 9 g, and Trogopterus Dung 9 g.  For those with severe bloating, add Aucklandiae Radix 9 g, and Magnoliae Officinalis Cortex 9 g.  For those with severe waterbrash and heartburn, add Sepiae Endoconcha 30 g and Arcae Concha 30 g. | 1 dose/ d, 2 times/ d, 200ml/ time | Decoction | The use of BXD in the treatment of CAG can improve patients' clinical symptoms and reduce gastric mucosal atrophy. The experiment evaluated individual clinical symptom scores, total clinical symptom scores, pathology scores, HP clearance rate, and gastric function levels. All five indicators showed that the BXD treatment group had a good effect on CAG, and there was a statistically significant difference in the treatment effect compared with the control group. | [1] |
| chronic atrophic gastritis | Randomized | 8 weeks | 60 | Pinelliae Rhizoma Praeparatum cum Zingibere et Alumine 12 g, Ginseng Radix et Rhizoma 9 g, Scutellariae Radix 9 g, Zingiberis Rhizoma 9 g, Glycyrrhizae Radix et Rhizoma Praeparata cum Melle 9 g, Coptidis Rhizoma 3 g, 4 pieces of Jujubae Fructus.  For patients with stagnant movement of Qi and blood, add Aurantii Fructus 6 g, Citri Reticulatae Pericarpium 6 g.  For those with severe heat syndrome, replaced Zingiberis Rhizoma with Zingiberis Rhizoma Recens , add Forsythiae Fructus 10 g, Gentianae Radix et Rhizoma 5 g.  For patients with constipation, add Processed Rhei Radix et Rhizoma 6 g. | 1 dose/ d, 2 times/ d | Decoction | BXD has good clinical efficacy and high safety in treating CAG patients. The patients in the control group and the observation group were improved in TCM syndrome score, gastroscopy and pathological effect, and the treatment effect of the observation group was better than that of the control group. The Hp clearance rate was 95.45% in the observation group and 89.47% in the control group, and the difference was not statistically significant (P>0.05). | [2] |
| chronic atrophic gastritis | Randomized | 2 weeks | 60 | Pinelliae Rhizoma Praeparatum cum Zingibere et Alumine 10 g, Scutellariae Radix 10 g, Coptidis Rhizoma 6 g, Zingiberis Rhizoma and Codonopsis Radix 15 g, Glycyrrhizae Radix et Rhizoma 6 g, Jujubae Fructus 10 g.  For patients with spleen deficiency with dampness, add Poria 15 g, Coicis Semen 20 g.  For patients with stagnation of Qi and blood, add Salviae Miltiorrhizae Radix et Rhizoma 15 g, Trogopterus Dung 15 g.  For those with severe pain, add Toosendan Fructus 15 g, Corydalis Rhizoma 10 g.  For those with severe anorexia, add Galli Gigerii Endothelium Corneum 6 g. | 1 dose/ d, 2 times/ d | Decoction | BXD treatment of chronic atrophic gastritis can significantly alleviate clinical symptoms, improve the inflammatory state of gastric mucosa, increase the number of gastric glands, and reverse precancerous lesions to a certain extent. | [3] |
| chronic atrophic gastritis | Randomized | 3 months | 80 | Pinelliae Rhizoma praeparatum 12 g, Zingiberis Rhizoma 10 g, Scutellariae Radix 10 g, Coptidis Rhizoma 6 g, Codonopsis Radix 15 g, Glycyrrhizae Radix et Rhizoma Praeparata cum Melle 6 g, Jujubae Fructus 15 g.  For patients with Qi stagnation, add Citri Sarcodactylis Fructus and Aucklandiae Radix.  For those with epigastric pain, add Corydalis Rhizoma and Paeoniae Radix Alba.  For those with severe dampness, add Atractylodis Rhizoma.  For patients with blood stasis, add Persicae Semen and Notoginseng Radix et Rhizoma. | 1 dose/ d, 2 times/ d, 150ml/ time | Decoction | BXD has good effect in treating chronic atrophic gastritis. Comparing the BXD treatment group with the control group, the symptom efficacy and gastroscopy efficacy of the treatment group were better than those of the control group (p<0.05). The reduction of gastric mucosal atrophy, reduction of intestinal metaplasia and improvement of dysplasia in the treatment group were better than those in the control group. The HP infection negative conversion effect in the treatment group was better than that in the control group. | [4] |
| chronic atrophic gastritis | Randomized | 3 months | 72 | Processed Pinelliae Rhizoma 15 g, Stir-fried Scutellariae Radix 10 g, Coptidis Rhizoma 5 g, Zingiberis Rhizoma 9 g, Codonopsis Radix 15 g, Glycyrrhizae Radix et Rhizoma Praeparata cum Melle 6 g, 5 pieces of Jujubae Fructus.  For patients with pain, add Paeoniae Radix Alba and Corydalis Rhizoma.  For those who vomit acid, add Sepiae Endoconcha.  For severe blood stasis, add Salviae Miltiorrhizae Radix et Rhizoma and Angelicae Sinensis Radix.  For patients with anorexia, add Stir-fried Hordei Fructus Germinatus and Scorched Crataegi Fructus.  For patients with stagnation of Qi of the liver and stomach, add Stir-fried Bupleuri Radix, Processed Cyperi Rhizoma and Perillae Caulis.  For those with severe spleen deficiency, add Atractylodis and Astragali Radix. | 1 dose/ d, 2 times/ d, 200ml/ time | Decoction | BXD has good efficacy in treating chronic atrophic gastritis and can inhibit HP.  The scores of various symptoms in the BXD treatment group decreased after treatment, with significant statistical differences compared with that score before treatment. In terms of changes in HP infection after treatment, the negative conversion rate in the treatment group was 66.7%, and the negative conversion rate in the control group was 37.5%. There was a significant difference between the two (p<0.05). | [5] |
| chronic atrophic gastritis | Randomized | 3 months | 60 | 4 pieces of Jujubae Fructus, Coptidis Rhizoma 3 g, Zingiberis Rhizoma 9 g, Glycyrrhizae Radix et Rhizoma Praeparata cum Melle 9 g, Codonopsis Radix 9 g, Scutellariae Radix 9 g, Pinelliae Rhizoma 15 g.  For those with severe abdominal pain and distension, add Magnoliae Officinalis Cortex 9 g, Citri Sarcodactylis Fructus 9 g.  For those with severe acid reflux and belching, add Sepiae Endoconcha 20 g.  For those with severe blood stasis, add Salviae Miltiorrhizae Radix et Rhizoma 10 g. | 1 dose/ d, 2 times/ d, 200ml/ time | Decoction | BXD can be used in the clinical treatment of chronic atrophic gastritis, for BXD can improve the pathological state of gastric mucosa. Experiments show that the total effective rate of the BXD treatment group (93.33%) is higher than that of the control group (70.00%). The symptom scores after treatment were lower than those in the control group (P<0.05). After treatment, the gastric mucosal pathology score was lower than that of the control group (p<0.05). | [6] |
| chronic atrophic gastritis | Randomized | 2 months | 86 | Pinelliae Rhizoma 15 g, Scutellariae Radix 9 g, Zingiberis Rhizoma 9 g, Ginseng Radix et Rhizoma 9 g, and Glycyrrhizae Radix et Rhizoma Praeparata cum Melle 9 g, Coptidis Rhizoma 3 g, 4 pieces of Jujubae Fructus.  For patients with severe dampness and heat, subtract Zingiberis Rhizoma, add Scutellariae Radix to 15 g.  For those with severe epigastric pain, add Paeoniae Radix Alba 20 g, Corydalis Rhizoma 10 g.  For patients with stagnation of Qi of the liver and stomach, add Cyperi Rhizoma 10 g, Perillae Caulis 8 g, Platycodonis Radix 8 g.  For patients with constipation, add Rhei Radix et Rhizoma 6 g.  For those with severe regurgitation of gastric acid, add Fritillariae Cirrhosae Bulbus 10 g, Sepiae Endoconcha 10 g. | 1 dose/ d, 2 times/ d, 150ml/ time | Decoction | BXD could repaired the gastric mucosa in chronic atrophic gastritis. The proportion of patients with gastric mucosal inflammation disappeared and the area of gastric mucosal atrophy decreased by more than 2/ 3 in observation group was significantly higher than that in control group, with statistical significance (P<0.05). The epigastric pain, heartburn, acid reflux, sodium sodium symptom scores and incidence of adverse reactions in observation group were lower than those in control group, with statistical significance (P<0.05). | [7] |
| chronic atrophic gastritis | Randomized | 30 days | 80 | Codonopsis Radix 20 g, Scutellariae Radix 10 g, Herba Hedyotidis 30 g, Taraxaci Herba 20 g, Glycyrrhizae Radix et Rhizoma6 g, Zingiberis Rhizoma 5 g, Coptidis Rhizoma 6 g, Aurantii Fructus Immaturus 10 g, Pinelliae Rhizoma praeparatum 10 g. | 1 dose/ d, 2 times/ d, 100 ml/ time | Decoction | BXD has a significant effect in treating chronic atrophic gastritis, which can improve patients' clinical symptoms and increase the number of cases of Hp infection turning negative. The Hp clearance rate was 87.50% in the observation group and 62.50% in the control group, with statistically significant (P<0.05). | [8] |
| chronic atrophic gastritis | Randomized | 1 month | 106 | Pinelliae Rhizoma 15 g, Scutellariae Radix, Zingiberis Rhizoma, Ginseng Radix et Rhizoma and Glycyrrhizae Radix et Rhizoma Praeparata cum Melle 9 g each, Coptidis Rhizoma 3 g, 4 pieces of Jujubae Fructus.  For patients with epigastric fullness syndrome caused by abnormal movement of the spleen and stomach, subtract Zingiberis Rhizoma to 2 g, add Zingiberis Rhizoma Recens 4 g.  For patients with epigastric fullness syndrome caused by stomach-Qi deficiency and coldheat complex, increase the amount of Glycyrrhizae Radix et Rhizoma Praeparata cum Melle. | 1 dose/ d, 2 times/ d | Decoction | BXD is effective in treating chronic atrophic gastritis with high safety and few complications. The Hp clearance rate was 81.1% in the observation group and 50.0% in the control group. The total effective rate was 94.3%, compared with 81.1% total effective rate in control group. The incidence of adverse reactions in the treatment group was significantly lower than that in the control group, and the difference was statistically significant (P<0.05). | [9] |
| chronic atrophic gastritis | Randomized | 40 days | 120 | Pinelliae Rhizoma 9 g, Scutellariae Radix 10 g, Coptidis Rhizoma 10 g, Codonopsis Radix 10 g, Zingiberis Rhizoma 6 g, Glycyrrhizae Radix et Rhizoma Praeparata cum Melle 6 g;  For patients with epigastric pain, add Corydalis Rhizoma and Notoginseng Radix et Rhizoma.  Forpatients with abdominal distension, add Citri Sarcodactylis Fructus and Amomi Fructus.For parients with acid reflux, add Arcae Concha and Bletillae Rhizoma.  For patients with epigastric distension, add Raphani Semen and Haematitum.  For parients who feeling of oppression over the chest, add Curcumae Radix and Ziziphi Spinosae Seme. | 1 dose/ d, 2 times/ d, 150 ml/ time | Decoction | BXD has significant clinical effect in treating chronic atrophic gastritis and can significantly improve HP clearance rate. The total effective rate was 94.8%, compared with 77.2% total effective rate in control group. The Hp clearance rate was 70.9% in the observation group and 52.9% in the control group. | [10] |

**References**

1. Qian L. Clinical Study on Banxia Xiexin Decoction in the Treatment of Chronic Atrophic Gastritis with Spleen-stomach Dampness Syndrome. Guangming Journal of Chinese Medicine. 2022;37(20):3736-8.
2. Yujia C. Clinical Study on Banxia Xiexin Decoction in the Treatment of Chronic Atrophic Gastritis with Spleen-stomach Dampness Syndrome. Guangming Journal of Chinese Medicine. 2021;36(07):1102-5.
3. Siyin L. Clinical Study on Pinellia Heart-Draining Decoction for Treating Chronic Atrophic Gastritis. Henan Traditional Chinese Medicine. 2015;35(01):26-7.
4. Yujin W., Wen J., Yang J. Clinical Study on Banxia Xiexin Decoction for Treating Chronic Atrophic Gastritis. China Practical Medicine. 2009;4(12):175-7.
5. Chengye Y. Clinical Study on Banxia Xiexin Decoction for Treating Chronic Atrophic Gastritis. Liaoning Journal of Traditional Chinese Medicine. 2007;34(11):1583-4.
6. Jianhua G., Zena Z., Jing Z., Yanqun Y., Ziwei W., Xiaohu Z. Clinical Study on Banxia Xiexin Decoction for Treating Chronic Atrophic Gastritis. Inner Mongolia Journal of Traditional Chinese Medicine. 2021;40(03):3-4.
7. Laishun G. Clinical Effect of Banxia Xiexin Decoction on Chronic Atrophic Gastritis. Shenzhen Journal of Integrated Traditional Chinese and Western Medicine. 2019;29(10):53-4.
8. Lin Z., Pengtao X. Clinical Effect Analysis of Banxia Xiexin Decoction in the Treatment of Chronic Atrophic Gastritis. China Continuing Medical Education. 2018;10(09):122-4.
9. Liping B. Clinical Analysis of Banxia Xiexin Decoction in the Treatment of Chronic Atrophic Gastritis. Journal of Snake. 2015;27(03):265-6.
10. Dayun Z., Deqin B. Clinical Effect of Banxia Xiexin Decoction in the Treatment of Chronic Atrophic Gastritis. World Latest Medicine Information. 2016;16(47):193-4.
11. Wei Q. Clinical Observation of Banxia Xiexin Decoction in Treating 42 cases of Chronic Atrophic Gastritis. Clinical Journal of Chinese Medicine. 2016;8(09):84-5.
12. Xiangjun K., Jingguang X., Qinghui L., Shuhua Z. Clinical Observation of Banxia Xiexin Decoction in Treating 20 cases of Chronic Atrophic Gastritis. Modern Medicine Journal of China. 2011;13(10):84-5.
13. Mingde C. Clinical Research of Pinelliae Decoction for PurgingStomach-Fire to Healing Chronic Atrophic Gastritis. Tianjin University of Traditional Chinese Medicine. 2007:257-9.
14. Xuebing L., Liang L. Clinical Observation of Banxia Xiexin Decoction for Treating Chronic Atrophic Gastritis. Journal of Liaoning University of Traditional Chinese Medicine. 2000;2(02):124-5.
15. Zhigang Z. Clinical Observation of Banxia Xiexin Decoction for Treating Chronic Atrophic Gastritis. Journal of Electrocardiogram. 2018;7(02):254-6.
16. Quanjun Y. Clinical Observation of Banxia Xiexin Decoction for Treating Chronic Atrophic Gastritis. World Latest Medicine Information. 2017;17(02):154.
17. Qiming W. Clinical Effect Observation of Banxia Xiexin Decoction in Treating 40 cases of Chronic Atrophic Gastritis. Inner Mongolia Journal of Traditional Chinese Medicine. 2016;35(17):30.

**Supplementary Table 4. Representative clinical trials of BXD on superficial gastritis**

| **Patients entered** | **Trial design** | **Trial length** | **Sample size** | **Observation Group** | | | **Main results** | **Reference** |
| --- | --- | --- | --- | --- | --- | --- | --- | --- |
|  |  |  |  | **Medicine Detail** | **Dosage** | **Preparation** | **Details** |  |
| Superficial gastritis | Randomized | 2 months | 136 | Pinelliae Rhizoma10 g, Codonopsis Radix 9 g, Scutellariae Radix 9 g, Glycyrrhizae Radix et Rhizoma Praeparata cum Melle 6 g, Zingiberis Rhizoma 6 g, Coptidis Rhizoma 6 g, 4 pieces of Jujubae Fructus.  For those with severe heat, the amount of Codonopsis Radix was reduced and 5 g of Coptidis Rhizoma and Scutellariae Radix were added.  For those with severe cold, Increase the dosage of Zingiberis Rhizoma, or 5 g of Euodiae Fructus and 5 g of Caryophylli Flos were added.  For those with severe dampness and heat, 5 g of Pogostemonis Herba and Artemisiae Scopariae Herba were added.  For those with Qi stagnation, 10 g of Cyperi Rhizoma and Aucklandiae Radix were added.  For those with acid reflux, 10 g of Arcae Concha and Sepiae Endoconcha were added. | 1 dose/ d,  150ml/ t,  2 times/ d. | Decoction | BXD is effective in treating superficial gastritis, can effectively improve symptoms, radically cure HP, and has a low recurrence rate. The experiment compared gastrin, motilin, and somatostatin before and after treatment, and observed the eradication of HP. After 6 months of follow-up, the recurrence rate was 6.06% in the observation group and 44.44% in the control group. | [1] |
| Chronic superficial gastritis | Randomized | 4 weeks | 100 | Pinelliae Rhizoma 20 g, Codonopsis Radix 15 g, Jujubae Fructus 10 g, Zingiberis Rhizoma 9 g, Coptidis Rhizoma 6 g, Glycyrrhizae Radix et Rhizoma Praeparata cum Melle 6 g, Scutellariae Radix 6 g  For patients with burning stomach, add Coptidis Rhizoma to 9 g, add Taraxaci Herba 20 g.  For patients with spleen and stomach deficiency, add Codonopsis Radix to 30 g, add Astragali Radix 20 g, Atractylodis Macrocephalae Rhizoma 15 g, Euodiae Fructus 6 g.  For patients with acid reflux, add Sepiae Endoconcha 30 g, Arcae Concha 18 g.  For patients with epigastric pain, add Corydalis Rhizoma 12 g, Paeoniae Radix Alba 10 g, Linderae Radix 10 g.  For patients with anorexia, add Hordei Fructus Germinatus 15 g, Setariae Fructus Germinatus 15 g, Amomi Fructus 6 g.  For patients with Qi stagnation, add Citri Sarcodactylis Fructus 10 g, Magnoliae Officinalis Cortex 10 g, Aucklandiae Radix 6 g and Aurantii Fructus 6 g. | 1 dose/ d,  2 times/ d. | Decoction | BXD can achieve good curative effect in the treatment of chronic superficial gastritis. The total clinical effective rate of observation group was 94.0%, which was higher than that of control group (70.0%). Comparison of TCM symptom score: After treatment, the improvement of TCM symptom score in observation group was better than that in control group. | [2] |
| Chronic superficial gastritis | Randomized | 4 weeks | 62 | Pinelliae Rhizoma 20 g，Zingiberis Rhizoma 9 g，Scutellariae Radix 6 g，Coptidis Rhizoma 6 g，Codonopsis Radix 15 g，Jujubae Fructus 10 g，Glycyrrhizae Radix et Rhizoma Praeparata cum Melle 6 g.  For those with burning stomach: add Coptidis Rhizoma to 9 g, add Taraxacai Herba 20g.  For those with severe pain: add Corydalis Rhizoma 12 g, Linderae Radix 10g, Paeoniae Radix Alba 10g.  For those with acid reflux: add Sepiae Endoconcha 30g, Concha Arcae 18 g.  For those with Qi stagnation: add Magnoliae Officinalis Cortex 10g, Aurantii Fructus 6g, Aucklandiae Radix 6g, Citri Sarcodactylis Fructus 10g.  For those with anorexia: add Amomi Fructus 6g, Setariae Fructus Germinatus 15g, Hordei Fructus Germinatus 15g | 1 dose/ d,  2 times/ d. | Decoction | BXD treatment achieves good efficacy in treating chronic gastritis. In the treatment group, the total effective rate was 93.7%. In the control group, the total effective rate was 76.7%. | [3] |

**References**

1. Honghong L., Li W. Observation on Banxia Xiexin Decoction in Treating Superficial Gastritis. Journal of Practical Traditional Chinese Medicine. 2019;35(04):394-5.
2. Liuhua Y. Clinical Observation on 50 Cases of Banxia Xiexin Decoction for Superficial Gastritis. China's Naturopathy. 2017;25(12):39-40.
3. Qiuling Z. Clinical Observation on 32 Cases of Banxia Xiexin Decoction for Superficial Gastritis. China & Foreign Medical Treatment. 2010;29(07):127.
4. Xiying C. Observation on Banxia Xiexin Decoction in Treating Chronic Stomach Disease. Guangming Journal of Chinese Medicine. 2017;32(23):3418-20.

**Supplementary Table 5.** Representative clinical studies of BXD on ulcerative diseases (peptic ulcer and ulcerative colitis)

| **Patients entered** | **Study design** | **Study length** | **Sample size** | **Observation Group** | | | **Main results** | **Reference** |
| --- | --- | --- | --- | --- | --- | --- | --- | --- |
|  |  |  |  | **Medicine Detail** | **Dosage** | **Preparation** | **Details** |  |
| Ulcerative colitis | Randomized | 8 weeks | 80 | Scutellariae Radix 10 g, Zingiberis Rhizoma 5 g, Coptidis Rhizoma 5 g, Codonopsis Radix 20 g, Processed Pinelliae Rhizoma 10 g, Processed Rhei Radix et Rhizoma 5 g, Glycyrrhizae Radix et Rhizoma Praeparata cum Melle10 g.  For those with abdominal pain, add Paeoniae Radix Alba 5 g, Angelicae Sinensis Radix 5 g, Aucklandiae Radix 5 g.  For those with severe diarrhea, add Portulacae Herba 5 g and Fried Coicis Semen 5 g.  For those with persistent diarrhea, add Puerariae Lobatae Radix 5 g, Cimicifugae Rhizoma 5 g, Astragali Radix 10 g.  For those with indigestion, add Hordei Fructus Germinatus 5 g, Messa Medicata Fermentata 5 g, Fried Crataegi Fructus 5 g. | 2 times/ d | Decoction | BXD relieved the symptom of ulcerative colitis, with the total effective rate was 95.00%, compared with 60.00% total effective rate in control group. | [1] |
| Ulcerative colitis | Randomized | 1 month | 118 | Jujubae Fructus 20 g, Coptidis Rhizoma 5 g, Zingiberis Rhizoma 10 g, Ginseng Radix et Rhizoma 20 g, Pinelliae Rhizoma Praeparatum cum Zingibere et Alumine 9 g, Glycyrrhizae Radix et Rhizoma Praeparata cum Melle 10 g, Scutellariae Radix 5 g.  For those with abdominal pain, add Angelicae Sinensis Radix 10 g, Aucklandiae Radix10 g, Paeoniae Radix Alba10 g.  For those with qi stagnation, add Aucklandiae Radix 13 g and Citri Reticulatae Pericarpium 13 g. | 2 times/ d | Decoction | BXD therapy group has better performance in terms of the number of intestinal flora colonies,  Pro-inflammatory cytokines, and stool markers and Baron endoscopic score | [2] |
| Peptic ulcer | Randomized | 4 weeks | 94 | On the basis of the control group, BXD was added.  Pinelliae Rhizoma 12 g, Ginseng Radix et Rhizoma 9 g, Scutellariae Radix 9 g, Zingiberis Rhizoma 9 g, 12 pieces of Jujubae Fructus, Coptidis Rhizoma 3 g, Cinnamomi Ramulus 9 g, Angelicae Sinensis Radix 10 g, Corydalis Rhizoma 12 g, Salviae Miltiorrhizae Radix et Rhizoma 15 g, Glycyrrhizae Radix et Rhizoma Praeparata cum Melle 9 g.  For those with dry mouth and bitter taste, remove Cinnamomi Ramulus and add Taraxacai Herba 15 g.  For those with abdominal distention, add Aurantii Fructus 10 g.  For those with chest tightness and belching, add Bupleuri Radix 10g and Cyperi Rhizoma 10 g.  For those with acid reflux, add Concha Arcae15 g and Sepiae Endoconcha 15 g.  For constipation, add Cannabis Fructus 30 g, Rhei Radix et Rhizoma 10 to 15 g.  For those with anorexia, add Stir-fired Hordei Fructus Germinatus 10 g, Stir-fried Crataegi Fructus 10 g, Fried Messa Medicata Fermentata 10 g.  For patients with diarrhea add Poria 30 g. | 2 times/ d | Decoction | The scores of belching and acid reflux in the control group were significantly lower than before treatment. The scores of belching and acid reflux, epigastric pain, and fear of cold limbs in the observation group were all lower than before treatment. | [3] |
| Peptic ulcer | Randomized | 4 weeks | 78 | Pinelliae Rhizoma 15 g, Coptidis Rhizoma 8 g, Scutellariae Radix 10 g, Powder of Notoginseng Radix et Rhizoma 6 g，Zingiberis Rhizoma 8 g, Codonopsis Radix 10 g, Powder of Sepiae Endoconcha 10 g, Glycyrrhizae Radix et Rhizoma Praeparata cum Melle 10 g, Amomi Fructus 6 g, 6 pieces of Jujubae Fructus.  For those with gastric bleeding, add Bletillae Rhizoma 15g, Sepiae Endoconcha 15 g. | 2 times/ d, 200 mL/ time | Decoction | BXD relieved the symptom of peptic ulcer. The total effective rate of treatment in the observation group was 94.9%, and the incidence of adverse reactions was 5.1%. | [4] |
| Peptic ulcer | Randomized | 4 weeks | 120 | Pinelliae Rhizoma Praeparatum 15 g, Scutellariae Radix 10 g, Coptidis Rhizoma 8 g, Zingiberis Rhizoma 8g, Powder of Notoginseng Radix et Rhizoma 6 g, Powder of Sepiae Endoconcha 10 g, Codonopsis Radix 10 g, Glycyrrhizae Radix et Rhizoma Praeparata cum Melle 10 g, 6 pieces of Jujubae Fructus, Amomi Fructus 6 g.  For those with gastric bleeding, add Bletillae Rhizoma 15 g, Sepiae Endoconcha 15 g. | 2 times/ d | Decoction | BXD relieved the symptom of peptic ulcer. Among the 60 cases in the treatment group, 34 cases were cured, 22 cases improved, and 4 cases were ineffective, with a total effective rate of 92%. | [5] |
| Peptic ulcer | Randomized | 4 weeks | 60 | Pinelliae Rhizoma 15 g, Scutellariae Radix 10 g, Coptidis Rhizoma 8 g, Zingiberis Rhizoma 8 g, Powder of Notoginseng Radix et Rhizoma 6 g, Powder of Sepiae Endoconcha 10 g, Codonopsis Radix 10 g, Glycyrrhizae Radix et Rhizoma Praeparata cum Melle 10 g, 6 pieces of Jujubae Fructus, Amomi Fructus 6 g.  For those with gastric bleeding, add Bletillae Rhizoma 15 g, Sepiae Endoconcha 15 g. | 2 times/ d, 200 mL/ time | Decoction | BXD relieved the symptom of peptic ulcer. In the treatment group, 21 cases were cured, 8 cases improved, and 1 case was ineffective. The total effective rate was 92%. After statistical processing, the difference between the two groups was statistically significant (P＜0.01). | [6] |
| Peptic ulcer | Randomized | 3 weeks | 108 | Scutellariae Radix 9 g, Glycyrrhizae Radix et Rhizoma Praeparata cum Melle 9 g, Zingiberis Rhizoma 9 g, Ginseng Radix et Rhizoma 9 g, 5 pieces of Jujubae Fructus，Coptidis Rhizoma 5 g，Pinelliae Rhizoma 15 g. | 2 times/ d, 150 mL/ time | Decoction | BXD relieved the symptom of peptic ulcer. The total effective rate of the observation group was 96.29% and the control group was 79.63%. The difference between the two groups was statistically significant (P<0.05). After treatment, the IL-2 levels in both groups were higher than before treatment, and the IL-6 levels were lower than before treatment. | [7] |
| Peptic ulcer | Randomized | 1 month | 206 | Paeoniae Radix Alba 30 g, Pinelliae Rhizoma 12 g, Zingiberis Rhizoma 12 g, 12 pieces of Jujubae Fructus, Coptidis Rhizoma 6 g, Scutellariae Radix 12 g, Codonopsis Radix 30 g, Glycyrrhizae Radix et Rhizoma 9 g.  For those with ecchymosis on the tongue, add Notoginseng Radix et Rhizoma 10 g or Persicae Semen 10 g.  For patients with epigastric burning and pain, add Bambusae Caulis in Taenias 15 g and Taraxacai Herba 30 g.  For constipation, add Coicis Semen 30 g and Cannabis Fructus 30 g.  For those with cold and painful epigastric pain, add Zingiberis Rhizoma 10 g and Aspongopus 10 g.  For those with nausea and vomiting sour water, add Concha Arcae 15 g and Sepiae Endoconcha 15 g.  For those with anorexia, add Messa Medicata Fermentata 15 g, Crataegi Fructus 15 g.  For those with abdominal distension, add Fried Raphani Semen 15 g, Magnoliae Officinalis Cortex 15 g.  If those whose tongue is red and the coating is yellow and greasy, add 20 g of Artemisiae Scopariae Herba and 30 g of Talcum. | 2 times/ d | Decoction | The total effective rate of treatment in the research group was higher than that in the control group. After treatment, the SAS and SDS scores of both groups were reduced, and the study group was lower than the control group. The study group's role function, cognitive function, social function and physical function were higher than those of the control group | [8] |
| Peptic ulcer | Randomized | 4 weeks | 380 | Pinelliae Rhizoma 13 g, Scutellariae Radix 8 g, Coptidis Rhizoma 7 g, Zingiberis Rhizoma 6 g, Powder of Notoginseng Radix et Rhizoma 6g, Powder of Sepiae Endoconcha 10 g, Codonopsis Radix 10 g, Glycyrrhizae Radix et Rhizoma Praeparata cum Melle 10 g, 6 pieces of Jujubae Fructus, Amomi Fructus 6 g.  For those with gastric bleeding, add 15 g of fried Bletillae Rhizoma and 15 g of Sepiae Endoconcha. | 2 times/ d, 200 mL/ time | Decoction | BXD relieved the symptom of peptic ulcer. Among the 190 cases in the treatment group, 125 cases were cured, 56 cases were improved, and 9 cases were ineffective. The total effective rate was 96%, which was significantly different from the control group (p<0.01). | [9] |
| Peptic ulcer | Randomized | 4 weeks | 489 | Pinelliae Rhizoma 12 g，Coptidis Rhizoma 6 g，Scutellariae Radix 10 g，Codonopsis Radix 15 g, Zingiberis Rhizoma 6 g，Citri Reticulatae Pericarpium 10 g，Glycyrrhizae Radix et Rhizoma Praeparata cum Melle 6 g. | 2 times/ d, 100 mL/ time | Decoction | BXD relived the symptom of peptic ulcer. In the treatment group, 184 cases were cured, 52 cases improved, and 13 cases were ineffective. The total effective rate reached 94.77%. Compared with the total effective rate of 70.41% in the control group, there was a statistically significant difference (p<0.01). | [10] |

**References**

1. Haiyan Z. Observation on the Therapeutic Effect of Banxiaxiexin Decoction in the Treatment of 40 Cases of Ulcerative Colitis. Asia-Pacific Traditional Medicine. 2016;12(02):109-110.
2. Zhenxing W., Jianguo L., Jindu Z. Clinical Observation on the Effect of Banxia Xiexin Decoction in the Treatment of Patients with Ulcerative Colitis on Anti-inflammation and Intestinal Flora Regulation. Guangming Journal of Chinese Medicine. 2022;37(13):2289-92.
3. Lin D. Clinical Observation of Peptic Ulcer Treated with Banxia Xiexin Decoction. Medical Journal of Chinese People's Health. 2018;30(13):73-5.
4. Bailin L. Clinical Observation of Peptic Ulcer Treated with Banxia Xiexin Decoction. Journal of China Prescription Drug. 2014;12(04):124.
5. Hongran W. Clinical Observation of Peptic Ulcer Treated with Banxia Xiexin Decoction. Guangming Journal of Chinese Medicine. 2011;26(08):1576-7.
6. Peixiang W. Clinical Observation of Peptic Ulcer Treated with Banxia Xiexin Decoction. China Practical Medicine. 2010;5(36):156.
7. Yang S., Fengyu Z., Jiao L. Clinical Observation of Peptic Ulcer Treated with Banxia Xiexin Decoction. China Practical Medicine. 2021;37(07):1104-5.
8. Ping L., Yang L. Clinical Effect of Banxia Xiexin Decoction on Peptic Ulcer. Clinical Research and Practice. 2020;5(03):155-6.
9. Yazhou F. Clinical Analysis on the Treatment of Peptic Ulcer by Banxia Xiexin Decoction. Guangming Journal of Chinese Medicine. 2011;26(01):97-8.
10. Feng L. Clinical Effect Analysis on the Treatment of Peptic Ulcer by Banxia Xiexin Decoction. China Medical Herald. 2010;7(19):102-3.

**Supplementary Table 6. Representative clinical studies of BXD on functional dyspepsia**

| **Patients entered** | **Study design** | **Study length** | **Sample size** | **Observation Group** | | | **Main results** | **Reference** |
| --- | --- | --- | --- | --- | --- | --- | --- | --- |
|  |  |  |  | **Medicine Detail** | **Dosage** | **Preparation** | **Details** |  |
| functional dyspepsia | Randomized | 4 weeks | 40 | Pinelliae Rhizoma 15 g, Scutellariae radix 10 g, Coptidis Rhizoma 6 g, Zingiberis Rhizoma 6 g, Codonopsis Radix 15 g, Glycyrrhizae Radix et Rhizoma Praeparata cum Melle 10 g, 5 pieces of Jujubae Fructus.  For severe abdominal distension, add Citri Fructus 10 g, Citri Sarcodactylis Fructus 10 g.  For severer pain, add Paeoniae Radix Alba 10 g, Corydalis Rhizoma 10 g.  For acid vomiting, add Sepiae Endoconcha 10 g, Fritillariae Thunbergii Bulbus 10 g.  For severe blood stasis, add Curcumae Rhizoma 10 g, Notoginseng Radix et Rhizoma 3 g.  For those who suffer from anorexia, add Galli Gigerii Endothelium 15 g, Stir-fired Crataegi Fructus10 g. | 1 dose/ d, 2 times/d, 300ml | Decoction | BXD has a good effect on functional dyspepsia. Through χ2 tests showed that the efficacy of two different individual symptoms in the treatment of fullness, stomach pain, bitter mouth, and mouth pain, dryness, dizziness, nausea, and vomiting, the treatment group showed a significantly higher effective rate than the control group (P<0.05). | [1] |
| functional dyspepsia | Randomized | 2 weeks | 60 | Pinelliae Rhizoma 12 g, Scutellariae radix 5 g, Coptidis Rhizoma 10 g, Codonopsis Radix 10 g, Zingiberis Rhizoma 5 g, Magnoliae Officinalis Cortex 10 g, Amomi Fructus 10 g, Paeoniae Radix Alba 10 g, Citri Reticulatae Pericarpium 10 g, 5 pieces of Jujubae Fructus, Atractylodis Macrocephalae Rhizoma 10 g | 3times/d, 100ml/t | Decoction | The total efficiency of the treatment group was 90%, while the effective rate of the control group was 86.7%. After treatment, there was a significant improvement in traditional Chinese medicine syndromes between the two groups, with a significant difference compared to before treatment (p<0.01). | [2] |
| Functional dyspepsia | Randomized | 4 weeks | 120 | Pinelliae Rhizoma Praeparatum cum Alumine 12 g, Scutellariae radix 10 g, Coptidis Rhizoma 5 g, Codonopsis Radix 15 g, Zingiberis Rhizoma 10 g, Glycyrrhizae Radix et Rhizoma Praeparata cum Melle 10 g, 4 pieces of Jujubae Fructus | 2 times/d, 150ml/t | Decoction | BXD treatment of FD can significantly relieve patients' symptoms and effectively regulate their gastrointestinal motility function. The total effective rate of the treatment group was 61.67%, while the total effective rate of the control group was 55%. There was a significant statistical difference (p<0.05) in the symptom score between the treatment group and the control group. | [3] |
| Functional dyspepsia | Randomized | 1 month | 96 | Scutellariae radix 15 g, Pinelliae Rhizoma 15 g, Ginseng Radix et Rhizoma 10 g, Curcumae Longae Rhizoma 10 g, Pogostemonis Herba 10 g, Glycyrrhizae Radix et Rhizoma 10 g, Zingiberis Rhizoma 10 g, Coptidis Rhizoma 6 g, 6 pieces of Jujubae Fructus.  For patients with excessive heat, add Hedyotidis Diffusae Herba and Taraxacai Herba.  For patients with depression, add small amounts of Curcumae Radix and Albiziae Flos.  For patients with anorexia, add Stir-fired Messa Medicata Fermentata and Hordei Fructus Germinatus. | 1 dose/d | Decoction | BXD treatment of FD can improve the clinical efficacy and quality of life of patients. The clinical efficacy of the control group and the observation group after treatment was 68.75% and 95.83%, respectively. The clinical efficacy indicators of the control group were lower than those of the observation group, and the data difference was statistically significant (P<0.05). The sleep quality indicators of the observation group and the control group after treatment were (69.45 ± 6.12) and (49.48 ± 6.09), respectively. The sleep quality indicators of the observation group were relatively high. The indicators of mental state, activity, and appetite in the observation group were higher than those in the control group, and the data difference was statistically significant (P<0.05) | [4] |
| Functional dyspepsia | Randomized | 6 weeks | 90 | Scutellariae radix 10 g, Codonopsis Radix 15 g, Jujubae Fructus 10 g, Pinelliae Rhizoma Praeparatum cum Zingibere et Alumine 10 g, Coptidis Rhizoma 6 g, Zingiberis Rhizoma 10 g, Glycyrrhizae Radix et Rhizoma Praeparata cum Melle 6 g.  For abdominal pain, add Toosendan Fructus 8 g, Stir-fried Paeoniae Radix Alba 10 g, Aucklandiae Radix 8 g.  For abdominal belching and fullness, add Magnoliae Officinalis Cortex 10 g, Poria 15 g, Aurantii Fructus Immaturus 10 g.  For emotional anxiety, add Citri Sarcodactylis Fructus 10 g, Bupleuri Radix 8 g, Curcumae Radix 10 g.  For patients with acid reflux, add Sepiae Endoconcha 20 g.  For those with thick tongue coating, add Atractylodis Macrocephalae Rhizoma 10 g, Coicis Semen 15 g, Amomi Fructus 10 g.  For patients with anorexia, add Fried Hordei Fructus Germinatus 15 g, Acori Tatarinowii Rhizoma 10 g, Galli Gigerii Endothelium 10 g. | 100 ml /time,3 times/ d | Decoction | In terms of clinical efficacy, The total effective rate of the control group is lower than that of the observation group, and the difference is statistically significant（χ2=4.050, P=0.044). The quality of life score of the control group was lower than that of the observation group, and the difference was statistically significant (P<0.05). | [5] |
| Functional dyspepsia | Randomized | 4 weeks | 61 | Pinelliae Rhizoma Praeparatum 12 g, Zingiberis Rhizoma10 g, Codonopsis Radix 15 g, Scutellariae radix 12 g, Coptidis Rhizoma 6 g, Glycyrrhizae Radix et Rhizoma Praeparata cum Melle 8 g, 3 pieces of Jujubae Fructus.  For patients with obvious gastric bloating, add Aurantii Fructus, Bupleuri Radix.  For patients with obvious abdominal pain, add Toosendan Fructus, Corydalis Rhizoma.  For patients with acid reflux, add Sepiae Endoconcha, Euodiae Fructus.  For patients with anorexia, add Stir-fired Hordei Fructus Germinatus, Galli Gigerii Endothelium.  For those with loose stools, add fried Coicis semen and stir-fried Dioscoreae Rhizoma.  For those with dry mouth, reduce the dose of Zingiberis Rhizoma and add Glehniae Radix, Ophiopogonis Radix.  For those with poor sleep at night, add Albiziae Cortex. | 2 times/d, 200ml/t | Decoction | The total effective rate of the treatment group was 90.32%, while the total effective rate of the control group was 76.67%. The treatment group was better than the control group (P<0.05). In terms of improving symptoms such as epigastric fullness, acid reflux, loose stools, and pale yellow tongue, the treatment group was superior to the control group (P<0.05) | [6] |
| Functional dyspepsia | Randomized | 4 weeks | 98 | Pinelliae Rhizoma 9 g, Scutellariae radix 6 g, Zingiberis Rhizoma 6 g, Glycyrrhizae Radix et Rhizoma Praeparata cum Melle 6 g, Ginseng Radix et Rhizoma 6 g, Aurantii Fructus 6 g, Bupleuri Radix 6 g, Coptidis Rhizoma 3 g, 10 pieces of Jujubae Fructus | 2 times/d, 100ml/t | Decoction | The total effective rate of the control group was 63.27%, there was a significant statistical difference in the total effective rate of treatment between the two groups of patients (P<0.05). There was no significant statistical difference in gastric pH between the two groups of patients before treatment (P>0.05). The pH values in the stomach of both groups of patients after treatment were significantly higher than before treatment (P<0.05).After treatment, the pH value of the stomach in the treatment group was significantly higher than that in the control group (P<0.05). | [7] |
| Functional dyspepsia | Randomized | 4 weeks | 80 | Pinelliae Rhizoma 15 g, Scutellariae radix 9 g, Zingiberis Rhizoma 9 g, Ginseng Radix et Rhizoma 9 g, Glycyrrhizae Radix et Rhizoma Praeparata cum Melle 9 g, Coptidis Rhizoma 3 g, 4 pieces of Jujubae Fructus. | 2 times/d | Decoction | The clinical efficacy and safety of BXD in the treatment of elderly patients with FD are worthy of recognition. After 2 and 4 weeks of treatment, the symptom scores of upper abdominal distension, acid reflux, belching, and loss of appetite in the two groups of patients were lower than before treatment, and the observation group was lower than the control group (P<0.05). The serum MTL and CCK levels of both groups of patients were higher than before treatment, and the observation group was higher than the control group (P<0.05). | [8] |
| Functional dyspepsia | Randomized | 14 days | 90 | Pinelliae Rhizoma 10 g, Astragali Radix 10 g, Zingiberis Rhizoma 10 g, Glycyrrhizae Radix et Rhizoma 10 g, Coptidis Rhizoma 4 g, 4 pieces of Jujubae Fructus.  For those with vomiting, Aurantii Fructus Immaturus 10 g, Haematitum 30 g and Raphani Semen 15 g were added.  For those with anorexia, Amomi Fructus 6 g, Citri Reticulatae Pericarpium10 g were added.  For those with diarrhea and loose stools, Stir-fired Crataegi Fructus 6 g, Zingiberis Rhizoma Praeparatum 6 g and Zingiberis Rhizoma 6 g were added  For those with blood stasis, add Salviae Miltiorrhizae Radix et Rhizoma, Moutan Cortex. | 2 times/d | Decoction | BXD has a definite clinical effect in treating pediatric dyspepsia and can accelerate the recovery of intestinal function in children. The total effective rate of treatment for the experimental group of children was 91.1%, which was higher than the control group's 64.4%, and the difference was statistically significant (P<0.05). The upper abdominal pain, early satiety, belching, nausea, vomiting, and upper abdominal burning sensation scores of the two groups of children after treatment were lower than before treatment, and the difference was statistically significant (P<0.05). The scores of upper abdominal pain, early satiety, belching, nausea, vomiting, and upper abdominal burning sensation in the experimental group were lower than those in the control group after treatment. | [9] |
| Functional dyspepsia | Randomized | 30 days | 200 | Pinelliae Rhizoma 12 g, Coptidis Rhizoma6g, Scutellariae radix 12 g, Codonopsis Radix10 g, Zingiberis Rhizoma 6 g, Glycyrrhizae Radix et Rhizoma 6 g, Jujubae Fructus 10 g.  For those with severe heat, add Taraxacai Herba, add Coptidis Rhizoma and Scutellariae radix.  For those with severe cold and dampness, add Pinelliae Rhizoma and Zingiberis Rhizoma.  For those with spleen deficiency, increase the dosage of Codonopsis Radix.  For those with abdominal distension, add Aurantii Fructus Immaturus, Magnoliae Officinalis Cortex.  For those with acid reflux, add Fritillariae Thunbergii Bulbus.  For nausea and vomiting, add Bambusae Caulis in Taenias.  For anorexia, add Fried Raphani Semen.  For constipation, add Rhei Radix et Rhizoma. | 2 times/d, 100ml/t | Decoction | Symptom efficacy: The total effective rate of the treatment group was 94%, which was significantly different from the control group's total effective rate of 69% (p<0.05).  Gastroscopy treatment: The total effective rate of the treatment group was 70%, which was significantly different from the control group's total effective rate of 67% (p>0.05).  HP clearance status: 51 cases (65.4%) in the treatment group were positive to negative, while 42 cases (63.4%) in the control group were positive to negative. | [10] |

**References**

1. Lei Z., Hui C., Zhaoyuan S. Clinical Study of Banxiaxiexin Decoction in the Treatment of Functional Dyspepsia. Journal of Mathematical Medicine. 2019;32(10):1519-21.
2. Xiongli H., Di Z., Hong Z., Xiangping X. Clinical Study on Banxia Xiexin Decoction for Treating Functional Dyspepsia with Simultaneous Occurrence of the Cold and the Heat Syndrome. Journal of Hunan University of Chinese Medicine. 2006;26(01):40-1.
3. Chenxi L. Clinical Observation of Banxia Xiexin Decoction for Treating Functional Dyspepsia with Simultaneous Occurrence of the Cold and the Heat Syndrome. Guangming Journal of Chinese Medicine. 2020;35(04):482-4.
4. Liming Y. Clinical Observation of Banxia Xiexin Decoction for Treating Functional Dyspepsia. Yunnan Journal of Traditional Chinese Medicine and Materia Medica. 2019;40(08):100-1.
5. Chenghai C., Lele Y. Clinical Observation on Banxia Xiexin Decoction in the Treatment of Functional Dyspepsia. Guangming Journal of Chinese Medicine. 2019;34(10):1530-2.
6. Liping Z., Xiufang S. Clinical Observation on Banxia Xiexin Decoction in the Treatment of Functional Dyspepsia. Guangming Journal of Chinese Medicine. 2017;32(10):1432-4.
7. Huating L., Fangming C. Clinical Observation of Using Banxia Xiexin Decoction in the Treatment of Functional Dyspepsia. Journal of Sichuan of Traditional Chinese Medicine. 2015;33(09):90-2.
8. Lifeng Z., Yanfang C., Xiaolin L. Clinical Effect and Safety Observation on Banxia Xiexin Decoction in the Treatment of Functional Dyspepsia in the Elderly. Chinese Journal of Clinical Rational Drug Use. 2019;12(07):88-9.
9. Pengfei Y. Clinical Effect of Banxia Xiexin Decoction in the Treatment of Children with Dyspepsia. China Modern Medicine. 2020;27(14):172-4.
10. Xiaoping D. Clinical Observation of Banxia Xiexin Decoction for Treating Non-Ulcerative Dyspepsia. Practical Clinical Journal of Integrated Traditional Chinese and Western Medicine. 2005;5(2):38-9.
11. Kim S. K., Joung J. Y., Ahn Y. C., Jung I. C., Son C. G. Beneficial Potential of *Banha-Sasim-Tang* for Stress-Sensitive Functional Dyspepsia via Modulation of Ghrelin: A Randomized Controlled Trial. Frontiers in pharmacology. 2021;12:636752.
12. Park J. W., Ryu B., Yeo I., Jerng U. M., Han G., Oh S., Lee J., Kim, J. *Banha-Sasim-Tang* as an Herbal Formula for the Treatment of Functional Dyspepsia: A Randomized, Double-Blind, Placebo-Controlled, Two-Center Trial. Trials. 2010;11:83.
13. Kim Y. H., Kim J. Y., Kwon O. J., Jung S. Y., Joung J. Y., Yang C. S., Lee J. H., Cho J. H., Son C. G. Efficacy of a Traditional Herbal Formula, Banha-Sasim-Tang in Functional Dyspepsia Classified as Excess Pattern. Frontiers in pharmacology. 2021;12:698887.
14. Kim Y. H., Kim J. Y., Jung S. Y., Kwon O. J., Lee J. H., Son C. G. Efficacy of *Banha-Sasim-Tang* on Functional Dyspepsia Classified as Excess Pattern: Study Protocol for a Randomized Controlled Trial. Trials. 2017;18(1):525.
15. Park J. W., Ko S. J., Han G., Yeo I., Ryu B., Kim, J. The Effects of *Banha-Sasim-Tang* on Dyspeptic Symptoms and Gastric Motility in Cases of Functional Dyspepsia: A Randomized, Double-Blind, Placebo-Controlled, and Two-Center Trial. Evidence-Based Complementary and Alternative Medicine. 2013, 265035.

**5. Clinical study of BXD on diabetes related indications**

**Supplementary Table 7. Representative clinical studies of BXD on diabetes related indications**

| **Patients entered** | **Study design** | **Study length** | **Sample size** | **Observation Group** | | | **Main results** | **Reference** |
| --- | --- | --- | --- | --- | --- | --- | --- | --- |
|  |  |  |  | **Medicine Detail** | **Dosage** | **Preparation** | **Details** |  |
| nonalcoholic fatty liver disease in type 2 diabetes mellitus | Randomized | 4 weeks | 64 | Puerariae Lobatae Radix 12 g, Ophiopogonis Radix 12 g, Processed Pinelliae Rhizoma 12 g, Codonopsis Radix 15 g, Scutellariae Radix 15 g, Coptidis Rhizoma, Glycyrrhizae Radix et Rhizoma 6 g, Zingiberis Rhizoma 6 g.  For severe blood stasis, add Paeoniae Radix Rubra, Salviae Miltiorrhizae Radix et Rhizoma and Carthami Flos.  For patients with spleen and kidney deficiency, add Rehmanniae Radix Praeparata, Schisandrae Chinensis Fructus and Astragali Radix. | 1 dose/ d, 2 times/ d | Decoction | The liver function indexes ALT and AST were 20.25±2.16 IU/ L, 23.43±1.96 IU/ L respectively, compared with 32.93±1.64 IU/ L, 31.15±3.42 IU/ L in control group. The blood sugar 2 hours after a meal was 8.06±1.18 mmol/ L, compared with 10.15±1.12 mmol/ L in control group. | [1] |
| diabetic gastroparesis with coldheat complex | Randomized | 8 weeks | 84 | Pinelliae Rhizoma Rraeparatum cum Alumine 12 g, Coptidis Rhizoma 10 g, Scutellariae Radix 10 g, Ginseng Radix et Rhizoma 10 g, Zingiberis Rhizoma 10 g, Glycyrrhizae Radix et Rhizoma Praeparata cum Melle 6 g, 3 pieces of Jujubae Fructus. | 1 dose/ d, 2 times/ d, 200 ml/ time | Decoction | BXD has efficacy in treating diabetic gastroparesis and can effectively improve the levels of gastrointestinal hormones and inflammatory factors in patients. The total effective rate was 97.62%, compared with 85.71% total effective rate in control group. The GAS, G-17, IL-6 levels and TCM symptom scores of the two groups of patients were all lower than before treatment (P<0.05), and the observation group was lower than the control group (P<0.05); SS and IL-10 levels were higher than before treatment (P<0.05). | [2] |
| type 2 diabetes mellitus with spleen deficiency and stomach stagnation | Randomized | 3 months | 84 | Pinelliae Rhizoma praeparatum 15 g, Salviae Miltiorrhizae Radix et Rhizoma 15 g, Jujubae Fructus 12 g, Zingiberis Rhizoma 10 g, Coptidis Rhizoma 12 g, Glycyrrhizae Radix et Rhizoma Praeparata cum Melle 8 g, Scutellariae Radix 10 g.  For patients with shortness of breath, add Astragali Radix 10 g, Stir-fried Atractylodis 10 g.  For patients with loose stools, remove Zingiberis Rhizoma, add Zingiberis Rhizoma Recens 30 g, Coicis Semen 30 g.  For patients with constipation and intestinal stasis, add Cannabis Fructus 12 g, Pruni Semen 12 g. | 1 dose/ d, 2 times/ d, 200 ml/ time | Decoction | The total effective rate was 97.62%, compared with 80.95% total effective rate in control group. The experiment also tested the patients' OCTT, HbA1c, and WBC. The above indicators in the BXD treatment group were lower than those in the control group (p<0.05). | [3] |
| diabetic gastro paresis | Randomized | 3 months | 62 | Pinelliae Rhizoma Praeparatum cum Zingibere et Alumine 15 g, Scutellariae Radix 15 g, Codonopsis Radix 15 g, Coptidis Rhizoma 5 g, Zingiberis Rhizoma 15 g, Aurantii Fructus 15 g, Magnoliae Officinalis Cortex 15 g, Glycyrrhizae Radix et Rhizoma Praeparata cum Melle 10 g.  For patients with obvious thirst, add PuerariaeLobatae Radix 15 g, Polygonati Odorati Rhizoma 10 g, Dendrobii Caulis 10 g.  For patients with waterbrash, add Sepiae Endoconcha 15 g, Powered Meretricis Concha Cyclinae Concha 30 g.  For patients with hiccup and nausea, add Inulae Flos 12 g, Zingiberis Rhizoma Recens 6 g. | 1 dose/ d, 2 times/ d, 200 ml/ time | Decoction | The total effective rate was 90.32%, compared with 77.42% total effective rate in control group. There were statistically significant differences between the control group and the treatment group in terms of main symptoms, physical sign scores, gastric emptying time, FBG, 2hPG, HBA1C, etc. | [4] |
| diabetic gastro paresis | Randomized | 1 month | 50 | Pinelliae Rhizoma 12 g, Atractylodis 12 g, Codonopsis Radix 12 g, Scutellariae Radix 9 g, Zingiberis Rhizoma 9 g, Hordei Fructus Germinatus and Shenqu 9 g, Glycyrrhizae Radix et Rhizoma Praeparata cum Melle 6 g and Coptidis Rhizoma 6 g, 6 pieces of Jujubae Fructus. | 1 dose/ d, 2 times/ d | Decoction | BXD is effective in treating diabetic gastroparesis, and it is well tolerated by patients. The total effective rate was 96.0%, compared with 72.0% total effective rate in control group. The evaluation indicators of the experiment include gastric emptying time, glycosylated hemoglobin level, and blood rheology analysis. | [5] |
| diabetic gastro paresis | Randomized | 4 weeks | 58 | Pinelliae Rhizoma Praeparatum cum Zingibere et Alumine 12 g, Scutellariae Radix, Ginseng Radix et Rhizoma 10 g, Zingiberis Rhizoma 10 g, Jujubae Fructus 10 g, Coptidis Rhizoma 8 g, Glycyrrhizae Radix et Rhizoma Praeparata cum Melle 8 g. | 1 dose/ d, 2 times/ d | Decoction | BXD has a significant effect in treating diabetes combined with gastroparesis. It can improve patients' clinical symptoms and reduce their blood sugar levels. The total effective rate was 93.10%, compared with 72.41% total effective rate in control group. | [6] |
| diabetic gastro paresis | Randomized | 1 month | 60 | Pinelliae Rhizoma Praeparatum cum Zingibere et Alumine 15 g, Coptidis Rhizoma 6 g, Scutellariae Radix 8 g, Zingiberis Rhizoma 6 g, Glycyrrhizae Radix et Rhizoma 6 g, Ginseng Radix et Rhizoma 10 g, Citri Reticulatae Pericarpium 10 g, Magnoliae Officinalis Cortex 12 g, 10 pieces of Jujubae Fructus. | 1 dose/ d, 2 times/ d | Decoction | The mechanism of action of BXD in treating diabetic gastroparesis may be related to promoting SS secretion and inhibiting GAS secretion. The total effective rate was 86.7%, compared with 63.3% total effective rate in control group. | [7] |
| diabetic gastro paresis | Randomized | 4 weeks | 48 | On the basis of basic treatment (diabetic diet, oral hypoglycemic drugs, insulin treatment when necessary), BXD treatment is used. Pinelliae Rhizoma 12 g, Scutellariae Radix 10 g, Coptidis Rhizoma 8 g, Zingiberis Rhizoma 10 g, Ginseng Radix et Rhizoma 10 g, Glycyrrhizae Radix et Rhizoma Praeparata cum Melle 8 g, Jujubae Fructus 10 g. | 1 dose/ d, 2 times/ d, 100 ml/ time | Decoction | BXD has definite efficacy in treating diabetic gastroparesis and can reduce fasting blood sugar. The total effective rate was 91.67%, compared with 66.67% total effective rate in control group. | [8] |
| nonalcoholic fatty liver disease in type 2 diabetes mellitus | Randomized | 3 months | 66 | Processed Pinelliae Rhizoma 12 g, Ophiopogonis Radix 12 g, puerariae lobatae radix 12 g, Scutellariae Radix 15 g, Codonopsis Radix 15 g, Glycyrrhizae Radix et Rhizoma 6 g, Zingiberis Rhizoma 6 g, Coptidis Rhizoma 6 g.  For those with severe blood stasis, add Salviae Miltiorrhizae Radix et Rhizoma, Carthami Flos and Paeoniae Radix Rubra. | 1 dose/ d, 2 times/ d | Decoction | BXD has obvious clinical effects in treating non-alcoholic fatty liver disease combined with diabetes, and can effectively improve patients' blood sugar and liver function indicators. The liver function indexes ALT and AST were 21.25±1.02 IU/ L, 24.02±1.85 IU/ L respectively, compared with 33.56±1.98 IU/ L, 30.45±1.85 IU/ L in control group. | [9] |

**References**

1. Yongmin Z., Guojun Z., Juhong Z. Clinical Study on Banxia Xiexin Decoction for Treating Diabetic Non-Alcoholic Fatty Liver Disease. Psychological Monthly. 2019;14(03):165.
2. Peixian X., Junjun W., Genyuan C., Yongchang H. Clinical Observation on the Treatment of Diabetic Gastroparesis with Cold-Heat Mismatch by Banxia Xiexin Decoction. China's Naturopathy. 2022;30(15):73-6.
3. Ming X. Clinical Observation on the Treatment of Type 2 Diabetes Mellitus with Spleen Deficiency and Gastric Stagnation with Banxia Xiexin Decoction. Guangming Journal of Chinese Medicine. 2021;36(06):933-4.
4. Na L., Chunmei D., Maer H. Clinical Effect of Banxia Xiexin Decoction in Treatment of Diabetic Gastroparesis: An Analysis of 31 Cases. Hunan Journal of Traditional Chinese Medicine. 2020;36(02):1-3.
5. Bo L. Clinical Observation on Banxia Xiexin Decoction in Treatment of Diabetic Gastroparesis. Journal of Aerospace Medicine. 2018;29(09):1141-2.
6. Songlin H., Shiyun L., Junfei L. Clinical Observation on Banxia Xiexin Decoction in the Treatment of Diabetes Complicated with Gastroparesis. Guangming Journal of Chinese Medicine. 2018;33(01):59-61.
7. Jing Z. Jian Z. Clinical Observation on Ban Xia Xie Xin Decoction in Treating Diabetic Gastroparesis. Western Journal of Traditional Chinese Medicine. 2014;27(11):104-6.
8. Jianping Z., Xifang P. Clinical Observation on Banxia Xiexin Decoction in Treating Diabetic Gastroparesis. Inner Mongolia Journal of Traditional Chinese Medicine. 2009;28(14):6.
9. Zhengbo Z. Analysis of the Clinical Effects of Banxia Xiexin Decoction in the Treatment of Non-Alcoholic Fatty Liver with Diabetes. Diabetes New World. 2020;23(21):89-91.

**Supplementary Table 8. Representative clinical trials of BXD on reflux disease**

| **Patients entered** | **Study design** | **Study length** | **Sample size** | **Observation Group** | | | **Main results** | **Reference** |
| --- | --- | --- | --- | --- | --- | --- | --- | --- |
|  |  |  |  | **Medicine Detail** | **Dosage** | **Preparation** | **Details** |  |
| Gastroesophageal reflux cough | Randomized | 4 weeks | 70 | On the basis of the control group, combined with BXD for treatment: Processed Pinelliae Rhizoma 9 g, Coptidis Rhizoma 6 g, Scutellariae Radix 6 g, Pseudostellariae Radix 9 g, Armeniacae Semen Amarum 9 g, Honey-fried Eriobotryae Folium 9 g, Zingiberis rhizome 3 g, Perillae Fructus 9 g, Jujubae Fructus 9 g, Glycyrrhizae Radix et Rhizoma 3 g | 1 dose/ d,  100ml/ t,  2 times/ d. | Decoction | BXD is effective in treating gastroesophageal reflux cough. It can effectively reduce patients’ clinical symptoms such as cough and reflux, and improve the overall treatment efficiency. The experiment evaluated CSS scores, RDQ scores, and clinical symptom scores. The results showed that after treatment, the scores of the BXD treatment group were lower than those of the control group (P<0.05). | [1] |
| Reflux esophagitis | Randomized | 4 weeks | 68 | Pinelliae Rhizoma Praeparatum 15 g, Zingiberis Rhizoma 10 g, Scutellariae Radix 10 g, Codonopsis Radix 10 g, Coptidis Rhizoma 3 g, Inulae Flos 10 g, Glycyrrhizae Radix et Rhizoma Praeparata cum Melle 6 g, Haematitum 10 g, Jujubae Fructus 10 g.  For patients with epigastric pain, add Aucklandiae Radix 8 g and Toosendan Fructus 8 g.  For patients with acid reflux, add Sepiae Endoconcha 10 g, Hordei fructus germinates 15 g, Galli Gigerii Endothelium Corneum 10 g.  For patients with gastric distension, add Aurantii Fructus Immaturus 10 g, Magnoliae Officinalis Cortex 10 g, Poria 15 g.  For patients with anorexia, add Poria 15 g and Acori Tatarinowii Rhizoma 10 g.  For patients with anxiety, add Bupleuri Radix 8 g and Curcumae Radix 10 g. | 1 dose/ d,  300 ml/ dose | Decoction | BXD adjustable treatment can effectively treat reflux esophagitis and relieve patients' clinical symptoms such as stomach pain and acid reflux. The total clinical effective rate was 85.2% in the observation group and 73.5% in the control group. The improvement of symptoms such as acid reflux, heartburn and dysphagia in observation group was significantly better than that in control group. After 1 month follow-up, the recurrence rate was 23.5% in the observation group and 50.0% in the control group. | [2] |
| Reflux Esophagitis of Intermingled Cold and Heat Type | Randomized | 3 months | 68 | Pinelliae Rhizoma Praeparatum 10 g, Coptidis Rhizoma 6 g, Scutellariae Radix 10 g, Zingiberis Rhizoma 10 g, Glycyrrhizae Radix et Rhizoma 6 g, Ginseng Radix et Rhizoma 10 g, 4 pieces of Jujubae Fructus. | 1 dose/ d,  100ml/ dose,  2 times/ d. | Decoction | BXD can regulate the level of gastrointestinal hormones in patients, relieve epigastric pain, burning, acid reflux, nausea and other symptoms, and can effectively treat reflux esophagitis. The experiment evaluated three aspects: TCM syndrome scores, gastrointestinal hormone levels, and clinical efficacy. The total effective rate was 94.12% in the treatment group and 76% in the control group. | [3] |
| Reflux esophagitis | Randomized | 8 weeks | 80 | Coptidis Rhizoma 10 g, 4 pieces of Jujubae Fructus, Pinelliae rhizome 12 g, Ginseng Radix et Rhizoma 6 g, Scutellariae Radix 6 g, Zingiberis Rhizoma 18 g, Glycyrrhizae Radix et Rhizoma Praeparata cum Melle 6 g.  Patients with Qi stagnation and phlegm obstruction should be treated with basic prescription.  For patients with spleen-stomach dampness syndrome, add Microctis Folium 10 g.  For patients with blood stasis blocking collaterals syndrome, add Carthami Flos 5 g, Persicae Semen 7 g, Angelicae Sinensis Radix 10 g. | 1 dose/ d,  200ml/ dose, 100ml/ t,  2 times/ d | Decoction | The clinical efficacy of BXD therapy in treating patients with reflux esophagitis is significant, and it can effectively improve the patient’s symptom score. The total effective rate of clinical treatment in observation group was 97.50%, which was significantly higher than 82.50% in control group. | [4] |
| Gastroesophageal reflux disease | Randomized | 8 weeks | 116 | Pseudostellariae Radix 15 g, Pinelliae rhizome 10 g, Coptidis Rhizoma 9 g, Scutellariae Radix 9 g, Zingiberis Rhizoma 6 g, Glycyrrhizae Radix et Rhizoma Praeparata cum Melle 6 g, Jujubae Fructus 10 g, Taraxaci Herba 15 g, Bletilla Striata 15 g.  For patients with severe waterbrash, add Sepiae Endoconcha.  For patients with severe bloating, add Raphani Semen, Arecae Semen and Magnoliae Officinalis Cortex.  For patients in pain, add Corydalis rhizome and Toosendan Fructus. | 1 dose/ d,  400ml/ dose, 200ml/ t,  2 times/ d | Decoction | BXD has reliable efficacy in treating gastroesophageal reflux disease and can improve patients’ clinical symptoms and signs. The total effective rate was 93.10% in the observation group and 77.59% in the control group. The clinical symptom score of the observation group was reduced more than that of the control group. | [5] |
| Superficial gastritis with bile reflux and anxiety symptoms | Randomized | 8 weeks | 118 | Pinelliae Rhizoma Praeparatum 10 g, Codonopsis Radix 15 g, Coptidis Rhizoma 5 g, Scutellariae Radix 15 g, Zingiberis Rhizoma 5 g, Glycyrrhizae Radix et Rhizoma 5 g.  For patients with upper abdominal pain, add Notoginseng Radix et Rhizoma 3 g and Corydalis rhizome 10 g.  For patients with abdominal distension, add Citri Reticulatae Pericarpium Viride 10 g and Aucklandiae Radix 10 g.  For patients with waterbrash, add Sepiae Endoconcha 20 g.  For patients with severe vomiting, add Bambusae Caulis in Taenias 10 g and Inulae Flos 10 g.  For patients with loose stool, add Coicis Semen 10 g and Atractylodis rhizome 10 g. | 1 dose/ d,  200ml/ dose,  2 times/ d | Decoction | BXD has significant effects on superficial gastritis with bile reflux and anxiety symptoms. The total effective rate for chronic gastritis with bile reflux was 57.95% in the treatment group and 47.81% in the control group. | [6] |
| Bile reflux gastritis | Randomized | 4 weeks | 90 | Pinelliae rhizome 15 g, Coptidis Rhizoma 12 g, Scutellariae Radix 12 g, Aurantii Fructus Immaturus 15 g, Amomi Fructus 15 g, Zingiberis Rhizoma 6 g, Euodiae Fructus 6 g, Aquilariae Lignum Resinatum 6 g, Trogopterus Dung 15 g, Wine-steamed Rhei Radix et Rhizoma 9 g, Glycyrrhizae Radix et Rhizoma Praeparata cum Melle 6 g | 1 dose/ d,  2 times/ d | Decoction | BXD has a good therapeutic effect on bile reflux gastritis. The total effective rate was 95.83% in the observation group and 66.67% in the control group. BXD is better than the control group in improving the symptoms of bile reflux gastritis, such as stomachache, fullness, belching, noisy and bitter mouth. | [7] |
| Reflux esophagitis | Randomized | 8 weeks | 66 | Pinelliae rhizome 10 g, Coptidis Rhizoma 10 g, Codonopsis Radix 10 g, Glycyrrhizae Radix et Rhizoma 6 g, Scutellariae Radix 10 g, Zingiberis Rhizoma 10 g, Jujubae Fructus 15 g, Haematitum 25 g, Sepiae Endoconcha 10 g, Aurantii Fructus 10 g. | 1 dose/ d,  400ml/ dose,  2 times/ d. | Decoction | The total effective rate was 88.9% in the observation group and 73.3% in the control group. | [8] |
| Gastroesophageal reflux disease | Randomized | 8 weeks | 96 | Pinelliae rhizome 12 g, Scutellariae Radix 10 g, Codonopsis Radix 15 g, Zingiberis Rhizoma 5 g, 4 pieces of Jujubae Fructus, Glycyrrhizae Radix et Rhizoma Praeparata cum Melle 5 g.  For patients with stomach excessive heat, remove the Zingiberis Rhizoma and add Moutan Cortex 10 g. | 2 times/ d,  100ml/ time | Decoction | BXD has a reliable clinical effect in treating gastroesophageal reflux disease The effective rates of study group and control group were 100% and 83.3%, respectively. The scores of TCM symptoms of the two groups were compared: the study group changed from 16.4±3.2 before treatment to 2.8±0.2 after treatment, and the control group changed from 16.5±3.1 before treatment to 7.4±1.9 after treatment. | [9] |
| Gastroesophageal reflux | Randomized | Not mentioned | 113 | 4 pieces of Jujubae Fructus, Coptidis Rhizoma 3 g, Ginseng Radix et Rhizoma 6 g, Glycyrrhizae Radix et Rhizoma Praeparata cum Melle 6 g, Zingiberis Rhizoma 6 g, Scutellariae Radix 6 g, Pinelliae rhizome 9 g. | 1 dose/ d,  300ml/ dose,  3 times/ d | Decoction | The therapeutic effect of BXD is significantly better than that of rabeprazole sodium enteric-coated tablets, which not only improves the prognosis of patients, but also improves the quality of life of patients. The total effective rate was 96.49% in the observation group and 71.43% in the control group. | [10] |

**References**

1. Li S., Ying X., Clinical Study on Modified Banxia Xiexin Decoction in the Treatment of Gastroesophageal Reflux Cough. Guangming Journal of Chinese Medicine. 2020;35(16):2489-91.
2. Songwen T. Clinical Research on the Treatment of Reflux Oesophagitis by Acridness Relieving Superficies and Bitterness Expelling Internal Heat Method. Asia-Pacific Traditional Medicine. 2017;13(14):113-4.
3. Dan L., Clinical Observation on Banxia Xiexin Decoction in the Treatment of Reflux Esophagitis of Intermingled Cold and Heat symptom. Chinese Medicine Modern Distance Education of China. 2022;20(21):76-7.
4. Haidan L., Zhiwei Z., Huanlong H. Clinical Observation on the Treatment of Reflux Oesophagitis with Banxia Xiexin Decoction. Shenzhen Journal of Integrated Traditional Chinese and Western Medicine. 2021;31(07):61-2.
5. Luhong T. Clinical Observation on Treating Gastroesophageal Reflux Disease with Banxia Xiexin Decoction. Clinical Journal of Chinese Medicine. 2014;6(16):103-4.
6. Yingsi H. Clinical Observation on the Treatment of Patients with Superficial Gastritis with Bile Reflux and Anxiety Symptoms by Banxia Xiexin Decoction. Modern Interventional Diagnosis and Treatment in Gastroenterology. 2007;12(02):114-5.
7. Yunyu Z., Hongyi L., Shuhong C. Clinical Observation on the Treatment of Bile Reflux Gastritis with Banxia Xiexin Decoction. Clinical Journal of Traditional Chinese Medicine. 2007;19(02):139-40.
8. Yinghui L., Huanyu C. Clinical Observation on 36 Cases of Reflux Oesophagitis Treated with Banxia Xiexin Decoction. Liaoning Journal of Traditional Chinese Medicine. 2006;33(04):436.
9. Ting W. To Observe the Clinical Effect of Banxia Xiexin Decoction in Treatment of Gastroesophageal Reflux Disease. Inner Mongolia Journal of Traditional Chinese Medicine. 2015;34(12):12-3.
10. Junlin L. Clinical Observation on 113 Cases of Gastroesophageal Reflux Treated with Banxia Xiexin Decoction. Clinical Journal of Chinese Medicine. 2014;6(03):109-10.
11. Jiaping W., Wentai Z., Renyan G., Guohong S., Huimin L. Clinical Observation on the Treatment of Gastro-Oesophageal Reflux Disease with Banxia Xiexin Decoction. Journal of Emergency in Traditional Chinese Medicine. 2016;25(09):1828-9.
12. Xiying C. Clinical Observation on the Treatment of Chronic Gastric Disease with Banxia Xiexin Decoction. Guangming Journal of Chinese Medicine. 2017;32(23):3418-20.
13. Takeuchi T., Hongo H., Kimura T., Kojima Y., Harada S., Ota K., Takeuchi N., Noguchi T., Inoue T., Murano M., Higuchi K. Efficacy and Safety of Hangeshashinto for Treatment of GERD Refractory to Proton Pump Inhibitors: Usual Dose Proton Pump Inhibitors Plus Hangeshashinto Versus Double-Dose Proton Pump Inhibitors: Randomized, Multicenter Open Label Exploratory Study. Journal of gastroenterology. 2019;54(11):972-83.

**Supplementary Table 9. Representative clinical trials of BXD on IBS**

| **Patients entered** | **Study design** | **Study length** | **Sample size** | **Observation Group** | | | **Main results** | **Reference** |
| --- | --- | --- | --- | --- | --- | --- | --- | --- |
|  |  |  |  | **Medicine Detail** | **Dosage** | **Preparation** | **Details** |  |
| Diarrhea-predominant of irritable bowel syndrome | Randomized | 4 weeks | 120 | Pinelliae Rhizoma 8 g, Jujubae Fructus 6 g, Zingiberis Rhizoma 6 g, Coptidis Rhizoma 10 g, Glycyrrhizae Radix et Rhizoma Praeparata cum Melle 6 g, Scutellariae Radix 12 g | 1 dose/ d,  2 times/ d | Dispensing granules | BXD may regulate gastrointestinal motility function by affecting the release of Ghrelin, thereby relieving abdominal pain and abdominal discomfort. In the treatment group, the total effective rate was 83.33%. In the control group, the total effective rate was 80.00%. The experiment evaluated the comparison of plasma and colon mucosa Ghrelin before and after treatment. | [1] |
| Diarrhea-predominant of irritable bowel syndrome | Randomized | 4 weeks | 50 | Pinelliae Rhizoma 10 g, Scutellariae Radix 10 g, Codonopsis Radix 10 g, Atractylodis Macrocephalae Rhizoma 10 g, Poria 10 g, Citri Reticulatae Pericarpium 10 g, Zingiberis Rhizome 6 g, Coptidis Rhizome 5 g, Glycyrrhizae Radix et Rhizoma Praeparata cum Melle 3 g | 1 dose/ d,  2 times/ d. | Decoction | Treating D-IBS with BXD can relieve patients' symptoms, and its mechanism of action may be related to inhibiting 5-HT overexpression. The total effective rate was 93.33% in the treatment group and 75.00% in the control group. | [2] |
| Irritable bowel syndrome | Randomized | 10 days | 56 | Pinelliae Rhizoma Praeparatum 10~15 g, Zingiberis Rhizoma 10~15 g, Coptidis Rhizoma 6~10 g, Scutellariae Radix 8~10 g, Codonopsis Radix 10~15 g, Jujubae Fructus 10~12 g, Stir-fried Atractylodis Macrocephalae Rhizoma 10~12 g, Aucklandiae Radix 8~12 g, Paeoniae Radix Alba 10~15 g, Glycyrrhizae Radix et Rhizoma 6~10 g.  For patients with mainly watery stools, add Plantaginis Semen, Seed-coat of Hyacinth Dolichos, Polyporus and Alismatis rhizome.  For patients with predominantly mucoid stools, add Pulsatillae Radix and Phellodendri Chinensis Cortex.  For patients with abdominal distension, borborygmus, and pain, remove Codonopsis Radix, Atractylodis Macrocephalae Rhizoma and Jujubae Fructus, add Magnoliae Officinalis Cortex and Aurantii Fructus Immaturus.  For patients with constipation, add Rhei Radix et Rhizoma.  For patients with wet feces, add Coicis Semen and Pogostemonis herba.  For patients with vomiting and nausea, add Zingiberis Rhizome Recens and Bambusae Caulis in Taenias. | 1 dose/ d,  2 times/ d | Decoction | BXD is effective in treating irritable bowel syndrome. The total effective rate was 92.86%, and the recurrence rate was 9% after 1 year follow-up. | [3] |
| Irritable bowel syndrome | Randomized | 4 weeks | 87 | Pinelliae Rhizoma 9 g, Scutellariae Radix 6 g, Zingiberis Rhizoma 6 g, Ginseng Radix et Rhizoma 6 g, Glycyrrhizae Radix et Rhizoma Praeparata cum Melle 6 g, Coptidis Rhizoma 3 g, 4 pieces of Jujubae Fructus.  For patients with severe abdominal pain, add Corydalis Rhizoma and Toosendan Fructus.  For patients with severe diarrhea, add Mume Fructus and Chaenomelis Fructus.  For patients with severe bloating, add Arecae Semen, Aurantii Fructus Immaturus and Magnoliae Officinalis Cortex. | 1 dose/ d, 2 times/ d. | Decoction | BXD has good efficacy in treating irritable bowel syndrome. The total effective rate was 70.2% in the treatment group and 37.5% in the control group. | [4] |

**References**

1. Cheng Z., Feng P., Tao Z. A Clinical Study on Banxia Xiexin Decoction Treating Diarrhea Predominated Irritable Bowel Syndrome by Observing the Expression of Ghrelin. Chinese Archives of Traditional Chinese Medicine. 2011;29(11):2588-91.
2. Tianwen L., Xinlin C., Wangdong L., Lianghuan Z. Clinical Observation of Banxia Xiexin Tang for Diarrhea-Predominant of Irritable Bowel Syndrome. New Chinese Medicine. 2016;48(08):76-9.
3. Duomeng L. Clinical Study on 56 Cases of Banxia Xiexin Decoction for Irritable Bowel Syndrome. Chinese Journal of Traditional Medical Science and Technology. 1998;5(01):45.
4. Yong Z., Xiongzhen L., Lehong L. Clinical Effect Observation on Banxia Xiexin Decoction in Treating Irritable Bowel Syndrome. China Medical Herald. 2009;6(19):127-8.

**Supplementary Table 10. Representative clinical trials of BXD on oral muscositis**

| **Patients entered** | **Study design** | **Study length** | **Sample size** | **Observation Group** | | | **Main results** | **Reference** |
| --- | --- | --- | --- | --- | --- | --- | --- | --- |
|  |  |  |  | **Medicine Detail** | **Dosage** | **Preparation** | **Details** |  |
| Patients with gastric cancer who developed moderate to severe oral mucositis (CtCae v4.0 grade ≧1) during any cycle of chemotherapy | Randomized | 2–6 weeks according to the chemotherapy regimen from the beginning of the next course of chemotherapy | 91 | TJ-14 (Pinelliae tuber, Scutellariae radix, glycyrrhizae radix, Zizyphi Fructus, ginseng radix, Zin giberis Processum rhizoma, and Coptidis rhizome) | dose of 2.5 g/three times per day | Solution | Treatment with TJ-14 did not exhibit any effect with regard to reduc ing the frequency of grade 2 events or the duration of grade 2 chemotherapy-induced oral mucositis in gastric cancer patients receiving fluorinated pyrimidine-based chemo therapy. | [1] |
| Patients with head and neck cancer All patients underwent conventional fractioned radiation (2 Gy per day and 5 days per week) for their whole neck including oral cavity, phar ynx, and larynx with or without concurrent chemotherapy. The range of total radiation dose was 60–72 Gy (mean 66.5 Gy). | Controlled clinical trial | Along with the chemotherapy, not specified | 80 | TJ-14 (Pinellia Tuber, Scutellariae Radix, GlycyrrhizaeRadix, Zizyphi Fructus, Ginseng Radix, Zingiberis Processum Rhizoma, and Coptidis Rhizoma) | a 100-mL oral rinse solution containing 2.5-g TJ-14 in water .3 times/d (after meals) during and after the (chemo)radiation period | Solution | TJ-14 is effective for ameliorating oral mucositis induced by (chemo)radiation in patients with head and neck cancers. TJ-14 was associated with improved completion rates of chemoradiation treatments with cisplatin. A randomized controlled trial is necessary to confirm the efficacy of TJ-14 for chemoradiation-induced mucositis in head and neck cancer patients. | [2] |
| Patients with colorectal cancer who developed moderate-to-severe COM (WHO grade ≧1) during any cycle of chemotherapy using FOLFOX, FOL FIRI, and/or XELOX treatment | Multi-institutional, double-blind, placebo-controlled, randomized  comparative trial | 2 weeks | 93 | TJ-14 (The details not speficied) | 2.5 g /3 times per day, dissolve 2.5 g of TJ-14 or placebo in 50 ml of drinking water | Solution | TJ-14 demonstrated a significant effect in the treatment of grade ≧2 mucosi tis in patients with colorectal cancer compared to the placebo. | [3] |
| Patients with head and neck cancer who were treated with induction chemotherapy | Double-blind, randomized trial | 14 d | 16 | TJ-14 (The details not speficied) | 2.5 g after each meal/ three times per day, 2.5 g of TJ-14 in 100 ml of drinking water, rinse mouths with the solution for 30 s. | Solution | The incidence of grade 2 or higher oral mucositis (OM) was 37.5% (three patients) in the TJ - 14 group and 50.0% (four patients) in the placebo group, and there was no significant difference between the two groups. The mean onset day was 9.7 in the TJ - 14 group and 6.7 in the placebo group. The mean duration of grade 2 or higher oral mucositis (OM) was 1.3 days in the TJ - 14 group and 3.7 days in the placebo group. | [4] |
| Patients who underwent docetaxel- or cisplatin-based CT/CRT for EC | Controlled clinical trial | 4 weeks | 39 | TJ-14 (The details not speficied) | 2.5 g / 3 times per day, dissolve 2.5 g of TJ-14 in 50 mL drinking water and rinse oral cavity with the solution 10 seconds | Solution | The incidences of oral mucositis, appetite loss, nausea, constipation, and vomiting did not significantly differ between the TJ-14 group and controls, diarrhea incidence was significantly lower in the TJ-14 group by the second treatment course (P ¼ 0.0261 by per protocol set analyses). | [5] |
| Patients with gastric cancer or colorectal cancer who developed moderate to severe COM (grade ≥1) during any cycle of chemotherapy | two prospective, multi-institutional, randomized, double-blind, placebo-controlled trials | 4-6 weeks | 181 | TJ-14 (The details not speficied) | 2.5 g /3 times per day, dissolve 2.5 g of TJ-14 in 50 mL drinking water and rinse oral cavity with the solution 10 seconds | Solution | Treatment with TJ-14 was associated with marginally significant reduction in the duration of severe grade ≥2 chemotherapy-induced oral mucositis (COM) in comparison to patients receiving placebo indicating the effect of TJ-14 in reducing the severity of COM. | [6] |
| Patients undergoing hematopoietic stem cell transplantation | Randomized | 28 days | 30 | Hangeshashinto (HST), Coptidis Rhizoma, Ginseng Radix, Glycyrrhizae Radix, Pinelliae Tuber, Scutellariae Radix, Zingiberis Rhizoma Processum, and Zizyphi Fructus | 2.5 g HST (TJ-14)/ 3 times per day, dissolved in 40 mL saline solution | Solution | HST use did not clearly inhibit onset of OM but showed a tendency to inhibit OM exacerbation. | [7] |
| Patients with head and neck cancer were subjected to radiation therapy with concomitant administration of anticancer drugs | Controlled clinical trail | 1 month | 50 | Hangeshashinto ( Pinelliae tuber, Scutellariae radix, glycyrrhizae radix, Zizyphi Fructus, ginseng radix, Zin giberis Processum rhizoma, and Coptidis rhizome) | 2.5 g dissolved in 100ml hot water and frozen into ice block | Preparation of frozen | Cryotherapy in combination with Hangeshashinto can be used for the treatment of radiation-induced oral stomatitis. | [8] |

**References**

1. Aoyama T., Nishikawa K., Takiguchi N., Tanabe K., Imano M., Fukushima R., Sakamoto J., Oba M. S., Morita S., Kono T., Tsuburaya, A. Double-Blind, Placebo-Controlled, Randomized Phase II Study of TJ-14 (Hangeshashinto) for Gastric Cancer Chemotherapy-Induced Oral Mucositis. Cancer Chemotherapy and Pharmacology. 2014;73(5):1047-54.
2. Yamashita T., Araki K., Tomifuji M., Kamide D., Tanaka Y., Shiotani, A. A Traditional Japanese Medicine--Hangeshashinto (TJ-14)-Alleviates Chemoradiation-Induced Mucositis and Improves Rates of Treatment Completion. Supportive Care in Cancer: Official Journal of the Multinational Association of Supportive Care in Cancer. 2015;23(1): 29-35.
3. Matsuda C., Munemoto Y., Mishima H., Nagata N., Oshiro M., Kataoka M., Sakamoto J., Aoyama T., Morita S., Kono T. Double-Blind, Placebo-Controlled, Randomized Phase II Study of TJ-14 (Hangeshashinto) for Infusional Fluorinated-Pyrimidine-Based Colorectal Cancer Chemotherapy-Induced Oral Mucositis. Cancer Chemotherapy and Pharmacology. 2015;76(1):97-103.
4. Taira K., Fujiwara K., Fukuhara T., Koyama S., Takeuchi H. The Effect of Hangeshashinto on Oral Mucositis Caused by Induction Chemotherapy in Patients with Head and Neck Cancer. Yonago Acta Medica. 2020;63(3):183-7.
5. Takahashi M., Nakajima M., Muroi H., Satomura H., Domeki Y., Ihara K., Kikuchi M., Kubo T., Yokoyama H., Ogata H., Yamaguchi S., Sasaki K., Hakata K., Kawamata H., Kato H. Prevention of the Chemotherapy-Induced Oral Mucositis in Esophageal Cancer by Use of Hangeshashinto (TJ-14). International Surgery. 2018;103(7-8):401-8.
6. Nishikawa K., Aoyama T., Oba M. S., Yoshikawa T., Matsuda C., Munemoto Y., Takiguchi N., Tanabe K., Nagata N., Imano M., Oshiro M., Fukushima R., Kataoka M., Morita S., Tsuburaya A., Mishima H., Kono T., Sakamoto, J. The Clinical Impact of Hangeshashinto (TJ-14) in the Treatment of Chemotherapy-Induced Oral Mucositis in Gastric Cancer and Colorectal Cancer: Analyses of Pooled Data from Two Phase II Randomized Clinical Trials (HANGESHA-G and HANGESHA-C). Journal of Cancer, 2018;9(10):1725-30.
7. Yoshimatsu M., Kawashita Y., Soutome S., Murata M., Sawayama Y., Kurogi T., Nakao N., Miyazaki Y., Umeda M. Ukai, T. Hangeshashinto for Prevention of Oral Mucositis in Patients Undergoing Hematopoietic Stem Cell Transplantation: A Randomized Phase II Study. Supportive Care in Cancer: Official Journal of the Multinational Association of Supportive Care in Cancer. 2023;31(12):707.
8. Kato T., Sakagami H. Efficacy of Cryotherapy and Hangeshashinto for Radiation-induced Oral Stomatitis: Preliminary Study. In Vivo. 2023;37(2):830-5.

**Supplementary Table 11. Representative clinical studies of BXD on tumor-related conditions**

| **Patients entered** | **Study design** | **Study length** | **Sample size** | **Observation Group** | | | **Main results** | **Reference** |
| --- | --- | --- | --- | --- | --- | --- | --- | --- |
|  |  |  |  | **Medicine Detail** | **Dosage** | **preparation** | **Details** |  |
| Colorectal cancer | Randomized | 60 days | 140 | On the basis of the control group, BXD was added.  Pinelliae Rhizoma 9 g, Zingiberis Rhizoma 6 g, Scutellariae radix 6 g, Ginseng Radix et Rhizoma 6 g, Coptidis Rhizoma 3 g, Glycyrrhizae Radix et Rhizoma Praeparata cum Melle 6 g, 4 pieces of Jujubae Fructus | 2 times/ d, 150ml/ time | Decoction | BXD has certain effects in the treatment of post-operative recovery of colorectal cancer. BXD treatment group was better than the control group in four aspects: clinical efficacy, TCM symptom score, KPS score, and serum CEA level, and the difference was statistically significant (p<0.05). There were no adverse reactions in the safety evaluation of both the treatment group and the control group. | [1] |
| Colon cancer | Randomized | 3 months | 96 | On the basis of conventional chemotherapy, adopt BXD treatment：Codonopsis Radix 15 g, Pinelliae Rhizoma 10 g, Scutellariae radix 10 g, Zingiberis Rhizoma 9 g, Coptidis Rhizoma 6 g, Glycyrrhizae Radix et Rhizoma Praeparata cum Melle 6 g, 5 piece of Jujubae Fructus.  For those with vomiting, add Haematitum 15 g, Inulae Flos 15 g.  For those with anorexia, add Hordei Fructus Germinatus 20 g, Stir-fried Crataegi Fructus 15 g, Stir-fried Messa Medicata Fermentata 15 g.  For those with Qi deficiency, add Astragali Radix 20 g.  For those with blood deficiency, add Asini Corii Colla 15 g, Rehmanniae Radix Praeparata 20 g. | 1 dose/ d | Decoction | BXD has significant efficacy in the treatment of colon cancer. The BXD treatment group was better than the control group in terms of patient clinical symptom scores, clinical efficacy comparison, and recurrence rate during the follow-up period. The difference is statistically significant. | [2] |
| Malignant tumor patients with gastrointestinal reactions caused by chemotherapy drugs | Randomized | 7 days | 90 | Applied BXD on the basis of conventional treatment: Coptidis Rhizoma 6 g, Scutellariae radix 9 g, Zingiberis Rhizoma 9 g, Pinelliae Rhizoma 9 g, Codonopsis Radix 15 g, Glycyrrhizae Radix et Rhizoma Praeparata cum Melle 9 g, Jujubae Fructus 9 g.  For those with Qi deficiency, add Astragali Radix 30 g, Stir-fried Atractylodis Macrocephalae Rhizoma 12 g.  For severe blood deficiency, add Astragali Radix 30 g, Angelicae Sinensis Radix 12 g.  For those with thick and greasy tongue coating and severe vomiting, add Pogostemonis Herba 12 g, Bambusae Caulis in Taenias 15 g.  For those with severe diarrhea, add Stir-fried Atractylodis Macrocephalae Rhizoma 12 g, Stir-fried Coicis Semen 30 g, Euodiae Fructus 6 g.  For those with bloating and Qi stagnation, add Magnoliae Officinalis Cortex 10 g, Aurantii Fructus 10 g.  For those with gastric bloating after stopping eating, add Stir-fried Galli Gigerii Endothelium Corneum 15 g, Messa Medicata Fermentata 10 g, Crataegi Fructus 6 g.  For those with epigastric pain, add Scolopendra 6 g, Corydalis Rhizoma 12 g, Curcumae Radix 10 g.  For those who have difficulty in urinating and suffer from generalized edema, add Stephaniae Tetrandrae Radix 9 g, Stir-fried Atractylodis Macrocephalae Rhizoma 12 g, Zingiberis Rhizoma Recens 10 g.  For those with pleural effusion, add Descurainiae Semen Lepidii Semen 6 g. | 1 dose/ d | Decoction | BXD has a good therapeutic effect on digestive tract reactions caused by cancer chemotherapy. Compared with the control group, the BXD treatment group performed better in terms of nausea and vomiting changes, diarrhea changes, and appetite effects. And the difference is statistically significant. | [3] |
| Malignant tumor | Randomized | 1 week | 72 | Pinelliae Rhizoma 10 g, Scutellariae Radix 10 g, Zingiberis Rhizoma 10 g, Codonopsis Radix 20 g, Glycyrrhizae Radix et Rhizoma Praeparata cum Melle 10 g, Coptidis Rhizoma 3 g, Sepiae Endoconcha 30 g | 1 dose/ d | Decoction | BXD has a good therapeutic effect on gastrointestinal reactions caused by chemotherapy of malignant tumors. The total effective rate of the BXD treatment group in grading nausea, vomiting, and anorexia was higher than that in the control group. Comparing the cassette scores of the two groups, the BXD treatment group had a higher effective rate (77.78%), and the difference was statistically significant compared with the control group (p<0.05). | [4] |
| Esophageal cancer | Randomized | 7 weeks | 26 | Pinelliae Rhizoma Praeparatum cum Zingibere et Alumine 30 g, Scutellariae radix 15 g, Coptidis Rhizoma 9 g, Ginseng Radix et Rhizoma 10 g, Zingiberis Rhizoma 10 g, Glycyrrhizae Radix et Rhizoma Praeparata cum Melle 12 g, 7 pieces of Jujubae Fructus | 2 times/ d | Decoction | BXD has a certain effect on alleviating the side effects of chemotherapy for esophageal cancer. It is effective in gastrointestinal reactions and alleviating bone marrow suppression. The quality of life of patients in the BXD treatment group was better than that of patients with chemotherapy alone. | [5] |
| Gastrointestinal tumors | Randomized | 10 days (from 3 days before chemotherapy to the 6th day of chemotherapy) | 60 | Processed Pinelliae Rhizoma 10 g, Scutellariae radix 10 g, Coptidis Rhizoma10g, Zingiberis Rhizoma 10 g, Glycyrrhizae Radix et Rhizoma Praeparata cum Melle 10 g, Jujubae Fructus 10 g, Pseudostellariae Radix 20 g, Atractylodis Macrocephalae Rhizoma 20 g, Galli Gigerii Endothelium Corneum 20 g, Jiao Sanxian (Fried Hordei Fructus Germinatus, Fried Crataegi Fructus, Fried Messa Medicata Fermentata) 20 g, Corydalis Rhizoma 20 g, Concha Arcae 30 g | 1 dose/ d | Decoction | BXD treatment has a certain effect on relieving severe gastrointestinal reactions, mainly nausea and vomiting, during chemotherapy. The total effective rate of controlling nausea and vomiting and the control rate of abdominal pain in the BXD treatment group after chemotherapy were better than those of the control group (p<0.05). | [6] |

**References**

1. Yingjie L., Shuguang Q., Jun L. Clinical Study on Banxia Xiexin Decoction in Treatment of Disharmony between Liver and Spleen after Colorectal Cancer Surgery. World Chinese Medicine. 2017;12(07):1523-6.
2. Zhigang S. Clinical Study on the Treatment of Colon Cancer by Combination of Acridness Relieving Superficies and Bitterness Expelling Internal Heat Method and Banxia Xiexin Decoction. Asia-Pacific Traditional Medicine. 2017;13(08):125-6.
3. Ruihua G., Yuanxun Q. Clinical Observation on the Treatment of Chemotherapy-Induced Gastrointestinal Reactions by Banxia Xiexin Decoction. Inner Mongolia Journal of Traditional Chinese Medicine. 2019;38(07):2-4.
4. Min C., Jian S., Xutao Z., Qiming M. Clinical Observation on the Treatment of Gastrointestinal Reactions to Chemotherapy for Malignant Tumours with Banxia Xiexin Decoction. Journal of Practical Traditional Chinese Medicine. 2015;31(06):501-2.
5. Guohua Z., Xin Z., Weidong D. Clinical Observation on the Toxicity-Reducing Effect of Banxia Xiexin Decoction in Chemotherapy for Oesophageal Cancer. Traditional Chinese Medicinal Research. 1996;9(02):40-1.
6. Jiangjin H. Clinical Observation on the Prevention and Treatment of Gastrointestinal Reactions Induced by Chemotherapy of Gastrointestinal Tumour with Banxia Xiexin Decoction. Journal of Emergency in Traditional Chinese Medicine. 2010;19(04):581-2.

**Supplementary Table 12. Representative clinical studies of BXD on PCOS**

| **Patients entered** | **Study design** | **Study length** | **Sample size** | **Observation Group** | | | **Main results** | **Reference** |
| --- | --- | --- | --- | --- | --- | --- | --- | --- |
|  |  |  |  | **Medicine Detail** | **Dosage** | **Preparation** | **Details** |  |
| PCOS | Randomized | 6 months | 68 | Pinelliae Rhizoma 9 g, Scutellariae Radix 20 g, Coptidis Rhizoma 10 g, Zingiberis Rhizoma 6 g, Epimedii Folium 30 g, Zingiberis Rhizoma Recens 3 pieces, 5 pieces of Jujubae Fructus.  For those who are upset and irritable andwith dry stools, add Bupleuri Radix 12 g, Rhei Radix et Rhizoma 6 g.  For those with epigastric distension and belching, add Fried Atractylodis Macrocephalae Rhizoma 30 g, Aurantii Fructus Immaturus 15 g.  For those with poor appetite, add Fried Atractylodis Macrocephalae Rhizoma 30 g, Citri Reticulatae Pericarpium 15 g.  For those with light menstruation and hair loss, add Cuscutae Semen 30 g, Lycii Fructus 30 g.  For those with sore waist and weak knees, add 30 g of Eucommiae Cortex and 30 g of Cibotii Rhizomaor.  For those whose stool is not smooth, sticky, and the tongue coating is thick and greasy, add Atractylodis Rhizoma 15 g, Poria 30 g. | 1 dose/ d | Decoction | BXD can improve insulin sensitivity and reproductive endocrine status in patients with polycystic ovary syndrome, and there is a time-dependent relationship. BXD can promote the recovery of spontaneous menstruation and increase the pregnancy rate. Its improving effect may be related to the correction of insulin resistance and reproductive endocrine status, and it has a certain degree of safety.  The experiment evaluated six aspects, namely insulin and blood sugar, female hormones, pregnancy and pregnancy outcomes, menstrual cycle, and liver and kidney function. Among them, except for liver and kidney function ALT, Cr, and BUN indicators that did not change significantly after treatment, other aspects were significantly improved after 3-6 months of treatment. | [1] |
| PCOS | Block randomized | 12 weeks | 62 | Pinelliae Rhizoma 9 g, Scutellariae Radix 30 g, Coptidis Rhizoma15 g, Zingiberis Rhizoma15 g, Codonopsis Radix12 g, Coicis Semen 30 g, Lycii Fructus 30 g. For those with light menstruation and hair loss, add Cuscutae Semen 15 g, Morindae Officinalis Radix 15 g.  For those with sore waist and weak knees, add 30 g of Eucommiae Cortex and 15 g of Corni Fructus.  For those who are upset and irritable, add Bupleuri Radix 15 g, Paeoniae Radix Alba 12 g.  For epigastric distension and belching, add Fried Atractylodis Macrocephalae Rhizoma 15 g, Aurantii Fructus Immaturus 15 g.  For those with thick tongue coating, add Atractylodis Rhizoma 15 g, Magnoliae Officinalis Cortex 12 g, Citri Reticulatae Pericarpium 12 g.  For those with cold lower abdomen, add Zingiberis Rhizoma Praeparatum 20 g. | 2 times/ d, 150ml/ time | Decoction | BXD can effectively improve insulin sensitivity in patients with PCOS and HI, and its effect is equivalent to etformin. BXD can restore spontaneous ovulation and improve oligomenorrhea in patients, and its effect is related to reducing FINS levels.  The experiment evaluated five indicators, namely glucose metabolism indicators, BBT, menstrual cycle and TCM symptom scores, reproductive hormone indicators and safety indicators. Among them, except that the cyclic biphasic rate of BBT and the incidence rate of BBT bipolar in the BXD treatment group were higher than those in the control group (p<0.05), there were no significant statistical differences between the other index treatment groups and the control group after treatment. | [2] |
| PCOS | Randomized | 6 months | 24 | Pinelliae Rhizoma 15 gScutellariae Radix 15 g，Coptidis Rhizoma 6 g，Magnoliae Officinalis Cortex 18 g，Citri Reticulatae Pericarpium12 g，Fried Atractylodis Macrocephalae Rhizoma 15 g, Poria 15 g，Cyperi Rhizoma 10 g，Zingiberis Rhizoma Recens 10 g，Atractylodis Rhizoma 10 g，Aurantii Fructus 10 g, Arisaema Cum Bile 15 g, Messa Medicata Fermentata 15 g，Fried Coicis Semen 30 g，Salt-fried Eucommiae Cortex 15 g，Gleditsiae Spina 15 g，Asari Radix et Rhizoma 9 g.  For those who suffer from insomnia and dreaminess, add Ziziphi Spinosae Semen 15 g and Polygoni Multiflori Caulis 30 g.  For those who are menstruating, add Paeoniae Radix Rubra 15 g and Persicae Semen 10 g. | 2 times/ d, 150ml/ time | Decoction | BXD effectively regulates PCOS sex hormone disorders, and its mechanism of action may be related to regulating the abundance and diversity of intestinal flora, increasing beneficial bacteria, reducing pathogenic bacteria, and restoring intestinal microecological homeostasis.  Compared with the PCOS group, the BXD treatment group could significantly reduce serum LH, T and LH/FSH (follicle-stimulating hormone) ratios in PCOS patients (P<0.05). The BXD treatment group could upregulate the relative abundance of Bacteroidetes, Proteobacteria, Bacteroidetes, Ruminococcum, Lactobacillus, Akkermansia and Sutterella (P<0.05). It also decrease the relative abundance of Firmicutes, Actinobacteria, Prevotella, Lachnospira and Clostridium tenella (P<0.05) | [3] |
| PCOS-IR | Randomized | 3 months | 80 | Processed Pinelliae Rhizoma 10 g，Coptidis Rhizoma 3 g，Scutellariae Radix 10 g，Phellodendri Chinensis Cortex 3 g, Zingiberis Rhizoma 3 g，Citri Reticulatae Pericarpium 6 g，Poria 12 g，Epimedii Folium 15 g，Astragali Radix 10 g, Codonopsis Radix 10 g, Atractylodis Rhizoma 10 g，Angelicae Sinensis Radix 12 g，Honey-fried Cimicifugae Rhizoma 3 g, Bupleuri Radix 3 g，Glycyrrhizae Radix et Rhizoma Praeparata cum Melle 6 g. | 2 times/ d | Decoction | Modified BXD could effectively improve the clinical symptoms of PCOS-IR due to spleen deficiency and stomach heat, increase IL-4 levels, regulate glucose metabolism, and control IR, thereby treating PCOS. The efficacy of TCM syndromes in the 2 groups of patients: the clinical efficacy of the treatment group was significantly better than that of the control group (χ2 = 4.501, P <0.05). Comparison of syndrome scores between the two groups of patients: After treatment, compared with those before treatment, the TCM syndrome scores of the two groups of patients were reduced (P＜0.05); the syndrome scores of the patients in the treatment group were reduced more significantly (P＜ 0.05).  Serum sex hormones of the 2 groups of patients: after treatment, the levels of T and LH in both groups were reduced, and the difference was statistically significant (P>0.05); in comparison between the groups, the reduction of T and LH in the treatment group was more obvious, and the difference was statistically significant (P >0.05), indicating that the treatment group can better improve T and LH. Comparison of patients' glucose metabolism indicators: There was no statistically significant difference in FPG, FINS, and HOMA-IR (P>0. 05). After treatment, the levels of FINS and HOMA-IR in both groups were lower than before treatment, and the treatment group was lower than that of the control group (P<0.05), indicating that both groups could reduce the degree of insulin resistance, and the treatment group had better effects. | [4] |

**References**

1. Dongxue Z., Xinming L., Yiming Z. Time-Effect Relationship Study of Modified Banxia Xiexin Decoction in the Treatment of Polycystic Ovary Syndrome Insulin Resistance (Deficiency of the Spleen and Heat of the Stomach). Global Traditional Chinese Medicine. 2017;10(02):220-4.
2. Xinmin L., Rufeng C., Sheng W., Jinyuan L., Lili Z., Dongxue Z. Efficacy of Modified Banxia Xiexin Decoction in Treating PCOS Hyperinsulinemia of Stomach Heat and Spleen Deficiency. Beijing Journal of Traditional Chinese Medicine. 2022;41(04):431-5.
3. Fenqin Z., Xiaonan D., Mingxia A., Yan Z., Jieying L. Effect of Banxia Xiexin Decoction on Intestinal Microflora Diversity of PCOS Based on 16S rDNA Sequencing. Chinese Journal of Microecology. 2022;34(10):1151-7.
4. Limei W., Xinyu G., Jie C., Jinfen Y., Yan s., Zhe M. Clinical Efficacy of Modified Banxia Xiexin Tangin PCOS-IR Patients with Spleen-deficiency Stomach-heat Type and Effect on Serum IL-4. Journal of Hebei Traditional Chinese Medicine and Pharmacology. 2023;38(06):48-52.

**Supplementary Table 13. Representative clinical trials of BXD on other diseases**

| **Patients entered** | **Study design** | **Study length** | **Sample size** | **Observation Group** | | | **Main results** | **Reference** |
| --- | --- | --- | --- | --- | --- | --- | --- | --- |
|  |  |  |  | **Medicine Detail** | **Dosage** | **Preparation** | **Details** |  |
| Phlegm fire disturbing heart ventricular premature beat | Randomized | 4 weeks | 92 | Pinelliae Rhizoma 10 g, Coptidis Rhizoma 8 g, Scutellariae Radix 9 g, Ginseng Radix et Rhizoma Rubra 10 g, Zingiberis Rhizoma 9 g, Glycyrrhizae Radix et Rhizoma Praeparata cum Melle 8 g, Jujubae Fructus 9 g. | 1 dose/ d,  200ml/ dose,  2 times/ d | Decoction | The combined use of BXD and amiodarone has a good synergistic effect, which can effectively improve the abnormal state of patients with ventricular premature beats and enhance the overall clinical efficacy without increasing drug toxicity and side effects. The effective rate was 89.13% in the treatment group and 71.74% in the control group. | [1] |
| Chronic subjective dizziness | Randomized | 4 weeks | 60 | Pinelliae Rhizoma 10 g, Scutellariae Radix 9 g, Zingiberis Rhizoma 9 g, Ginseng Radix et Rhizoma 9 g, Coptidis Rhizoma 3 g, Jujubae Fructus 10 g, Glycyrrhizae Radix et Rhizoma Praeparata cum Melle 9 g. | 400mL/ dose, 200mL/ time,  2 times/d. | Decoction | BXD can alleviate the clinical symptoms of patients with chronic subjective dizziness. DHI score and TCM syndrome score of BXD were significantly higher. | [2] |
| ventilator-associated pneumonia | Randomized | not mentioned | 52 | Pinelliae rhizome 10 g, Scutellariae Radix 9 g, Zingiberis Rhizoma 6 g, Ginseng Radix et Rhizoma 6 g, Coptidis Rhizoma 6 g, Glycyrrhizae Radix et Rhizoma Praeparata cum Melle 6 g and 4 pieces of Jujubae Fructus. | 1 dose/ d | Decoction | The 5-day VAP incidence and cumulative incidence in the BXD treatment group were lower than those in the control group. The offline success rate was higher than that of the control group. The mechanical ventilation time and ICU stay time in the prevention group were shorter than those in the control group. | [3] |
| AIDS-related diarrhea | Randomized | 14 days | 70 | Pinelliae Rhizoma Praeparatum 20 g, Scutellariae Radix 10 g, Coptidis Rhizoma 6 g, Zingiberis Rhizoma 12 g, Codonopsis Radix 15 g, 6 pieces of Jujubae Fructus, Glycyrrhizae Radix et Rhizoma 6 g.  For patients with severe nausea and vomiting, change Pinelliae Rhizoma Praeparatum to Pinelliae Rhizoma Praeparatum cum Zingibere et Alumine and add ginger Bambusae Caulis in Taenias, Inulae Flos and Haematitum.  For patients with severe bloating, add Stir-fried Raphani Semen, Arecae Semen, Aucklandiae Radix and Citri reticulatae.  For patients with abdominal pain, add Stir-fried Paeoniae Radix Alba and Vinegar-fried Corydalis Rhizoma.  For patients with anal burning, add Pulsatillae Radix and Portulacae Herba, and change Coptidis Rhizoma to 12 g.  For patients with watery stools, add Stir-fried Coicis Semen, Atractylodis Macrocephalae Rhizoma, Atractylodis Rhizoma and Poria.  For patients with sticky stool, add Arecae Semen and Aucklandiae Radix. | not mentioned | Decoction | BXD has a good therapeutic effect in treating AIDS-related diarrhea and is worthy of clinical application. The total effective rate of clinical treatment was 87.1% | [4] |
| Damp-heat cough | Randomized | not mentioned | 50 | Pinelliae Rhizoma, Scutellariae Radix, Zingiberis Rhizoma, Coptidis Rhizoma, Glycyrrhizae Radix et Rhizoma, Coicis Semen, Caulis Phragmitis, Mori Cortex, Trichosanthis Pericarpium, Lycii Cortex, Benincasae Semen, Acori Tatarinowii Rhizoma.  For patients with severe mouth bitterness, add Prunellae Spica and Gardenia Jasminoides.  For patients with anorexia, add Magnoliae Officinalis Cortex, Crataegi Fructus.  For patients with yellow urine, add Artemisiae Scopariae Herba and Talci Pulvis. | 1 dose/ d,  2 times/ d. | Decoction | The total effective rate was 96%. | [5] |
| Acute gastroenteritis | Randomized | 5 days | 82 | Pinelliae Rhizoma Praeparatum cum Zingibere et Alumine 12 g, Scutellariae Radix 9 g, Ginseng Radix et Rhizoma 9 g, Zingiberis Rhizoma 9 g, Glycyrrhizae Radix et Rhizoma Praeparata cum Melle 9 g, Coptidis Rhizoma 3 g, Jujubae Fructus 4 pieces.  For patients with severe abdominal pain and distension, add Magnoliae Officinalis Cortex 10 g, Perillae Caulis 10 g, Poria 10 g and Zingiberis Rhizome Recens 10 g.  For patients with severe nausea and vomiting, add Zingiberis Rhizome Recens 6 g.  For patients with indigestion, add Massa Medicata Fermentata 8 g, Stir-fried Hordei Fructus Germinatus and Stir-fried Setariae Fructus Germinatus 8 g and Charred Crataegi Fructus 8 g. | 1 dose/ d,  150ml/ dose,  3 times/ d | Decoction | BXD can significantly alleviate clinical symptoms, restore gastrointestinal function and reduce inflammatory response in patients with acute gastroenteritis. The scores of diarrhea, abdominal pain, watery stools, vomiting and fever were significantly lower in the study group than in the control group. Motilin level was higher than before treatment, gastrin level was lower than before treatment, and the study group was better than the control group. The levels of IL-6, IL-8 and TNF-α in the patients were significantly lower than before treatment, and the study group was lower than the control group (P<0.05). | [6] |
| Gastrointestinal dysfunction | Randomized | not mentioned | 35 | Pinelliae Rhizoma 10~15 g, Coptidis Rhizoma 10~15 g, Scutellariae Radix 10~15 g, Glycyrrhizae Radix et Rhizoma 6~10 g, Coptidis Rhizoma 6~10 g, Zingiberis Rhizoma 6~10 g, Jujubae Fructus 15 g.  For patients with poor appetite, add Crataegi Fructus, Citri Reticulatae Pericarpium.  For patients with severe diarrhea, add Plantaginis Semen, Akebiae Caulis.  For patients with severe vomiting, add Haematitum, Bambusae Caulis in Taenias.  For patients with severe epigastric pain, add Corydalis Rhizoma and Arcae Concha. | 1 dose/ d,  400 ml/ dose,  2 times/ d. | Decoction | The total effective rate was 97%. | [7] |
| Impaired fasting blood glucose | Randomized | not mentioned | 120 | Pinelliae Rhizoma Praeparatum cum Alumine 10~12 g, Ginseng Radix et Rhizoma 10~12 g, Scutellariae Radix 10~15 g, Coptidis Rhizoma 10~30 g, Hirudo 10~20 g, Zingiberis Rhizoma 10~20 g, Glycyrrhizae Radix et Rhizoma 10~15 g, 6 pieces of Jujubae Fructus. | 1 dose/ d,  300 mL/ dose,  2 times/ d. | Decoction | After treatment, fasting blood glucose (FPG), sugar load 2h blood glucose, (2hPG), triglyceride (TG) and total cholesterol (TC) were all reduced to varying degrees, and there was basically no change in the above aspects in the control group. | [8] |
| Chronic cough | Randomized | 7 days | 132 | Pinelliae rhizome 10 g, Codonopsis Radix 10 g, Asteris Radix et Rhizoma 10 g, Stemonae Radix 10 g, Cynanchi Stauntonii Rhizome et Sadix 10 g, Perillae Caulis 10 g, Citri Reticulatae Pericarpium 10 g, Coptidis Rhizoma 6 g, Glycyrrhizae Radix et Rhizoma Praeparata cum Melle 6 g, Platycodonis Radix 6 g, Zingiberis rhizome 5 g, Scutellariae Radix 5 g, Processed Euodiae Fructus 3 g.  For patients with severe cough symptoms, add Honey-fried Eriobotryae Folium, Farfarae Flos, Armeniacae Semen Amarum, Fritillariae Thunbergia Bulbus, Peucedani Radix.  For patients with severe phlegm-dampness, add Citri Exocarpium Rubrum and Atractylodis Rhizoma.  For patients with visceral Qi obstruction, add Rhei radix et rhizome, Aurantii Fructus Immaturus and Magnoliae Officinalis Cortex.  For patients with chronic cough, add Schisandrae Chinensis Fructus. | 1 dose/ d,  2 times/ d. | Decoction | BXD has a significant effect in treating patients with chronic cough and can effectively improve the clinical symptoms of patients. The effective rates of study group and control group were 92.4% and 72.7%, respectively. | [9] |
| Gastrointestinal disfunction | Randomized | not mentioned | 70 | Pinelliae Rhizoma 10~15 g, Coptidis Rhizoma 10~15 g, Scutellariae Radix 10~15 g, Glycyrrhizae Radix et Rhizoma 6~10 g, Coptidis Rhizoma 6~10 g, Zingiberis Rhizoma 6~10 g, Jujubae Fructus 15 g.  For patients with poor appetite, add Crataegi Fructus, Citri Reticulatae Pericarpium.  For patients with severe diarrhea, add Plantaginis Semen, Akebiae Caulis. For patients with severe vomiting, add Haematitum, Bambusae Caulis in Taenias.  For patients with severe epigastric pain, add Corydalis Rhizoma and Arcae Concha. | 1 dose/ d,  400 ml/ dose,  2 times/ d. | Decoction | BXD can effectively treat gastrointestinal disorders. The total effective rate was 85.7%. | [10] |
| Sleep disorder | Randomized | 2~4 weeks | 92 | Pinelliae Rhizoma Praeparatum cum Zingibere et Alumine 15 g, Codonopsis Radix 20 g, Coptidis Rhizoma 6 g, Glycyrrhizae Radix et Rhizoma 6 g, Zingiberis Rhizoma 6 g, Scutellariae Radix 10 g, Jujubae Fructus 10 g, Polygalae Radix 10 g, Albiziae Cortex 30 g.  For patients with headaches, add Chuanxiong Rhizoma 10 g and Saposhnikoviae Radix 10 g.  For patients with backache, add Cibotii Rhizoma 10 g and Eucommiae Cortex 10 g.  For patients with loss of appetite, add Atractylodis Macrocephalae Rhizoma 15 g, Stir-fried Hordei fructus germinates 10 g and Stir-fried Massa Medicata Fermentata 10 g.  For patients with acid reflux, add Euodiae Fructus 3 g and Calcined Ostreae Concha 30 g.  For patients with excessive phlegm and nausea, add Arisaematis Rhizoma Preparatum 10 g and Bambusae Caulis in Taenias 10 g.  For patients with dizziness, add Gastrodiae Rhizoma 9 g and Prunellae Spica 20 g.  For patients with irritability, add Polygonati Odorati Rhizoma 10 g and Ophiopogonis Radix 10 g.  For patients with dry stool, add Cannabis Fructus 10 g and fried Aurantii Fructus 10 g.  For patients with Qi deficiency and fatigue, add Astragali Radix 10 g and Schisandrae Chinensis Fructus 10 g. | 1 dose/ d,  2 times/ d. | Decoction | BXD relieve sleep disorders in patients with epigastric discomfort. The effective rates of study group and control group were 89.13% and 56.52%, respectively. | [11] |
| Chronic stomach disease | Randomized | 4 weeks | 96 | Pinelliae Rhizoma Praeparatum cum Zingibere et Alumine 10 g, Codonopsis Radix 9 g, Scutellariae Radix 9 g, Glycyrrhizae Radix et Rhizoma Praeparata cum Melle 6 g, Zingiberis Rhizoma 6 g, Coptidis Rhizoma 6 g, 4 pieces of Jujubae Fructus.  For patients with epigastric fullness and dry stools, reduce Scutellariae Radix, add Aurantii Fructus Immaturus 20 g, Citri Reticulatae Pericarpium 30 g, Rhei Radix et Rhizoma 10 g, Magnoliae Officinalis Cortex 15 g.  For patients with epigastric fullness, borborygmus and diarrhea, anorexia, reduce Scutellariae Radix, add Dioscoreae Rhizoma 30 g, Atractylodis Macrocephalae Rhizoma 30 g, Coicis Semen 30 g, Epimedii Folium 30 g, Citri Reticulatae Pericarpium 30 g, Nelumbinis Semen 20 g.  For patients with epigastric fullness, borborygmus and diarrhea in the morning, reduce Scutellariae Radix, add Psoraleae Fructus 20 g, Myristicae Semen 20 g, Schisandrae Chinensis Fructus 15 g.  For patients with epigastric fullness, anorexia, insomnia and dry stools, add Bambusae Caulis in Taenias 20 g, Aurantii Fructus Immaturus 20 g and Citri Reticulatae Pericarpium 30 g. | 1 dose/d,  400 mL/ dose,  2 times/ d. | Decoction | BXD has a definite clinical efficacy in treating chronic gastritis and can effectively shorten the time it takes for clinical symptoms to disappear. The total effective rate was 97.92% in the treatment group and 85.42% in the control group. The mean time of clinical symptom disappearance was (6.58±1.72) d in the treatment group and (10.87±2.31) d in the control group. | [12] |

**References**

1. Li Z. Clinical Observation on The Treatment of Ventricular Premature Beats with Phlegm-Fire Confusing Heart Based on Ascending-Descending Theory in Banxia Xiexin Decoction. Yunnan Journal of Traditional Chinese Medicine and Materia Medica. 2021;42(01):33-5.
2. Qiaoling L., Juguang S. Clinical Observation of Banxia Xiexin Decoction in Treating 60 Cases of Chronic Subjective Dizziness. Clinical Journal of Traditional Chinese Medicine, 2020;23(06):1109-12.
3. Zhanglian J. Clinical Observation on the Prevention of Ventilator Associated Pneumonia by Banxia Xiexin Decoction. New Chinese Medicine. 2021;44(09):22-4.
4. Jianshe G., Baitao G. Clinical Observation of AIDS-Related Diarrhea Treated with Banxia Xiexin Decoction. Acta Chinese Medicine. 2011;26(08):899-900.
5. Yanling C. Clinical Observation of Banxia Xiexin Decoction in Treating 50 Cases of Dampness Heat Cough. Modern Journal of Integrated Traditional Chinese and Western Medicine. 2000;9(18):1805-6.
6. Changyi X. Clinical Effect of Banxia Xiexin Decoction in the Treatmient of Acute Gastroenteritis. Clinical Research and Practice. 2022;7(06):139-141.
7. Wei Y. Clinical Observation of Banxia Xiexin Decoction in Treating 35 Cases of Gastrointestinal Dysfunction. China Practical Medicine. 2009;4(17):141-2.
8. Yanping J., Xujian Z. Clinical Efficacy Observation on the Treatment of Impaired Fasting Glucose with Banxia Xiexin Decoction. Journal of Practical Diabetology. 2020;16(06):49-50.
9. Zhendong Y. Clinical Observation on Treating Chronic Cough in TCM. Clinical Journal of Chinese Medicine. 2014;6(14):122-3.
10. Congrong Y. Exploring the Clinical Efficacy of Banxia Xiexin Decoction on Gastrointestinal Dysfunction. Journal of North Pharmacy. 2011;8(01):80.
11. Liqi F., Pingsheng Y., Gangcheng Y. Clinical Observation of Banxia Xiexin Decoction in Treating 46 Cases of Sleep-Disorder. Zhejiang Journal of Traditional Chinese Medicine. 2019;54(09):644.
12. Xiying C. Clinical Observation on Treating Chronic Gastritis in Banxia Xiexin Decoction. Guangming Journal of Chinese Medicine. 2017;32(23):3418-20.
13. Lu H., Qin J., Han N., Xie F., Gong L., Li C. Banxia Xiexin Decoction is Effective to Prevent and Control Irinotecan-Induced Delayed Diarrhea in Recurrent Small Cell Lung Cancer. Integrative Cancer Therapies. 2018;17(4):1109-14.
14. Mori K., Kondo T., Kamiyama Y., Kano Y., Tominaga K. Preventive Effect of Kampo Medicine (Hangeshashin-to) Against Irinotecan-Induced Diarrhea in Advanced Non-Small-Cell Lung Cancer. Cancer Chemotherapy and Pharmacology. 2003;51(5):403-6.
15. Murai T., Matsuo M., Tanaka H., Manabe Y., Takaoka T., Hachiya K., Yamaguchi T., Otsuka S., Shibamoto Y. Efficacy of Herbal Medicine TJ-14 for Acute Radiation-Induced Enteritis: A Multi-Institutional Prospective Phase II Trial. Journal of Radiation Research. 2020;61(1):140-5.
16. Li K., Xu G., Liu C., Zhu B., Liu R., Hua B., Zhang W., Feng X. Effect of a Modified Banxia Xiexin Decoction Plus Chemotherapy on Stage Ⅲ Colon Cancer. Journal of Traditional Chinese medicine. 2019;39(2):251-7.
17. Zhang Z., Wu C., Liu N., Wang Z., Pan Z., Jiang Y., Tian J., Sun M. Modified Banxiaxiexin Decoction Benefitted Chemotherapy in Treating Gastric Cancer by Regulating Multiple Targets and Pathways. Journal of Ethnopharmacology. 2024;331:118277.
18. Gou Y., Zhou H. Observation on the Therapeutic Effect of Chemotherapy Combined with Banxia Xiexin Decoction on Duodenal Cancer under CT Enhanced Scanning. International Journal of Radiation Research. 2023;21(4):633-7.
19. Sin S. H., Wu J., Kang Y., Yip K. H. K., Kong N. S., Wan H., Ng B. F. L., Chen H. Efficacy of Modified Banxia Xiexin Decoction in the Management of Wei-Pi Syndrome (Postprandial Distress Syndrome): Study Protocol for a Randomized, Waitlist-Controlled Trial. Trials. 2021;22(1):135.
20. Wang L., Ke J., Wang C., Li Y., Wu G., Ding Q., Luo Q., Cai R., Lv P., Song T., Xiong S. Efficacy and Safety of Banxia XieXin Decoction, a Blended Traditional Chinese Medicine, as Monotherapy for Patients with Advanced Hepatocellular Carcinoma. Integrative Cancer Therapies. 2020;19.
21. Kuwamura A., Komasawa N., Kori K., Tanaka M., Minami T. Preventive Effect of Preoperative Administration of Hange-Shashin-To on Postoperative Sore Throat: A Prospective, Double-Blind, Randomized Trial. Journal of Alternative and Complementary Medicine. 2015;21(8):485-8.
22. Ichiki M., Wataya H., Yamada K., Tsuruta N., Takeoka H., Okayama Y., Sasaki J., Hoshino, T. Preventive Effect of Kampo Medicine (Hangeshashin-to, TJ-14) Plus Minocycline Against Afatinib-Induced Diarrhea and Skin Rash in Patients with Non-Small Cell Lung Cancer. OncoTargets and Therapy. 2017;10:5107-13.


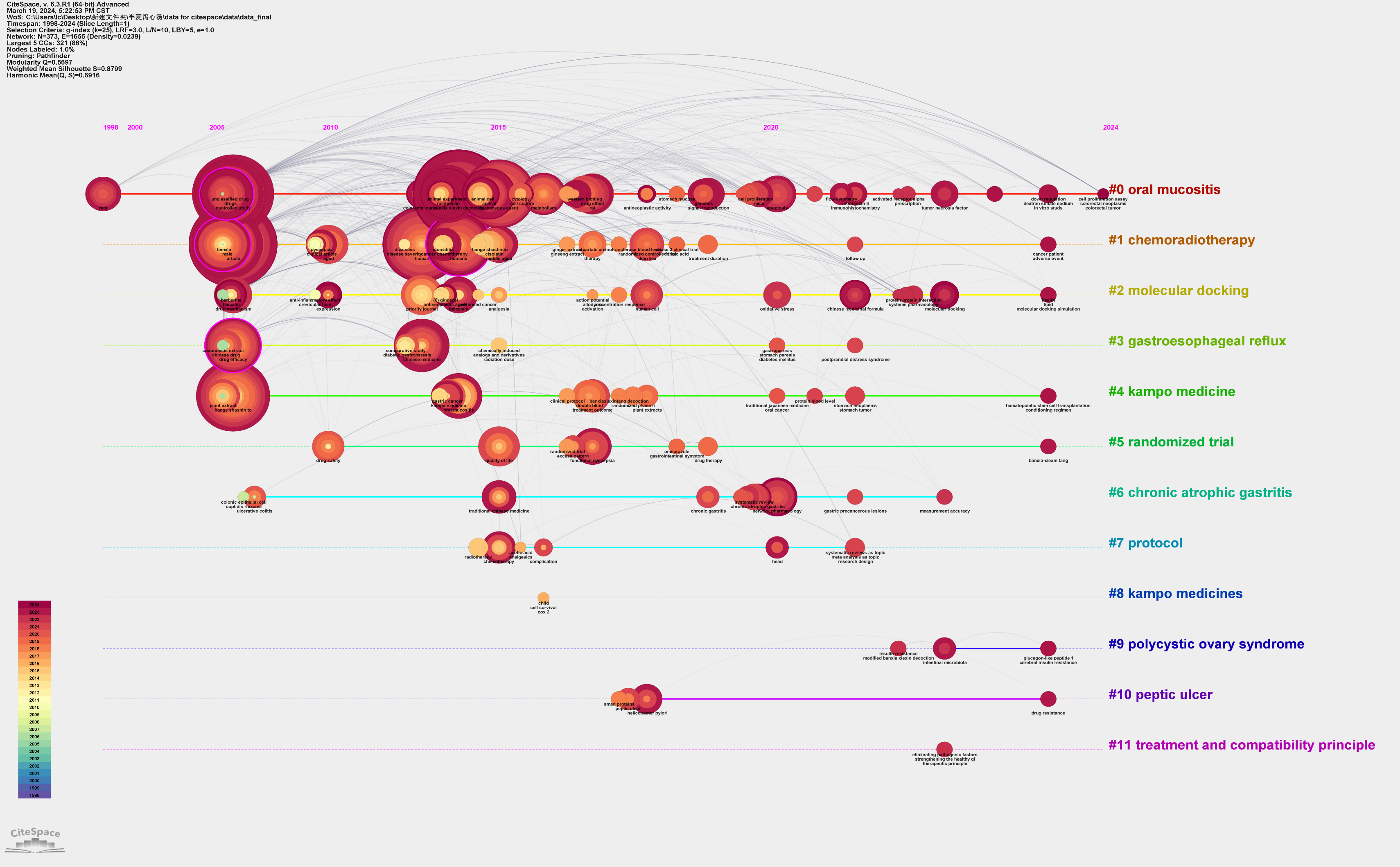


**Figure S1.** Keywords timeline cluster of BXD from Wos, Pubmed, and Scopus.


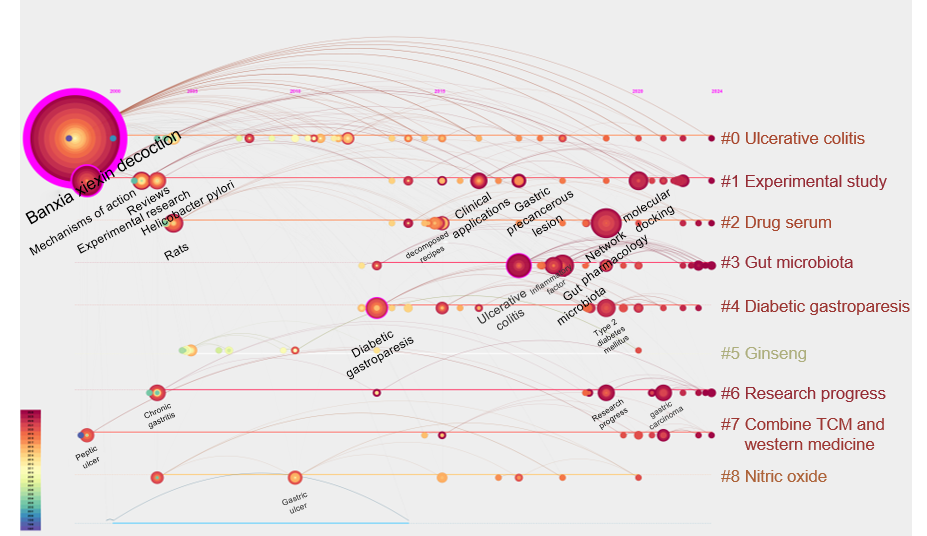


**Figure S2.** Keywords timeline cluster of BXD from CNKI.
